# Oleanane-Type Glycosides with α‑Glucosidase Inhibitory Activity from *Chenopodium serotinum* L., an Everlasting Wild Vegetable

**DOI:** 10.1021/acs.jafc.5c12681

**Published:** 2025-11-17

**Authors:** Chien-Yi Chen, Sheau-Ling Ho, Sheng-Fa Tsai, Ju-Fang Liu, Shoei-Sheng Lee, Chia-Chuan Chang

**Affiliations:** a School of Pharmacy, College of Medicine, 33561 National Taiwan University, Taipei 10050, Taiwan, Republic of China; b Department of Chemical & Materials Engineering, 25795 Chinese Culture University, Taipei 11114, Taiwan, Republic of China; c School of Oral Hygiene, College of Oral Medicine, 38032 Taipei Medical University, Taipei 11031, Taiwan, Republic of China

**Keywords:** *Chenopodium serotinum*, Chenopodiaceae, olean-12-en-11-one glycosides, virtual screening, α-glucosidase inhibition

## Abstract

*Chenopodium serotinum* L. (Chenopodiaceae) is a folk medicine first recorded in Shi Jing (an ancient Chinese book of classic songs) with activities such as antipyretic and detoxification. To explorer the unknown species, the MeOH extract of its aerial part was isolated via various chromatographic techniques (e.g., such as Sephadex LH-20 column chromatography and HPLC) to give seven new oleanane-type triterpenoid glycosides, i.e., chenoponins A–G, isolated from the BuOH fraction. Six of them are rare 3-*O*-trioside, composed of one sophorosyl (i.e., Glc-(1→2)-Glc) and one arabinosyl moiety, which are otherwise known only from a single congeneric species, thereby providing more definitive evidence of their structural novelty within the genus. Their structures were elucidated on the basis of extensive spectroscopic analysis (1D/2D NMR, MS). Virtual screening of these compounds on α-glucosidase by molecular docking followed by an *in vitro* assay on the major ones indicated that chenoponin A (**1**) showed moderate activity against α-glucosidase with IC_50_ values of 23.25 ± 1.00 μM.

## Graphical abstract

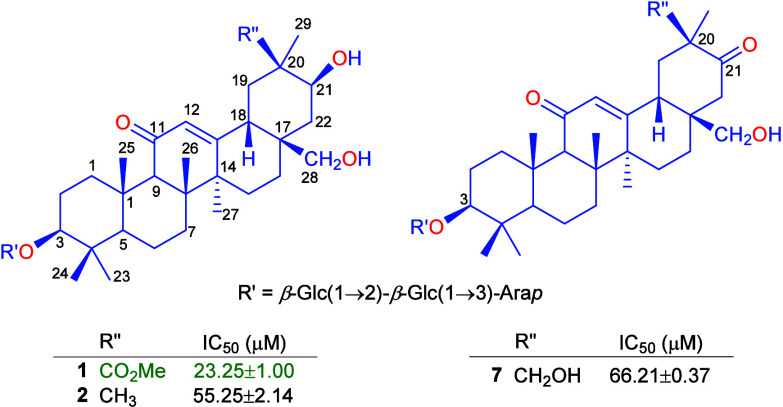

## 1 Introduction

*Chenopodium serotinum* L., trivial name “small goosefoot”, is a green annual herb, distributed widely through Europe, Japan, mainland China, and Taiwan.^1^ It flourishes along gardens, roads, and wild fields. Moreover, its rapid growth has made it become invasion species in Northeast China.^2^ It has been recorded as a wild edible vegetable in the Shi Jing (Classic of Poetry), a collection of ancient Chinese songs around BC 1100, and as folk medicine for detoxification, antipyretic, and anticonstipation.^3^

The related species, *Chenopodium formosanum*, *C. album*, and *C. quinoa*, are known as pseudocereal crops and have shown anticancer, anti-inflammatory, and antidiabetic activities.^4–6^ Bioactive chemical constituents of some *Chenopodium* plants, such as *C. ambrosioides*herb,^3^ *C. album* leaf,^7^ and *C. quinoa* seed,^8^ have been reported. In particular, seeds of *C. quinoa*, an edible grain of South America, contain oleanane-type bisdesmosides at the C-3 and C-28 positions possessing phytolaccagenic acid (Figure 1) as the common aglycon.^8^ The chemical constituents of *C. serotinum*, however, has not been reported yet. As the juice of the fresh herb possesses saponin’s property, i.e., foam forming while stirring, it might contain related saponins as those in *C. quinoa*seeds. Hence, this study focused on the polar constituents from edible *C. serotinum*. Herein, we report the isolation and structural elucidation of 12 compounds, including seven novel saponins (chenoponins A–G) from the *n*-butanol-soluble fraction of the title plant. These compounds were subjected to virtual screening against a target enzyme via molecular docking studies followed by *in vitro* α-glucosidase assays of the major isolates.

**Figure 1:**
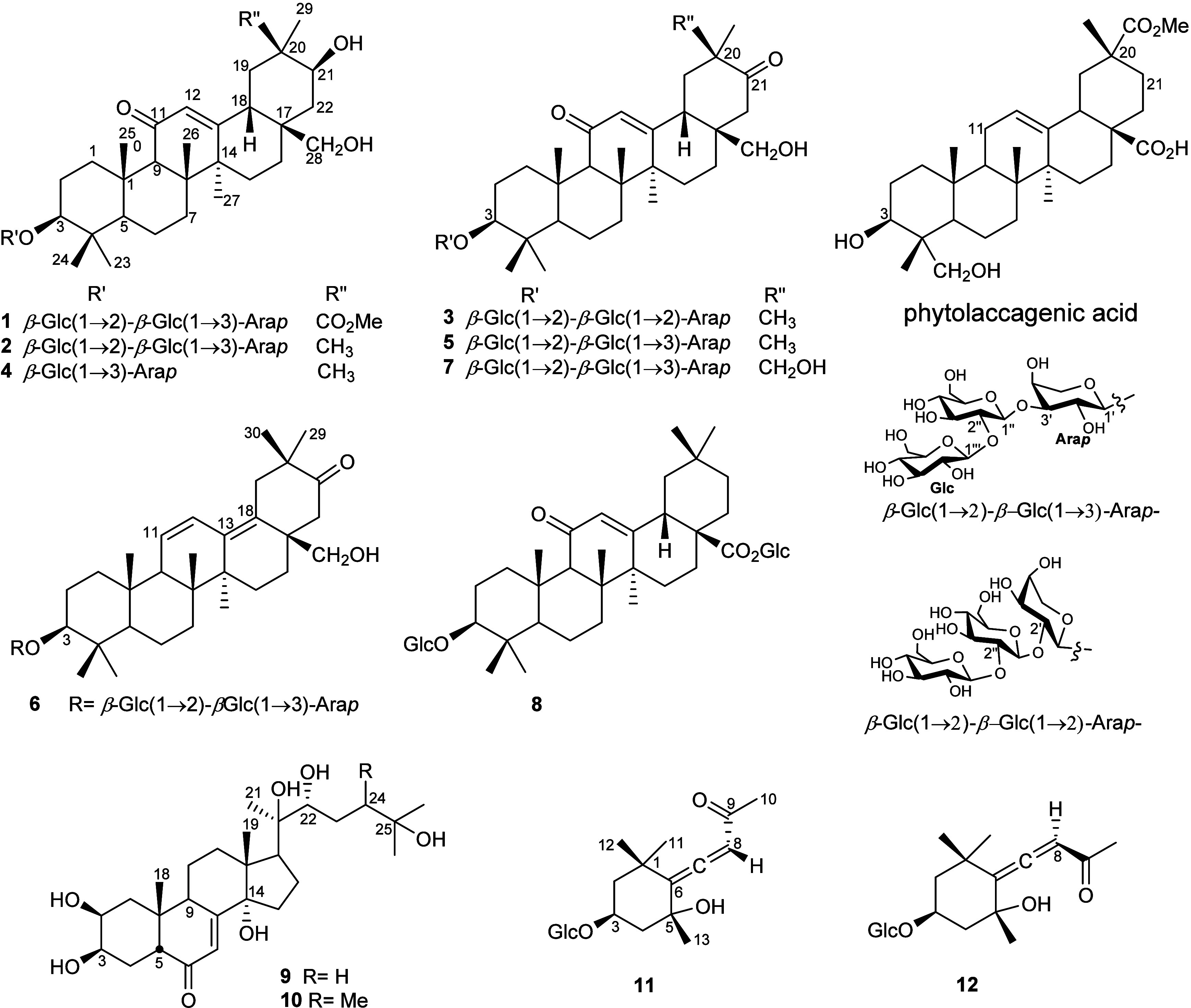
Structure of compounds **1**–**12** from the aerial part of *Chenopodium serotinum* Linn.

## 2 Material and Methods

### 2.1 Instrumentation

The following instruments were used: IR spectra (KBr disc): Jasco FT/IR- 410; Optical rotations (MeOH): Jasco P-2000 polarimeter (Hachioji, Tokyo); UV spectra (MeOH): Hitachi U-2900 double-beam spectrophotometer (Hitachi, Japan); Electron circular dichroic spectra (ECD) (MeOH): Jasco J-720 spectropolarimeter (Hachioji, Tokyo) was used under the following settings: step resolution 0.1 nm, scan speed 50 nm/min, response 0.25s, and sensitivity 200 mdeg; 1D and 2D NMR spectra (CD_3_OD, δ_H_ 3.30 and δ_C_ 49.0 ppm): Bruker AV-400 and AV III-600 NMR spectrometers; HPLC: Agilent 1100 liquid chromatograph (Waldbronn, Germany), Phenomenex Prodigy ODS3 (100 Å; 250 × 4.6 mm, 5 μm) analytical column or Phenomenex Prodigy ODS3 (100 Å; 250 × 10 mm, 5 μm) semipreparative column with a flow rate of 2.2 mL/min and monitored at 254 nm, coupled with a diode array detector (DAD, G1315A; Agilent Technologies, Canada); Column chromatography: Lobar, Lichrospher RP-18 (size A, 240 × 10 mm; size B, 310 × 25 mm; size C, 440 × 37 mm, LiChroprep RP-18, 40–63 μm, Merck, Darmstadt, Germany); ESIMS (electrospray ionization mass spectrometry): Esquire 2000 mass spectrometer (Bruker Daltonics, Germany) and MicroOTOF orthogonal ESI-TOF mass spectrometer (Bruker); TLC analysis: silica gel plates (KG60-F254, Merck).

### 2.2 Plant Material

The aerial part of *Chenopodium serotinum* (C.) L. was collected from Puzi Township, Chiayi County, Taiwan in February, 2020. The voucher specimen was identified by the author (S.S.L.).

### 2.3 Extraction and Isolation

The dry milled aerial part (1.0 kg) of *C. serotinum* immersed in methanol (MeOH) (3 × 6 L) was stirred at room temperature for 24 h and an additional 1 h at 45 °C and then filtered. The filtrate was condensed under reduced pressure at 40 °C to give the MeOH extract (136.9 g). The suspension of the methanol extract (105.0 g) in distilled water (900 mL) was partitioned with dichloromethane, ethyl acetate, and *n*-butanol (H_2_O saturated) in sequence, each 3 × 900 mL, to give the corresponding fractions soluble in CH_2_Cl_2_ (31.3 g), EtOAc (1.8 g), *n*-BuOH (9.5 g), and H_2_O (58.4 g) after condensation. The *n*-BuOH-soluble fraction (9.5 g) was dissolved in MeOH (400 mL), and the solution was filtered. The filtrate was concentrated under reduced pressure to afford a residue (6.25 g), which was subjected to column chromatography on Sephadex LH-20 (6.8 cm × 43.3 cm) eluted with MeOH to yield eight fractions (Frs. 1–8).

Fr. 2 (200 mg out of 315 mg) upon fractionation over a Sephadex LH-20 column (1.1 cm × 64.0 cm; MeOH – H_2_O 8:2) gave Frs. 2–1 to 4. Fr. 2–3 upon separation over a Lobar RP-18 column (size A), eluted using MeOH–H_2_O (5:95 to 50:50) with a flow rate of 2.2 mL/min, yielded **8** (3.2 mg).

Fr. 3 (2.18 g out of 2.60 g) upon separation over a Lobar RP-18 column (size C), eluted using CH_3_CN–0.1% HOAc _(aq)_ 25:75 with a flow rate of 5.0 mL/min and monitored by TLC, afforded Frs. 3–1 to 8. Fr. 3–2 (528 mg out of 537 mg) upon separation over a Lobar RP-18 column (size B), eluted by CH_3_CN – 0.1% HOAc _(aq)_ (9:91) with a flow rate of 2.2 mL/min and monitored by TLC, gave Frs. 3–2–1 to 6, of these Fr. 3–2–2 was **9** (79.0 mg). Fr. 3–2–1 (8 × 27.9 mg) upon separation over a semipreparative RP-18 HPLC column, eluted using CH_3_OH–0.05% trifluoroacetic acid (TFA) _(aq)_ (24:76), afforded **11** (10.0 mg; *t*_R_ = 36.7 min) and **12** (1.6 mg; *t*_R_ = 56.3 min). Fr. 3–2–5 upon separation over the semiprep. RP-18 HPLC column, eluted by CH_3_OH – 0.1% HOAc _(aq)_ (38:62), provided **10** (3.3 mg; *t*_R_ = 47.5 min). Fr. 3–4 (6 × 13.4 mg) upon separation over the semiprep. RP-18 HPLC column, eluted with CH_3_CN–0.1% HOAc _(aq)_ (21:79), yielded **7** (12.4 mg, *t*_R_ = 63.2 min). Fr. 3–7 (7 × 29.7 mg) upon separation over the semiprep. RP-18 HPLC column, eluted using CH_3_CN–0.1% HOAc _(aq)_ (25:75), provided **1** (51.4 mg; *t*_R_ = 33.7 min), **2** (15.9 mg; *t*_R_ = 53.3 min), **3** (7.8 mg; *t*_R_ = 58.4 min), and **4** (7.2 mg; *t*_R_ = 63.8 min). Fraction 3–7–7 upon separation over the semiprep. RP-18 HPLC column, eluted using CH_3_CN–0.1% HOAc _(aq)_ (30:70) for 30 min gave **5** (4.8 mg; *t*_R_ = 28.6 min), then the amount of CH_3_CN to CH_3_CN −0.1% HOAc _(aq)_ (37:63) in 31 min was increased to give **6** (1.5 mg). Fr. 3–8 (220 mg), following procedures similar to those described for Fr.3–7, and yielded another crop of **2** (3.2 mg), **3** (3.1 mg), **4** (3.7 mg), **5** (4.8 mg), and **6** (1.0 mg).

#### Chenoponin A: Methyl 21β,28-Dihydroxy-18β-glycyrrhetinoate

##### 3β-*O*-[β-Sophorosyl-(1→3)]-α-l-arabinopyranoside **(1)**

IR (KBr): 3417, 2929, 2875, 1719, 1644, 1456, 1356, 1259, 1212, 1158, 1134, 1076, 1032, 773, 728, 632, 616 cm^–1^; [α]_D_^25^ + 84.8 (*c* 0.1, MeOH); UV (MeOH) λ_max_ (log ε) 248.0 (3.97); CD (*c* 1.03 × 10^–5^ M, MeOH) [θ]_223.2_ 20384.4, [θ]_265.0_ −27172.2; ^1^H, ^13^C NMR data, see Tables 1 and 2; NOESY and HMBC data, see Table S1; (−)-HRESIMS *m*/*z* 971.4857 [M–H]^−^ (calcd. for C_48_H_75_O_20_, 971.4857, Err = 3.1 ppm).

**Table 1:** ^1^H and ^13^C NMR Data of Aglycon Moieties of Compounds **1**–**7** (CD_3_OD)^a^

	**1** ^a^		**2** ^a^		**3** ^a^		**4** ^a^		**5** ^a^		**6** ^a^		**7** ^b^	
pos.	δ_H_ (*J* in Hz)	δ_C_	δ_H_ (*J* in Hz)	δ_C_	δ_H_ (*J* in Hz)	δ_C_	δ_H_ (*J* in Hz)	δ_C_	δ_H_ (*J* in Hz)	δ_C_	δ_H_ (*J* in Hz)	δ_C_	δ_H_ (*J* in Hz)	δ_C_
1	1.00 (ax) 2.69 (dt, 13.4, 3.6) (eq)	40.3	0.98 (ax) 2.68 (dt, 13.6, 3.6) (eq)	40.3	1.01 (ax) 2.69 (dt, 13.5, 3.4) (eq)	40.2	0.98 (ax) 2.68 (dt, 13.6, 3.8) (eq)	40.3	0.98 (ax) 2.68 (dt, 13.6, 3.8) (eq)	40.2	1.04	39.3	0.96 (ax) 2.66 (dt, 13.4, 3.6) (eq)	40.2
											1.89			
2	1.11	26.9	1.13	26.9	1.23	26.9	1.15	26.9	1.15	26.9	1.76	27.0	1.12	27.0
	1.75		1.77		1.81		1.77		1.80		1.91		1.77	
3	3.16 (dd, 13.4, 4.7)	90.4	3.16 (dd, 11.8, 4.6)	90.4	3.18 (dd, 10.9, 5.3)	90.9	3.16 (dd, 12.6, 4.7)	90.2	3.16 (dd, 11.6, 4.7)	90.9	3.17 (dd, 11.6, 4.4)	90.6	3.18 (dd, 11.5, 4.6)	90.4
4		40.6		40.6		40.8		40.6		40.8		40.4		40.5
5	0.77 (brd, 11.5)	56.7	0.77 (brd, 11.6)	56.5	0.79 (brd, 11.6)	56.3	0.77 (brd, 11.2)	56.6	0.78 (brd, 11.6)	56.3	0.87 (dd, 12.4, 1.7)	56.6	0.77 (d, 11.4)	56.4
6	1.46	18.5	1.44	18.5	1.42	18.4	1.46	18.4	1.47	18.4	1.47	19.3	1.45	18.4
	1.60		1.61		1.63		1.60		1.63		1.65		1.60	
7	1.44	33.9	1.43	33.9	1.43	33.8	1.43	33.9	1.42	33.8	1.39	33.1	1.41	33.7
	1.68		1.70		1.72		1.69		1.72		1.39		1.70	
8		46.6		46.6		46.6		46.6		46.6		43.4		46.7
9	2.45 (s)	63.1	2.45 (s)	62.9	2.45 (s)	62.8	2.45 (s)	62.9	2.44 (s)	62.8	1.97 (s)	55.7	2.44 (s)	63.9
10		38.0		38.1		38.1		38.1		38.1		37.6		38.0
11		202.6		202.7		202.1		202.7		202.1	6.50 (dd, 10.6, 3.0)	128.1		202.7
12	5.56 (brs)	129.2	5.53 (brs)	128.9	5.65 (brs)	129.8	5.55 (brs)	128.9	5.55 (brs)	129.8	5.72 (dd, 10.7, 1.3)	129.0	5.69 (brs)	129.9
13		172.1		173.3		169.6		173.3		169.6		139.4		169.7
14		44.9		45.0		44.6		45.0		44.6		41.6		44.6
15	1.14	27.1	1.13	27.1	1.21	26.7	1.13	27	1.15	26.7	1.04	25.	1.21	26.6
	1.78		1.77		1.77		1.78	0.0	1.80		1.77	8	1.80	
16	1.48 (eq) 2.44 (dt, 14.3, 4.3) (ax)	25.1	1.43 (eq) 2.40 (dt, 14.3, 4.3) (ax)	26.2	1.50	26.0	1.46 (eq) 2.40 (dt, 14.2, 4.0) (ax)	26.2	1.50	26.0	1.48 (dt, 3.3, 13.7) (ax) 1.83 (dt, 13.7, 3.3) (eq)	29.5	1.51	25.7
					1.76				1.76				1.73	
17		38.1		38.5		44.4		38.5		44.4		45.5		44.5
18	2.20 brd (13.6)	44.6	2.31 (dd, 13.9, 2.9)	43.5	2.72 (dd, 13.7, 3.8)	42.7	2.31 (dd, 14.1, 3.4)	43.5	2.73 (dd, 13.4, 3.1)	42.7		132.4	2.78 (dd, 13.6, 4.0)	42.3
19	1.61 (eq) 2.17 t (13.6 Hz) (ax)	37.5	0.93 (eq) 2.22 (t, 13.0) (ax)	41.5	1.55 (dd, 14.3, 4.3) (eq) 2.29 (t, 13.9) (ax)	47.3	0.93 (eq) 2.22 (t, 13.3) (ax)	41.5	1.55 (dd, 13.9, 4.1) (eq) 2.30 (t, 13.7) (ax)	47.3	2.27 (d, 14.8) 2.80 (d, 14.6)	39.9	1.79 (eq) 2.12 (t, 14.1) (ax)	42.8
20		49.3		36.4		46.4		36.4		46.4		47.2		52.4
21	4.15 (t, 3.1)	70.3	3.47 (t, 3.9)	75.3		218.3	3.47 (t, 3.4)	75.4		218.3		218.6		217.2
22	1.66	39.4	1.52 (dd, 15.0, 3.6) 1.86 (dd, 15.0, 3.5)	38.3	2.03 (d, 14.5) 2.82 (d, 14.5)	46.8	1.52 (dd, 14.9, 3.5) 1.86 (dd, 14.8, 3.6)	38.3	2.03 (d, 14.5) 2.82 (d, 14.5)	46.8	2.19 (d, 14.4) 2.67 (d, 14.6)	48.5	2.10 (d, 14.4) 2.82 (d, 14.4)	47.6
	1.75													
23	1.05 (s)	28.3	1.05 (s)	28.4	1.06 (s)	28.5	1.06 (s)	28.4	1.05 (s)	28.5	1.04 (s)	28.2	1.05 (s)	28.4
24	0.85 (s)	16.9	0.85 (s)	17.0	0.87 (s)	16.9	0.84 (s)	17.0	0.85 (s)	16.9	0.83 (s)	16.5	0.84 (s)	17.0
25	1.13 (s)	16.9	1.13 (s)	17.0	1.14 (s)	17.0	1.14 (s)	17.0	1.13 (s)	17.0	0.93 (s)	18.6	1.12 (s)	17.0
26	1.11 (s)	19.3	1.11 (s)	19.2	1.12 (s)	19.2	1.11 (s)	19.2	1.12 (s)	19.2	0.77 (s)	17.2	1.11 (s)	19.2
27	1.41 (s)	23.1	1.43 (s)	23.2	1.40 (s)	23.4	1.43 (s)	23.1	1.40 (s)	23.4	1.02 (s)	21.0	1.40 (s)	23.7
28	3.30^c^	69	3.30^c^	69	3.36	67	3.30^c^	69	3.35 (d,	6	3.45 brs	67.	3.27	67.7
	3.01 (d, 11.2)	0.5	3.18 (d, 11.9)	0.5	(d, 10.9) 3.24 (m)	0.8	3.18 (d, 10.9)	0.5	11.1) 3.24 (d, 11.1)	7.8	3.45 brs	9	(d,11.7) 3.36 (d, 11.8) 3.90 (d, 11.8) 3.61 (d. 11.2)	
29	1.16 (s)	24.0	0.94 (s)	25.2	0.92 (s)	24.5	0.92 (s)	27.8	1.22 (s)	24.5	1.06 (s)	25.0	1.16 (s)	67.3
30		178.2	0.93 (s)	27.8	0.94 (s)	25.7	0.94 (s)	25.2	1.01 (s)	25.7	1.05 (s)	25.1	1.02 (s)	21.0
30-OMe	3.69 (s)	52.3												

a NMR spectra were measured on Bruker AV-III-600.

b Using CD_3_OD + D_2_O = 1:1 as solvent.

c The signals are overlapped with solvent peak.

**Table 2:** ^1^H and ^13^CNMR Data of Sugar Moieties of Compounds **1**–**7** (CD_3_OD)^a^

	**1**		**2**		**3**		**4**		**5**		**6**		**7** ^b^	
pos.	δ_H_ (*J* in Hz)	δ_C_	δ_H_ (*J* in Hz)	δ_C_	δ_H_ (*J* in Hz)	δ_C_	δ_H_ (*J* in Hz)	δ_C_	δ_H_ (*J* in Hz)	δ_C_	δ_H_ (*J* in Hz)	δ_C_		
Ara														
1′	4.32 (d, 7.7)	106.8	4.31 (d, 7.7)	106.8	4.31 (d, 5.2)	105.1	4.28 (d, 7.4)	107.0	4.31 (d, 7.8)	106.8	4.32 (d, 7.7)	106.8	4.32 (d, 7.9)	106.6
2′	3.73 (dd, 7.7, 9.0)	71.8	3.72 (m)	71.8	3.88 (m)	79.1	3.70 (dd, 9.7, 7.6)	72.1	3.71 (m)	71.8	3.73 (dd, 8.3, 7.8)	71.8	3.73 (m)	71.7
3′	3.55 (dd, 4.2, 9.0)	86.1	3.56 (t, 8.6)	86.1	3.88 (m)	73.2	3.62 (dd, 9.3, 3.2)	83.8	3.55 (t, 9.0)	85.8	3.55 (m)	86.1	3.58 (t, 9.0)	85.9
4′	4.01 (brs)	69.6	4.01 (brs)	69.3	3.89 (m)	68.7	4.01 brs	69.2	4.01 (brs)	69.5	4.02 (brs)	69.3	4.03 (brs)	69.4
5′	3,55 (brd, 11.8) 3.85 (dd, 11.8, 1.7)	67.1	3,56 (m) 3.85 (dd, 12.8, 1.8)	67.1	3,50 (dd, 12.8, 4.7) 3.83 (dd, 12.1, 2.3)	65.0	3.56 (brd 11.8) 3.82 (dd, 11.8, 2.1)	66.7	3.56 (m) 3.84 (dd, 12.8, 1.7)	67.1	3,56 (m) 3.85 (dd, 12.8, 1.9)	67.1	3,56 (m) 3.85 (dd, 11.5, 1.7)	67.1
Glc I														
1”	4.61 (d, 7.8)	104.6	4.604 (d, 7.7)	104.6	4.73 (d, 7.7)	103.2	4.54 (d, 7.7)	105.4	4.603 (d, 7.8)	104.6	4.608 (d, 7.9)	104.6	4.62 (d, 7.7)	104.5
2”	3.46 (dd, 9.4, 7.8)	85.3	3.46 (dd, 9.2, 8.2)	85.3	3.40 (dd, 9.4, 7.8)	84.7	3.28 (t, 8.2)	75.4	3.46 (dd, 8.8, 7.9)	85.3	3.46 (dd, 9.2, 7.7)	85.3	3.49 (dd, 8.8, 7.9)	84.9
3”	3.56 (t, 9.0)	77.6	3.56 (m)	77.5	3.56 (t, 8.9)	77.6	3.33 (t, 8.6)	77.7	3.56 (m)	77.5	3.55 (m)	77.6	3.58 (m)	77.4
4”	3.38 (t, 9.5)	70.4	3.40 (t, 9.1)	70.7	3.26 (m)	71.3	3.38 (t, 8.9)	71.3	3.38 (t, 8.6)	70.8	3.38 (t, 8.6)	70.7	3.40 (t, 9.2)	70.6
5”	3.29^c^	77.7	3.31^c^	77.6	3.26 (n)	78.0	3.31^c^	78.0	3.29^c^	77.7	3.30^c^	77.7	3.30^c^	77.5
6”	3.69 (dd, 11.8, 5.3) 3.81 (dd, 11.8, 2.3)	62.2	3.70 m 3.80 (dd, 11.9, 1.2)	62.1	3.61 (dd, 11.9, 5.9) 3.81 (dd, 11.3, 1.8)	63.0	3.70 (m) 3.80 (dd, 11.9, 1.2)	62.4	3.70 m 3.81 (dd, 12.2, 2.0)	62.2	3.70 (m) 3.81 (dd, 11.8, 2.3)	62.1	3.71 m 3.81 (dd, 11.9, 2.1)	62.0
Glc II														
1”’	4.599 (d, 7.8)	106.6	4.598 (d, 7.7)	106.6	4.57 (d, 7.8)	105.9			4.598 (d, 7.7)	106.6	4.601 (d, 7.7)	106.6	4.62 (d, 7.7)	106.3
2”’	3.25 (dd, 9.0, 8.3)	76.2	3.25 (t, 8.4)	76.1	3.26 (m)	76.3			3.25 (dd, 9.0, 7.9)	76.2	3.25 (dd, 8.7, 7.9)	76.2	3.25 (dd, 9.0, 7.9)	76.0
3′”	3.37 (t, 9.5)	77.7	3.38 (t, 8.3)	77.7	3.36 (m)	77.7			3.39 (m)	77.5	3.39 (t, 9.5)	77.7	3.40 (t, 9.2)	77.5
4’”	3.33 (m)	70.9	3.32 (m)	70.8	3.25 (m)	71.7			3.32 (m)	70.8	3.33 (m)	70.8	3.33 (m)	70.8
5′”	3.34 (m)	78.9	3.34 (m)	78.9	3.30 (m)	78.7			3.36 (m)	78.9	3.36 (m)	78.9	3.36 (m)	78.7
6”’	3.71 (dd, 12.0, 4.7) 3.90 (dd, 11.4, 1.9)	62.3	3.72 (m) 3.89 (dd, 12.5, 1.5)	62.2	3.69 (dd, 11.9, 4.9) 3.88 (m)	62.7			3.72 (m) 3.89 (dd, 12.7, 1.4)	62.2	3.70 (m) 3.90 (dd, 12.4, 1.6)	62.4	3.70 (m) 3.90 (brd, 11.9)	62.1

a NMR spectra were measured on Bruker AV-III-600.

b Using CD_3_OD + D_2_O = 1:1 as solvent.

c The signals are overlapped with solvent peak

#### Chenoponin B: 3β,21β,28-Trihydroxy-18β-olean-12-en-11-one

##### 3-*O*-[β-Sophorosyl-(1→3)]-α-l-arabinopyranoside (**2**)

IR (KBr): 3418, 2927, 2887, 2855, 1671 (sh), 1641 (sh), 1625, 1377, 1352, 1271, 1134, 1076, 1038, 1027, 922, 888, 886, 814, 726, 630, 617 cm^–1^; [α]_D_^25^ + 40.0 (*c* 0.1, MeOH); UV (MeOH) λ_max_ (log ε) 248.5 (4.00); CD (*c* 1.03 × 10^–5^ M, MeOH) [θ]_222.1_ 11864.6, [θ]_266.1_ −28662.6; ^1^H, ^13^C NMR data, see Tables 1 and 2; HMBC data, see Table S1; (−)-HRESIMS *m*/*z* 927.4989 [M–H]^−^ (calcd. for C_47_H_75_O_18_, 927.4959, Err = 3.2 ppm).

#### Chenoponin C: 3β,28-Dihydroxy-18β-olean-12-ene-11,21-dione

##### 3-*O*-[β-Sophorosyl-(1→2)]-α-l-arabinopyranoside (**3**)

IR (KBr): 3412, 2930, 2887, 2856, 1672 (sh), 1643, 1626, 1376, 1352, 1309, 1270, 1135, 1076, 1038, 1027, 922, 888, 856, 815, 726, 630, 617 cm^–1^; [α]_D_^25^ + 58.8 (*c* 0.1, MeOH); UV (MeOH) λ_max_ (log ε) 242.0 (4.08); CD (*c* 1.08 × 10^–5^ M, MeOH) [θ]_224.3_ 24370.8, [θ]_259.4_ −6267.2, [θ]_278.3_ 1067.9; ^1^H, ^13^C NMR data, see Tables 1 and 2; HMBC data, see Table S1; (−)-HRESIMS *m*/*z* 925.4795 [M–H]^−^ (calcd. for C_47_H_73_O_18_, 925.4802, Err = 0.7 ppm).

#### Chenoponin D: 3β,21β,28-Trihydroxy-18β-olean-12-en-11-one

##### 3-*O*-[β-Glucosyl-(1→3)]-α-l-arabinopyranoside (**4**)

IR (KBr): 3411, 2930, 2887, 2855, 1672 (sh), 1643, 1625, 1377, 1343, 1270, 1135, 1076, 1038, 1027, 922, 888, 856, 815, 726, 631, 617 cm^–1^; [α]_D_^25^ + 49.2 (*c* 0.1, MeOH); UV (MeOH) λ_max_ (log ε) 242.0 (3.96); CD (*c* 1.31 × 10^–5^ M, MeOH) [θ]_223.4_ 51940.3, [θ]_257.2_ −7589.5, [θ]_281.6_ 3307.3; ^1^H, ^13^C NMR data, see Tables 1 and 2; HMBC data, see Table S1; (−)-HRESIMS *m*/*z* 765.4409 [M–H]^−^ (calcd. for C_41_H_65_O_13_, 765.4431, Err = 2.2 ppm).

#### Chenoponin E: 3β,28-Dihydroxy-18β-olean-12-ene-11,21-dione

##### 3-*O*-[β-Sophorosyl-(1→3)]-α-l-arabinopyranoside (**5**)

IR (KBr): 3416, 2929, 2859, 1696 (sh), 1664, 1643, 1626, 1377, 1352, 1270, 1204, 1170, 1135, 1076, 1034, 1001, 922, 888, 856, 815, 727, 631, 618 cm^–1^; [α]_D_^25^ + 55.0 (*c* 0.1, MeOH); UV (MeOH) λ_max_ (log ε) 246.0 (4.05); CD (*c* 1.08 × 10^–5^ M, MeOH) [θ]_223.5_ 38146.8, [θ]_265.4_ −27945.1; ^1^H, ^13^C NMR data, see Tables 1 and 2; HMBC data, see Table S1; (−)-HRESIMS *m*/*z* 925.4835 [M–H]^−^ (calcd. for C_47_H_73_O_18_, 925.4802, Err = 3.5 ppm).

#### Chenoponin F: 3β,28-Dihydroxy-oleana-11,13(18)-dien-21-one

##### 3-*O*-[β-Sophorosyl-(1→3)]-α-l-arabinopyranoside (**6**)

IR (KBr): 3417, 2929, 2888, 2859, 1671 (sh), 1641 (sh), 1625, 1377, 1352, 1270, 1135, 1076, 922, 888, 856, 815, 727, 631, 617 cm^–1^; [α]_D_^25^ −1.90 (*c* 0.1, MeOH); UV (MeOH) λ_max_ (log ε) 242.5 (3.79); CD (*c* 1.10 × 10^–5^ M, MeOH) [θ]_223.1_ −95.8, [θ]_250.9_ −30614.1; ^1^H, ^13^C NMR data, see Table 1; HMBC data, see Table S1; (−)-HRESIMS *m*/*z* 909.4893 [M–H]^−^ (*calcd*. for C_47_H_73_O_17_, 909.4853, Err = 3.7 ppm).

#### Chenoponin G: 3β,28,30-Trihydroxy-18β-olean-12-ene-11,21-dione

##### 3-*O*-[β-Sophorosyl-(1→3)]-α-l-arabinopyranoside (**7**)

IR (KBr): 3420, 2925, 2888, 2856, 1650 (sh), 1626, 1374, 1339, 1270, 1134, 1075, 922, 888, 856, 814, 727, 630, 616 cm^–1^; [α]_D_^25^ + 45.2 (*c* 0.1, MeOH); UV (MeOH) λ_max_ (log ε) 242.5 (4.00); CD (*c* 1.06 × 10^–5^ M, MeOH) [θ]_223.1_ 11386.2, [θ]_265.7_ −26995.5; ^1^H, ^13^C NMR data, see Tables 1 and 2; HMBC data, see Table S1; (−)-HRESIMS *m*/*z* 941.4728 [M–H]^−^ (calcd. for C_47_H_73_O_19_, 941.4752, Err = 2.5 ppm).

### 2.4 Molecular Docking Studies

#### 2.4.1 Construction of the AG Template

A structural template model for human maltase-glucoamylase (MGAM) had been created previously by adapting PDB 2QLY, which shares 44% sequence identity with MGAM.^9^ The α-glucosidase (AG) template aligned well with MGAM’s active site, maintaining critical catalytic residues and structural elements typical of family 31 glycosyl hydrolases.

#### 2.4.2 Docking Simulation

The structures of ligands were depicted in ChemBioDraw, converted to favorable 3D structures using ChemBio3D, and optimized using the MMFF94x force field in MOE 2010.10. Docking was carried out with the triangle matcher method (RMSD < 0.6 Å) in a solvated active site. The docking scores were calculated using the London dG and Affinity dG scoring functions.^10,11^ The pose with the lowest score and no distortions was chosen for further analysis.

### 2.5 Inhibition Assay of α-Glucosidase

Compounds were subjected to an assay against α-glucosidase (type IV from *Bacillus stearothermophilus*, Sigma–Aldrich Co., Germany), according to a reported procedure^9^ with modifications. Briefly, to the sample (10 μL in the concentrations of 1000 μg/mL, 100 μg/mL or 10 μg/mL in 1% MeOH) in H_2_O (20 μL) was added α-glucosidase [3 U/mL in phosphate buffer (PBS), pH 6.5] and PBS (40 μL; pH 6.5). *p*-Nitrophenyl α-d-glucopyranoside (10 μL, 20 mM in PBS) was added after the mixture was incubated at 37 °C for 10 min. Then, the mixture was reacted for an additional 35 min. The sample was measured by the absorbance (A) at 405 nm by a microplate spectrophotometer (SPECTRAmax PLUS, Molecular Devices).

## 3 Results and Discussion

### 3.1 Isolation of Compounds **1**–**12** from *Chenopodium serotinum*

The methanol extract of the *C. serotinum* aerial part was divided into fractions soluble in CH_2_Cl_2_, EtOAc, *n*-BuOH, and H_2_O via a liquid–liquid partitioning process. As the *n*-BuOH-soluble fraction possessed saponin’s property, i.e., foam forming while its aqueous solution was stirred, this fraction was further investigated. Twelve compounds were isolated from this fraction via chromatography over Sephadex LH-20 and C-18 columns. They are eight triterpenoid glycosides (**1–8**), two ecdysteroids (**9**, **10**), and two norsesquiterpenoid glycosides (**11**, **12**) (Figure 1).

### 3.2 Structural Characterization

Compound **1** had a molecular formula of C_48_H_76_O_20_ as established by HR-ESI-MS, showing [M – H]^−^ at *m*/*z* 971.4857 (calcd. 971.4857). Subtracting the triterpene moiety (C_30_) and an ester methyl (C_1_), the latter being verified by one methyl singlet at δ_H_ 3.69 in its ^1^H NMR spectrum, from the molecular formula left a residue containing 17 carbons, corresponding to two hexosyl and one pentosyl moieties. The presence of three anomeric protons’ (δ_H_ 4.32, 4.61, 4.60) and carbons’ signals (δ_C_ 104.6, 106.6, 106.8) in its ^1^H and ^13^C NMR spectra (Table 1), respectively, verified this suggestion. The ^13^C NMR data of the glycon residue (Table 2) were almost identical to those reported data for the 3-*O*-triosyl residue of β-d-glucosyl*^p^*-(1→2)-β-d-glucosyl-(1→3)-α-l-arabinopyranosyl-phytolaccagenic acid 28-O-β-d-glucopyranoside,^8^ isolated from *C. quinoa*seeds, suggesting the same substitution at C-3 of **1** as this reported compound. This suggestion was supported by analysis of the COSY, HSQC, and HMBC spectra (Figures S6–S13, Supporting Information). The COSY spectrum showed key correlations δ_H_ 4.60 (Glc II H-1)/δ_H_ 3.25 (Glc II H-2), δ_H_ 4.61 (Glc I H-1)/δ_H_ 3.46 (Glc I H-2), δ_H_ 4.32 (Ara H-1)/δ_H_ 3.72 (Ara H-2)/δ_H_ 3.56 (Ara H-3)/δ_H_ 4.01 (Ara H-4)/δ_H_ 3.85 and 3.56 (Ara H_2_-5) (Figure 3), where the chemical shift for the corresponding proton attached carbons were assigned by analysis of the HSQC spectrum (Table 2; Figure S10, Supporting Information). The HMBC spectrum displayed the critical correlations δ_H_ 4.60 (Glc H-1″′)/δ_H_ 85.3 (Glc C-2″), δ_H_ 4.61 (Glc H-1″)/δ_H_ 86.1 (Ara C-3′), and δ_H_ 4.32 (Ara H-1′)/δ_H_ 90.4 (C-3) (Figure 3; Figures S11–S13, Supporting Information), thus establishing the linkage between each monosaccharide and between glycon and aglycon residues. The ^13^C NMR data of the aglycon residue of **1** (Table 1) was similar to that of glycyrrhizic acid methyl ester,^12^ except for one methylene and one methyine group (*C*H_2_OH, δ_C_ 69.5; *C*HOH, δ_C_ 70.3). The ^1^H NMR spectrum of **1** (Figure S1, Supporting Information) supported this suggestion by showing an AX system at δ_H_ 3.01 and 3.30 (*J* = 11.2 Hz) and the X part (δ_H X_ 4.15, t-like, *J* = 3.1 Hz) of an ABX system (δ_H A/B_ 1.66/1.77), verified by HSQC and COSY spectral analysis. Accordingly, this aglycon moiety was likely the dioxygenated derivative of glycyrrhetinic acid methyl ester. The HMBC spectrum (Figures S11–S13, Supporting Information) displayed the following critical correlations δ_H_ 1.16 (Me-29)/δ_C_ 178.2 (C, C-30), 49.3 (C, C-20), 70.3 (CH, C-21), 37.5 (CH_2_, C-19); δ_H_ 3.69 (MeO-30)/δ_C_ 178.2 (C, C-30); δ_H_ 3.01 and 3.30/δ_C_ 39.4 (CH_2_, C-22), and 25.1 (CH_2_, C-16) (Figure 2; Table S1, Supporting Information), designating the location of the hydroxylated position at C-21 and C-28 and the methyl ester at C-30. The NOESY spectrum (Figures S7–S9, Supporting Information) displayed the key correlations δ_H_ 1.16 (Me-29)/δ_H_ 4.15 (H_α_-21), 2.18 (H_ax_-19), and 1.63 (H_a_-22) (Figure 4), designating the signal of Me-29, the 21-hydroxy group being β-oriented, and the ring E being the boat form. The sequential NOESY correlations: δ_H_ 3.16 (H-3) to δ_H_ 1.05 (Me-23) & δ_H_ 0.85 (Me-24); δ_H_ 0.85 (Me-24) to δ_H_ 1.13 (Me-25) to δ_H_ 1.11 (Me-26) to δ_H_ 3.30 & δ_H_ 3.01 (H_2_-28); and δ_H_ 5.56 (H-12) to δ_H_ 2.19 (H-18), not only confirmed C-28 to be hydroxylated, but also designated the chemical shifts of the methyls and H-18 (Figure 4). Based on these analyses, compound **1** was elucidated as methyl 21β,28-dihydroxy-glycyrrhetinoate 3-*O*-[β-sophorosy-(1→3)]-α-L-arabinopyranoside (Figure 1). Elaborate analysis of ^1^H NMR, 2D NMR, and 1D selectively excited NMR spectra (1D TOCSY and 1D NOESY; Figure S3–5, 8 and S9, Supporting Information) led to the complete ^1^H and ^13^C NMR assignments of **1** as listed in Tables 1 and 2.

**Figure 2:**
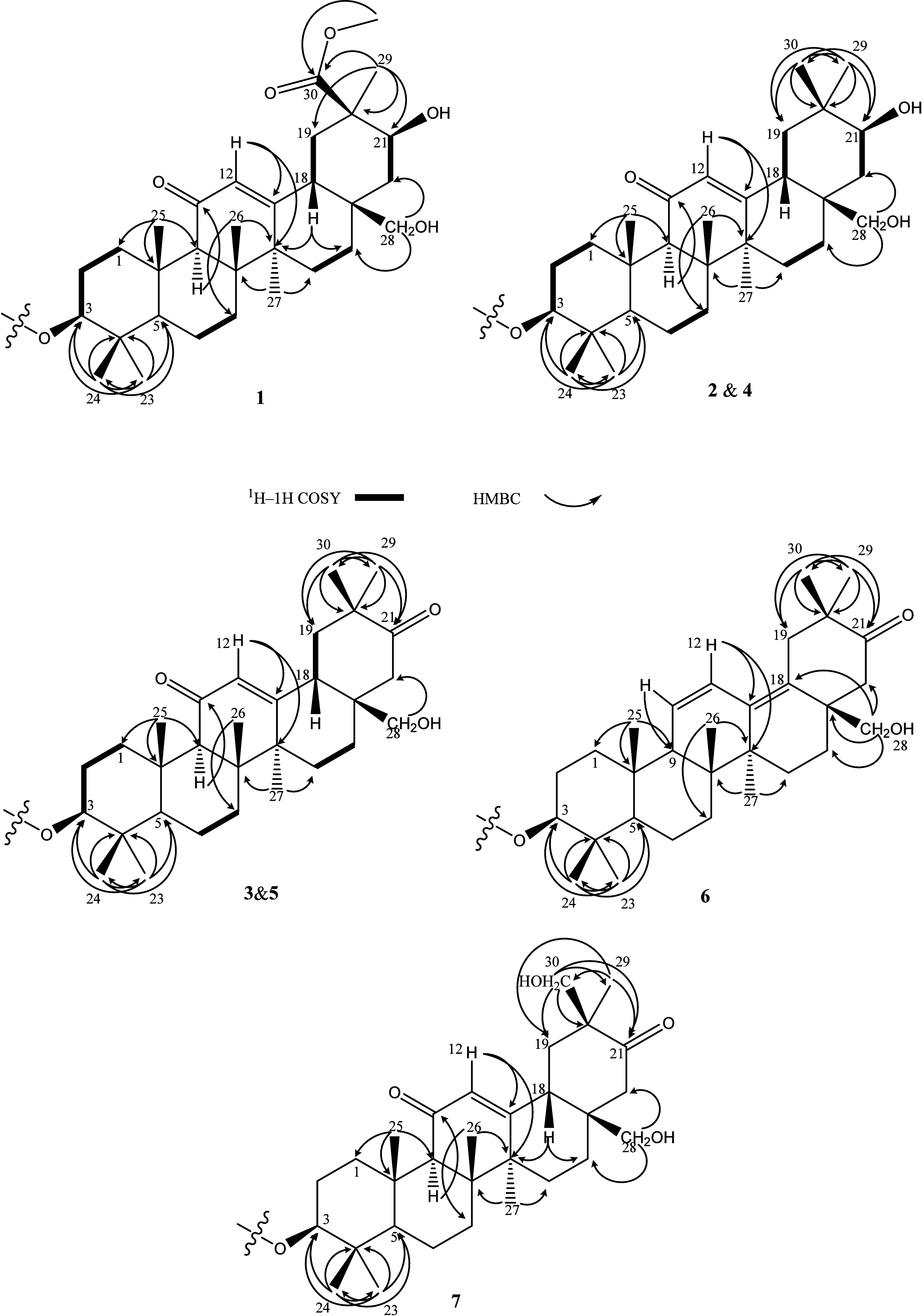
Key HMBC correlations of aglycon part of **1–7**.

**Figure 3:**
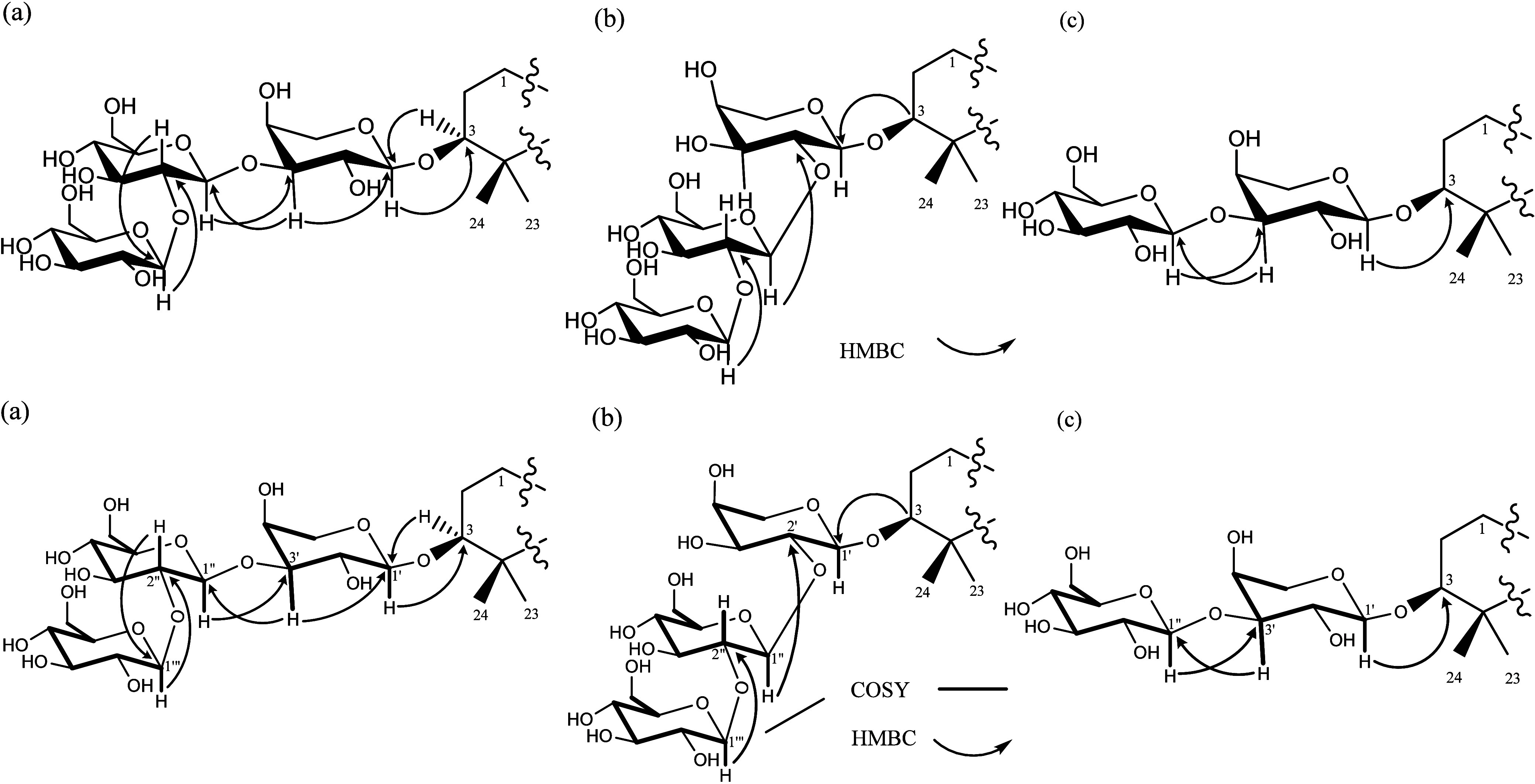
Key COSY and HMBC correlations of glycosidic linkages of **1**, **2**, and **5–7** (a); **3** (b) and **4** (c).

**Figure 4:**
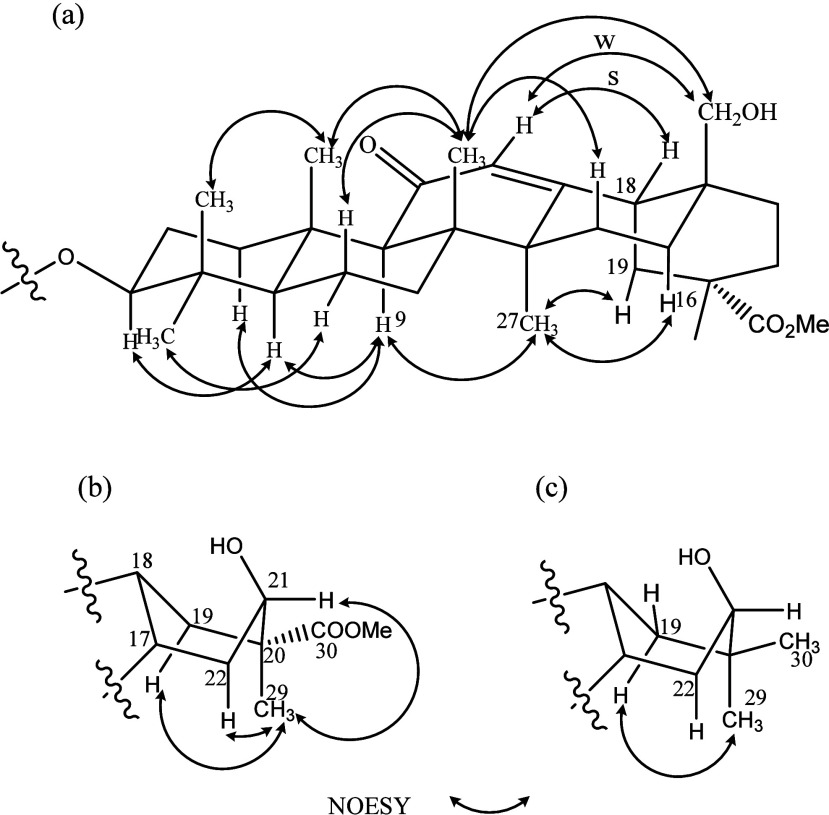
Key NOESY in rings A-D of **1** (a), ring E of **1** (b) and **2** (c).

Compound **2** had a molecular formula C_47_H_76_O_18_ as established by HR-ESI-MS, showing [M – H]^−^ at *m*/*z* 927.4869 (calcd. for C_47_H_75_O_18_ 927.4959), a CO_2_ residue less than that of **1**. Its ^1^H NMR spectrum (Figure S16, Supporting Information; Table 1) showing six methyl singlets was similar to that of **1** except for the ester methyl singlet in **1** (CO_2_ *Me*, δ_H_ 3.69) being replaced by a 4°-carbon linked methyl singlet, suggesting C-20 in **2** being dimethylated and consistent with the MS data indicated above. The ^13^C NMR spectrum of **2** (Figure S17, Supporting Information; Table 1) was almost superimposable to that of **1** except for the carbon signals in ring E. Accordingly, compound **2** was likely to be 3β,21β,28-trihydroxy-18β-olean-12-en-11-one 3-*O*-(β-sophorosyl-(1→3)-α-L-arabinopyranoside (Figure 1). This proposed structure for **2** was supported by analysis of 1D NOESY and HMBC spectra (Figures S18, S19, and S21; Table S1, Supporting Information). In the 1D NOESY experiment, selective irradiation at δ_H_ 0.94 (Me-29) enhanced δ_H_ 2.23 (H_ax_-19, t, *J* = 13.0 Hz) that, however, was not affected upon irradiating at δ_H_ 0.93 (Me-30) (Figure 4). The HMBC spectrum displayed the correlations δ_H_ 0.94 and 0.93/δ_C_ 75.3 (C-21), 41.5 (C-19), and 36.4 (C-20) in addition to mutual correlation to the attached carbons, i.e., δ_H_ 0.94 (Me-29)/δ_C_ 27.8 (C-30) and δ_H_ 0.93 (Me-30)/δ_C_ 25.2 (C-29) (Figure 2; Table S1, Supporting Information). These data confirmed the structure of **2** as suggested above.

Compound **4** had a molecular formula of C_41_H_66_O_13_ as established by HR-ESI-MS, showing [M – H]^−^ at *m*/*z* 765.4409 (calcd. for C_41_H_65_O_13_ 765.4431), short of a C_6_H_10_O_5_ residue relative to **2**, corresponding to a glucosyl moiety. The ^1^H and ^13^C NMR spectra of **4** were almost superimposable to those of **2** except for the absence of signals for a glucosyl residue (Tables 1 and 2; Figures S35 and S36, Supporting Information). Comparing to the corresponding signals in **2**, those of H-2″ and C-2″ in **4** were upfield shifted (H-2″: δ_H_ 3.28 vs δ_H_ 3.46; C-2″: δ_H_ 75.4 vs δ_H_ 85.3) (Table 2). The ^13^C NMR data of the glycon residue (Table 2) of **4** were almost identical to those reported data for the 3-*O*-diosyl residue, β-d-glucosyl*^p^*-(1→3)-α-L-arabinosyl*^p^*,^8^ suggesting the same substitution at C-3 of **4**. The presence of this glycon moiety was supported by the HMBC spectrum (Figures S39 and S40 , Supporting Information), displaying the correlations δ_H_ 4.599 (Glc 1″′)/δ_C_ 85.3 (Glc C-2″), δ_H_ 4.605 (Glc 1″)/δ_C_ 86.1 (Ara C-3′), δ_H_ 4.32 (Ara H-1′)/δ_C_ 90.4 (C-3) (Figure 3; Table S1, Supporting Information), and the COSY spectrum (Figure S38, Supporting Information), displaying the sequential correlations with each other: δ_H_ 4.54 (Glc 1″) to δ_H_ 3.28 (Glc H-2″), δ_H_ 4.28 (Ara H-1′) to δ_H_ 3.70 (Ara H-2′) and to δ_H_ 3.62 (Ara H-3′), and δ_H_ 3.16 (H-3) to δ_H_ 1.15 and 1.77 (H_2_-2). [25]) (Figure 3). These data thus established **4** as 3β,21β,28-trihydroxy-18β-olean-12-en-11-one 3-*O*-[β-d-glucosyl-(1→3)]-l-arabinopyranoside (Figure 1).

Compounds **3** and **5** had the same molecular formula of C_47_H_74_O_18_ as supported by HR-ESI-MS, showing [M – H]^−^ at *m*/*z* 925.4795 for **3** (*calcd*. for C_47_H_73_O_18_, 925.4802) and 925.4835 for **5** (*calcd*. for C_47_H_73_O_18_, 925.4802), 2H less than that of **2**. The ^1^H and ^13^C NMR spectral analyses indicated that **3** and **5** had the same aglycon residue, which is only slightly different from those of **2** and **4** whose carbinoyl C-21 was replaced by a ketone group (**2**: δ_H‑21_ 4.15 & δ_C‑21_ 70.3; **3**: δ_C‑21_ 218.3) (Tables 1 and 2), consistent with their MS difference. HMBC analysis supported this suggestion by showing key correlations δ_H‑29_ 0.92 & δ_H‑30_ 0.94/δ_C‑21_ 218.3, δ_C‑20_ 46.4, and δ_C‑19_ 47.3 (Figure 2; Table S1, Figures S30 and S46, Supporting Information). Thus, the aglycon part of **3** and **5** was 3β,28-dihydroxy-18β-olean-12-ene-11,21-dione. Analysis of the 1D-TOCSY spectra (Figures S26 and S27, Supporting Information) also indicated the glycon of **3** being composed of two glucosyl and one arabinosyl residues. The glycosidic linkage in **3** [Glc-(1→2)-Glc(1→2)-Ara–O-C-3] was elucidated by the HMBC spectrum showing correlations δ_H_ 3.18 (H-3)/ δ_C_ 105.1 (C-1′), δ_H_ 4.73 (H-1″)/ δ_C_ 79.1 (C-2′), and δ_H_ 4.57 (H-1′″)/ δ_C_ 84.7 (C-2″) (Figure 3; Figures S31 and S32, Supporting Information). The ^1^H and ^13^C NMR spectra of the glycon part of **5** (Figures S43 and S44, Supporting Information; Table 2) were superimposable to those of **1**, indicating both bearing the same glycon, which was also confirmed by analysis of the HMBC spectrum (Figure S46, Supporting Information; Table S1). Therefore, the structures of **3** and **5** were elucidated as 3β,21β,28-trihydroxy-18β-olean-12-en-11-one 3-*O*-[β-sophorosyl-(1→2)]-α-l-arabinopyranoside and 3β,21β,28-trihydroxy-18β-olean-12-en-11-one 3-*O*-[β-sophorosyl-(1→3)]-α-l-arabinopyranoside, respectively (Figure 1).

Compounds **1**–**5** and **7** possessed 11-*oxo*-olean-12-enol derivatives as their aglycon part, verified by their ^1^H and ^13^C NMR spectra, displaying six (**1**, **7**) to seven (**2**–**5**) methyl singlets in the aliphatic region, and characteristic signals for the β-disubstituted α,β-unsaturated ketone moiety at δ_H_ 5.50 (H-12, brs; **1**) and δ_C_ 202.6 (C, C-11; **1**), 129.6 (CH, C-12; **1**), and 172.1 (C, C-13; **1**), and UV absorption around 248 nm like that of glycyrrhizic acid and its aglycon.^12,13^

Compound **6** was elucidated as 3β,28-dihydroxy-olean-11,13(18)-diene-21-one 3-*O*-[3-*O*-(2-*O*-β-d-glucopyranosyl)- β-d-glucopyranosyl]-α-l-arabinopyranoside. As deduced from HR-ESI-MS showing [M – H]^−^ at *m*/*z* 909.4893 (*calcd*. for C_47_H_73_O_17_, 909.4853), it had a molecular formula of C_47_H_74_O_17_, an oxygen atom less than that of **5**. Compound **6** had an identical glycon moiety to **5**, as supported by the ^13^C NMR spectral data (Table 2; Figure S50, Supporting Information). The aglycon moiety of **6** is similar to that of **5** except for the replacement of the chromophore α,β-unsaturated ketone with a conjugated diene. This difference was verified by the ^1^H and ^13^C NMR spectra of **6** by showing the *cis*-coupled H-11 (δ_H_ 6.50) and H-12 (δ_H_ 5.72) (*J* = 10.6 Hz) and four olefinic carbon signals (δ_C‑11_ 128.1, δ_C‑12_ 129.0, δ_C‑13_ 139.4, and δ_C‑18_ 132.4).^14^ This structural moiety in the aglycon was supported by the HMBC spectrum (Figure S52, Supporting Information), showing the characteristic correlations δ_H_ 6.50 (H-11)/δ_C_ 55.7 (C-9), δ_H_ 5,72 (H-12)/δ_C_ 139.4 (C-13) and 41.6 (C-14) (Figure 2; Table S1, Supporting Information).

Compound **7** had a molecular formula of C_47_H_74_O_19_ as supported by HR-ESI-MS, showing [M – H]^−^ at *m*/*z* 941.4728 (calcd. for C_47_H_73_O_19_, 941.4752), an oxygen atom more than **5**. Its ^1^H NMR spectrum (Figure S55, Supporting Information) was similar to that of **5** except for one methyl singlet being replaced by an AX system (δ_H_ 3.61, 3.90, *J* = 11.5 Hz) (Table 1). The MS and ^1^H NMR data thus indicated one methyl group in **5** to be hydroxylated in **7**. This was verified by the ^13^C NMR spectrum (Figure S56, Supporting Information) displaying an extra oxymethylene signal at δ_C_ 67.3 relative to that of **5** (Table 1) and was β-substituted at the C-20 position, designated by analysis of the HMBC spectrum (Figure S58, Supporting Information), showing the key correlation δ_H_ 1.02 (Me-29)/δ_C_ 67.3 (C-30) and 217.2 (C-21) (Figure 2; Table S1, Supporting Information). Accordingly, **7** was elucidated as 3β,28,30-trihydroxy-12-oleanene-11,21-dione-3-*O*-[β-sophorosyl-(1→3)]-α-l-arabinopyranoside (Figure 1). The same as compounds **1**–**5**, compound **7** also possessed a 11-*oxo*-olean-12-enol aglycon, six methyl singlets in the aliphatic region, and a β-disubstituted α,β-unsaturated ketone moiety.

Among these, compound **8** was elucidated as oleanolic acid 3,28-di-*O*-glucoside, i.e., Chikusetsu Saponin IVa, by comparison of its physical data (Table S2, Supporting Information) to those reported.^15^

Compounds **9** and **10** were identified as β-ecdysone and makisterone A (24-methyl-β-ecdysone),^16–18^ respectively. The ^1^H NMR spectrum of the latter displayed an additional methyl signal at δ_H_ 0.92 (d, *J* = 6.8 Hz) relative to that of the former (Table S3, Supporting Information). Both of them possess a characteristic UV absorption maximum at 242 nm for the enone chromophore. Compound **11** was identified as stophylionoside D,^19^ while **12** was its C-8 epimer, icariside B_1_ ^19,20^ (austroside B). Their ^1^H NMR data (Table S4, Supporting Information) indicated characteristic signals for the allenyl conjugated carbonyl residue, δ_H‑8_ 5.82 (**11**) and 5.90 (**12**).^19^ In addition, the ^13^C NMR spectrum of **11** showed distinct signals for this residue at δ_C_ 120.1 (C-6), 200.8 (C-7), 101.2 (C-8), and 211.4 (C-9)^19^ (Table S4; Supporting Information).

### 3.3 Molecular Docking of **1**–**12** toward Human α-Glucosidase

Table 3 displays the docking results for chenoponins A–G (**1**–**7**), oleanolic acid 3,28-di-*O*-glucoside (**8**), β-ecdysterone (**9**), makisterone A (**10**), stophylionoside D (**11**), and icariside B_1_ (**12**) toward human α-glucosidase (AG).

**Table 3:** H-Bond Interaction of Isolated Compounds **1**–**12** with α-Glucosidase^a^

compd.	Δ_H_ *G* kcal/mol	IC_50_ (μM)	His674	Asp645	Asp616	Asp518	Asp443	Asp404	Asp282	Tr481	Trp376	glycon conformation^b^
**1**	–18.43	23.25 ± 1.00	3‴–OH			2‴–OH		3″–OH	4′–OH		4″–OH	C B B
**2**	–18.01	55.25 ± 2.14	3‴–OH		4‴–OH	2‴–OH	6″–OH			O″		C B B
**3**	–15.31		6‴–OH		2‴–OH	3‴–OH	4″–OH	3′–OH				C B B
**4**	–17.21		2″–OH		6″–OH						2′–OH	C B
**5**	–18.30		3‴–OH		6‴–OH	2‴–OH	6″–OH		4′–OH	O″		C B B
**6**	–18.42		3″–OH	6″–OH			6‴–OH	2‴–OH				C B B
								3‴–OH				
**7**	–17.90	66.21 ± 0.37	3‴–OH	6‴–OH	4‴–OH		6″–OH	2‴–OH		O″		C B C
								4″–OH				
**8**	–15.68		4″–OH	6″–OH								
**9**	–15.38		22-OH		25-OH			20-OH				
**10**	–15.75		22-OH			25-OH		20-OH				
**11**	–13.36	>249.87	6′–OH		4′–OH	3′–OH						
**12**	–13.30				2′–OH	3′–OH		6′–OH				

a 2″-O of chenoponin D (**4**) formed ionic bond with Arg600 *N*η1.

b C: chair; B: boat; starting from 3-O-linked monosaccharide.

The docking result revealed a consistent migration pattern from the glycon units of **1**–**7** to the binding pocket of AG and orientation of the aglycon units toward the hydrophobic region. The hydroxyl groups in the glycon residue frequently formed hydrogen bonds (H-bonds) with His674, Asp616, Asp518, Asp404, Asp282, and Tyr376 in the AG binding site, similar to those observed between the tetraose acarbose and AG.

Relative to **2**, the 20β-CO_2_Me group in **1** formed an intramolecular H-bond with 21-OH, leading to the reduction of structural flexibility and entropy (Figure S61, **1** vs **2**), while the sp^2^-hybridized C-21 in **5** reduced steric clash near the binding site entrance. Hence, **1** and **5** had better binding affinity than **2** [Δ*G* = −18.93 (**1**), −18.30 (**5**), and −18.09 kcal/mol (**2**)]. As **4** lacks the terminal glucosyl unit of **2**, it formed fewer H-bonds, resulting in a weaker binding affinity (Δ*G* = −17.21 kcal/mol).

Relative to **5**, the additional 30-OH group in **7** encountered steric hindrance during docking (Figure S62, **5** vs **7**), whereas the conjugated diene in **6** showed a negligible effect on its docking conformation. Consequently, **7** had a weaker binding affinity (Δ*G* = −17.90 kcal/mol) while **6** showed comparable binding affinity to **5** (Δ*G* = −18.42 vs −18.30 kcal/mol, **5**).

Compound **3** adopted an arc-bent conformation ascribable to the sugar linkage during docking, whereas **5** remained extended (Figure 5, **3** vs **5**). This bending obstructed the glycon moiety in **3** from entering the AG binding pocket, leading to a lower binding affinity (Δ*G* = −15.64 kcal/mol).

**Figure 5:**
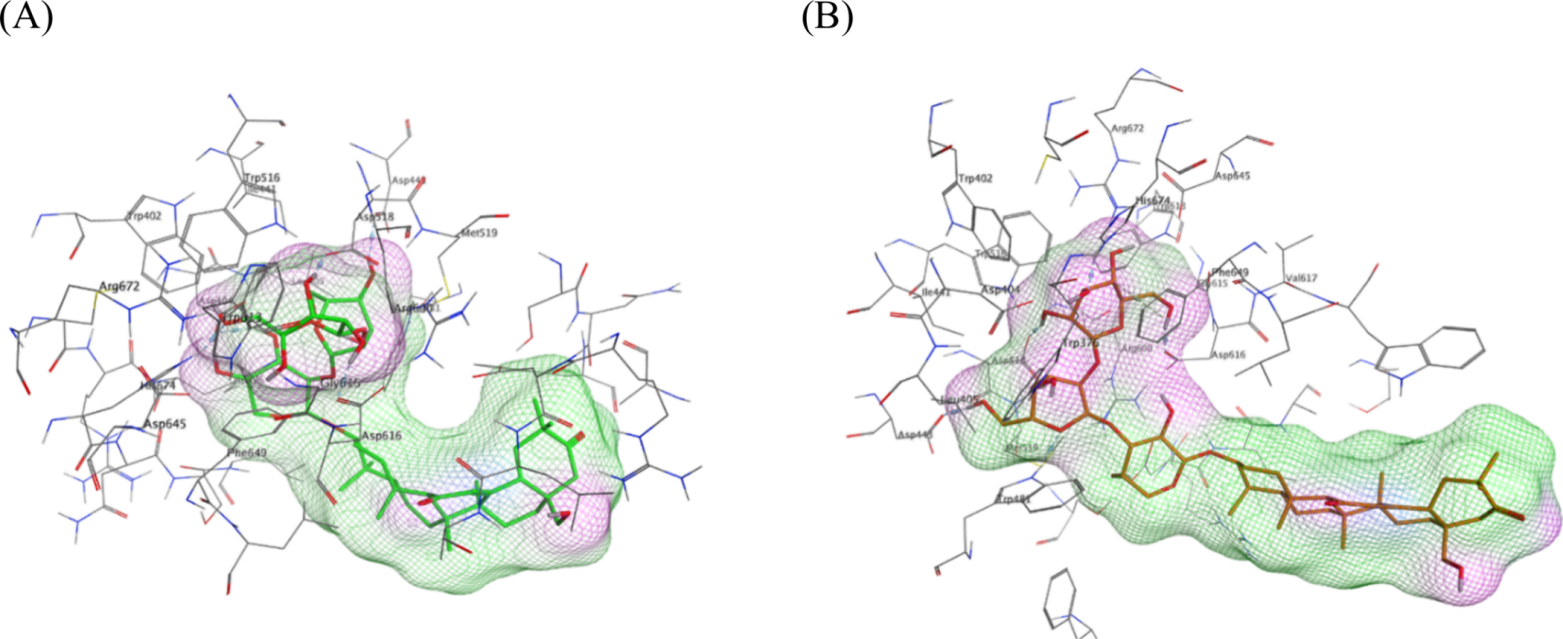
Surface depiction of **3** (A) and **5** (B) docked with the AG.

The bisdesmoside **8** containing 3-*O*-Glc and 28-*O*-Glc showed limited interaction with AG, yielding a weaker binding affinity (Δ*G* = −15.64 kcal/mol). Steroidal compounds **9** and **10** displayed similar binding affinities (Δ*G* = −15.38 vs −15.75 kcal/mol, **10**), indicating that the 24-methyl group in **10** had little effect while interacting with AG. The geometric isomers **11** and **12** exhibited weak binding affinity (Δ*G* = −13.36 vs −13.30 kcal/mol, **12**).

### 3.4 In Vitro Assay of **1**, **2**, **7**, and **11** against α-Glucosidase

Bioassay of the relatively major isolates (**1**, **2**, **7**, and **11**) against α-glucosidase was performed. The result (Table 3) showed their IC_50_ values to be 23.25, 55.25, 66.21, and >249 μM, respectively, where the IC_50_ value of acarbose, the positive control, was 0.03 μM. The order of their anti-α-glucosidase activity was consistent with the binding affinity from in silico virtual screening as discussed above.

Seven new glycosides were isolated and characterized structurally from *Chenopodium serotinum*. Six of them (**1**–**5** and **7**) possessed an olean-12-en-11-one residue, and the remaining one possessed an oleana-11,13(18)-diene residue as the aglycon. The former skeleton was commonly present in licorice,^21^ such as to glycyrrhetinic acid, but different from those reported from *C. quinoa* seeds.^8^ The glycon part of the triosides, β-D-Glc*^p^*-(1→2)-β-D-Glc*^p^*- (1→3)-L-Ara*^p^* and β-D-Glc*^p^*-(1→2)-β-D-Glc*^p^*- (1→2)-L-Ara*^p^*, however, was the same as those from *C. quinoa*.^22^ As glycyrrhetinic acid displayed anti-inflammatory activity, the isolates **1–5** and **7** were bioassayed against COX-1 and COX-2; however, they were found inactive at 30 μM. *In silico* virtual screening on α-glucosidase followed by an *in vitro* assay did indicate **1** to possess moderate inhibition toward this enzyme, which is beneficial to diabetic patients and adds value to this everlasting wild vegetable.

## Supplementary Material



## Notes

Spectra (1D and 2D NMR, IR, UV, CD and MS) for **1**–**7** and spectroscopic data for **8**–**12** (PDF)

## References

[1] Liu, Ho-Yih “20. Chenopodiaceae” in Flora of Taiwan, 2 ^nd^ ed. Huang; Tseng-Chieng , Ed.; Ed.ial Committee of the Flora of Taiwan: Taipei, 1996, Vol. 2, pp 385–386.

[2] Ren Y., He P., Xu J., Jia J. (2017). Distribution pattern of riparian
invasive plants in Luanhe Basin, North China and its relationship
with environment. J. Appl. Ecol..

[3] Song K., Wang H.-Q., Liu C., Kang J., Li B.-M., Chen R.-T. (2014). Chemical constituents
from *Chenopodium ambrosioides*. China J. Chin. Mater. Med..

[4] Tsai P. J., Chen Y. S., Sheu C. H., Chen C. Y. (2011). Effect of nanogrinding
on the pigment and bioactivity of Djulis (*Chenopodium formosanum* Koidz.). J. Agric. Food. Chem..

[5] Poonia A., Upadhayay A. (2015). *Chenopodium
album* Linn: review of
nutritive value and biological properties. J.
Food Sci. Technol..

[6] Lin M., Han P., Li Y., Wang W., Lai D., Zhou L. (2019). Quinoa secondary
metabolites and their biological activities or functions. Molecules.

[7] Usman L. A., Hamid A. A., Muhammad N. O., Olawore N. O., Edewor T. I., Saliu B. K. (2010). Chemical constituents
and anti-inflammatory activity
of leaf essential oil of Nigerian grown *Chenopodium album* L. EXCLI J..

[8] Dini I., Schettino O., Simioli T., Dini A. (2001). Studies on the constituents
of *Chenopodium quinoa* seeds: Isolation and characterization
of new triterpene saponins. J. Agric. Food Chem..

[9] Sim L., Jayakanthan K., Mohan S., Nasi R., Johnston B. D., Pinto B. M., Rose D. R. (2010). New glucosidase inhibitors from an
Ayurvedic herbal treatment for type 2 diabetes: structures and inhibition
of human intestinal maltase-glucoamylase with compounds from *Salacia reticulata*. Biochemistry.

[10] Labute P. (2009). Protonate3D:
assignment of ionization states and hydrogen coordinates to macromolecular
structures. Proteins.

[11] Halgren T.
A. (1996). Merck molecular force
field. I. Basis,
form, scope, parameterization, and performance of MMFF94. J. Comput. Chem..

[12] Baltina L. A., Kunert O., Fatykhov A. A., Kondratenko R. M., Spirikhin L. V., Baltina L. A., Galin F. Z., Tolstikov G. A., Haslinger E. (2005). High-resolution ^1^H and ^13^C NMR of glycyrrhizic acid and its esters. Chem. Nat. Compd..

[13] Patil S. K., Salunkhe V. R., Mohite S. K. (2012). Development and
validation of UV
spectrophotometric method for estimation of glycyrrhetinic acid in
hydroalcoholic extrat of *Glycyrrhiza glabra*. Int. J. Pharm., Chem. Biol. Sci..

[14] Yamamoto A., Miyase T., Ueno A., Maeda T. (1993). Scrophulasaponins
II-IV,
new saikosaponin homologs from *Scrophularia kakudensis* Franch. Chem. Pharm. Bull..

[15] Kunert O., Haslinger E., Schmid M. G., Reiner J., Bucar F., Mulatu E., Abebe D., Debella A. (2000). Three saponins, a steroid,
and a flavanol glycoside from *Achyrantes aspera*. Chem. Mon..

[16] Girault J. P., Lafont R. (1988). The complete ^1^H-NMR assignment of ecdysone
and 20-hydroxyecdysone. J. Insect Physiol..

[17] Zhu N., Kikuzaki H., Vastano B. C., Nakatani N., Karwe M. V., Rosen R. T., HO C. T. (2001). Ecdysteroids
of Quinoa Seeds (*Chenopodium quinoa* Willd.). J. Agric.
Food Chem..

[18] Canonica L., Danieli B., Ferrari G., Krepinsky J., Weisz-Vincze I. (1975). A novel method of isolation of phytoecdysones
from
Kaladana seeds. Phytochemistry.

[19] Yu Q., Matsunami K., Otsuka H., Takeda Y. (2005). Staphylionosides A–K:
Megastigmane glucosides from the leaves of *Staphylea bumalda* DC. Chem. Pharm. Bull..

[20] Wang J., Shen Y., He H., Kang W., Hao X. (2005). Norsesquiterpenoid
and sesquiterpenoid glycosides from *Evodia austrosinensis*. Planta Med..

[21] Schmid C., Dawid C., Peters V., Hofmann T. (2018). Saponins from European
Licorice roots (*Glycyrrhiza glabra*). J. Nat. Prod..

[22] Dini I., Tenore G. C., Schettino O., Dini A. (2001). New oleanane saponins
in *Chenopodium quinoa*. J. Agric.
Food Chem..

