# Supplementary material for: Oleanane-Type Glycosides with α‑Glucosidase Inhibitory Activity from Chenopodium serotinum L., an Everlasting Wild Vegetable
Source: J Agric Food Chem. 2025 Nov 17;73(47):30369–79. doi: 10.1021/acs.jafc.5c12681 (PMC12670393; doi:10.1021/acs.jafc.5c12681)
Supplement: Supplementary file 1 [file jf5c12681_si_001.pdf]

Supplementary Information

Oleanane-type glycosides with α-glucosidase inhibitory activity from *Chenopodium serotinum* L., an everlasting wild vegetable

Chien-Yi Chen [a], Sheau Ling Ho [b], Sheng-Fa Tsai [a], Ju-Fang Liu [c], Shoei-Sheng Lee [a,], Chia-Chuan Chang [a,*]*

[a] *School of Pharmacy, College of Medicine, National Taiwan University, Taipei 10050, Taiwan, Republic of China*

[b] *Department of Chemical & Materials Engineering, Chinese Culture University, Taipei 11114, Taiwan, Republic of China*

[c] *School of Oral Hygiene, College of Oral Medicine, Taipei Medical University, Taipei 11031, Taiwan, Republic of China*

[*] Corresponding author. Tel./fax: +886 2 23916127. *E-mail address:* shoeilee@ntu.edu.tw (SSL)
Tel./fax: +886 2 23919098. *E-mail address:* chiachang@ntu.edu.tw (CCC)

Page	Content
5	Spectroscopic data of **8**－**12**
6	**Table S1**. HMBC spectroscopic data of aglycon part of compounds **1-7** and NOESY data of **1**
7	**Table S2**. ^{1}H and ^{13}C spectroscopic data of compound **8**
8	**Table S3**. ^{1}H and ^{13}C spectroscopic data of compounds **9** and **10**
9	**Table S4**. ^{1}H and ^{13}C spectroscopic data of compounds **11** and **12**
10	**Fig. S1**. ^{1}H NMR spectrum of **1** (CD_3OD, 600 MHz)
11	**Fig. S2**. ^{13}C NMR spectrum of **1** (CD_3OD, 150 MHz)
12	**Fig. S3**. 1D-TOCSY spectrum of **1** (CD_3OD, 600 MHz) (H-1′ &5′ b)
13	**Fig. S4**. 1D-TOCSY spectrum of **1** (CD_3OD, 600 MHz) (H-2″ &6″ b)
14	**Fig. S5**. 1D-TOCSY spectrum of **1** (CD_3OD, 600 MHz) (H-6‴ b)
15	**Fig. S6**. COSY spectrum of **1** (CD_3OD, 600 MHz)
16	**Fig. S7**. NOESY spectrum of **1** (CD_3OD, 600 MHz)
17	**Fig. S8**. 1D-NOESY spectrum of **1** (CD_3OD, 600 MHz) (Me-26 & Me-29)
18	**Fig. S9**. 1D-NOESY spectrum of **1** (CD_3OD, 600 MHz) (Me-27)
19	**Fig. S10**. HSQC spectrum of **1** (CD_3OD, 600 MHz)
20	**Fig. S11**. HMBC spectrum of **1** (CD_3OD, 600 MHz)
21	**Fig. S12**. HMBC spectrum of **1** (CD_3OD, 600 MHz) (glycosidic linkages)
22	**Fig. S13**. HMBC spectrum of **1** (CD_3OD, 600 MHz) (aliphatic region)
23	**Fig. S14**. IR spectrum of **1**
24	**Fig. S15**. UV spectrum of **1**
25	**Fig. S16**. CD spectrum of **1**
26	**Fig. S17**. MS spectrum of **1** (positive mode)
27	**Fig. S18**. MS spectrum of **1** (negative mode)
28	**Fig. S19**. ^{1}H NMR spectrum of **2** (CD_3OD, 600 MHz)
29	**Fig. S20**. ^{13}C NMR spectrum of **2** (CD_3OD, 150 MHz)
30	**Fig. S21**. 1D-NOESY spectrum of **2** (CD_3OD, 600 MHz) (Me-27 & H-12)

31	**Fig. S22**. 1D-NOESY spectrum of **2** (CD_3OD, 600 MHz) (Me-29 & Me-30)
32	**Fig. S23**. HSQC spectrum of **2** (CD_3OD, 600 MHz)
33	**Fig. S24**. HMBC spectrum of **2** (CD_3OD, 600 MHz)
34	**Fig. S25**. IR spectrum of **2**
35	**Fig. S26**. UV spectrum of **2**
36	**Fig. S27**. CD spectrum of **2**
37	**Fig. S28**. MS spectrum of **2** (positive mode)
38	**Fig. S29**. MS spectrum of **2** (negative mode)
39	**Fig. S30**. ^{1}H NMR spectrum of **3** (CD_3OD, 600 MH)
40	**Fig. S31**. ^{13}C NMR spectrum of **3** (CD_3OD, 150 MHz)
41	**Fig. S32**. 1D-TOCSY spectrum of **3** (CD_3OD, 600 MHz) (H-1′)
42	**Fig. S33**. 1D-TOCSY spectrum of **3** (CD_3OD, 600 MHz) (H-1″ &1‴)
43	**Fig. S34**. COSY spectrum of **3** (CD_3OD, 600 MHz)
44	**Fig. S35**. HSQC spectrum of **3** (CD_3OD, 600 MHz)
45	**Fig. S36**. HMBC spectrum of **3** (CD_3OD, 600 MHz)
46	**Fig. S37**. HMBC spectrum of **3** (CD_3OD, 600 MHz) (glycosidic linkages-1)
47	**Fig. S38**. HMBC spectrum of **3** (CD_3OD, 600 MHz) (glycosidic linkages-2)
48	**Fig. S39**. HMBC spectrum of **3** (CD_3OD, 600 MHz) (Proof of the ketone at C-21)
49	**Fig. S40**. IR spectrum of **3**
50	**Fig. S41**. UV spectrum of **3**
51	**Fig. S42**. CD spectrum of **3**
52	**Fig. S43**. MS spectrum of **3** (positive mode)
53	**Fig. S44**. MS spectrum of **3** (negative mode)
54	**Fig. S45**. ^{1}H NMR spectrum of **4** (CD_3OD, 600 MH)
55	**Fig. S46**. ^{13}C NMR spectrum of **4** (CD_3OD, 150 MHz)
56	**Fig. S47**. 1D-TOCSY spectrum of **4** (CD_3OD, 600 MHz) (H-1′ &1″)
57	**Fig. S48**. COSY spectrum of **4** (CD_3OD, 600 MHz)
58	**Fig. S49**. HMBC spectrum of **4** (CD_3OD, 600 MHz)
59	**Fig. S50**. HMBC spectrum of **4** (CD_3OD, 600 MHz) (glycosidic

	linkages)
60	**Fig. S51**. IR spectrum of **4**
61	**Fig. S52**. UV spectrum of **4**
62	**Fig. S53**. CD spectrum of **4**
63	**Fig. S54**. MS spectrum of **4** (positive mode)
64	**Fig. S55**. MS spectrum of **4** (negative mode)
65	**Fig. S56**. ^{1}H NMR spectrum of **5** (CD_3OD, 600 MHz)
66	**Fig. S57**. ^{13}C NMR spectrum of **5** (CD_3OD, 150 MHz)
67	**Fig. S58**. HSQC spectrum of **5** (CD_3OD, 600 MHz)
68	**Fig. S59**. HMBC spectrum of **5** (CD_3OD, 600 MHz)
69	**Fig. S60**. HMBC spectrum of **5** (CD_3OD, 600 MHz) (Proof of the ketone at C-21)
70	**Fig. S61**. IR spectrum of **5**
71	**Fig. S62**. UV spectrum of **5**
72	**Fig. S63**. CD spectrum of **5**
73	**Fig. S64**. ^{1}H NMR spectrum of **6** (CD_3OD, 600 MH)
74	**Fig. S65**. ^{13}C NMR spectrum of **6** (CD_3OD, 150 MHz)
75	**Fig. S66**. HSQC spectrum of **6** (CD_3OD, 600 MHz)
76	**Fig. S67**. HMBC spectrum of **6** (CD_3OD, 600 MHz)
77	**Fig. S68**. IR spectrum of **6**
78	**Fig. S69**. UV spectrum of **6**
79	**Fig. S70**. CD spectrum of **6**
80	**Fig. S71**. ^{1}H NMR spectrum of **7** (CD_3OD+D_2O, 600 MH)
81	**Fig. S72**. ^{13}C NMR spectrum of **7** (CD_3OD+D_2O, 150 MHz)
82	**Fig. S73**. HSQC spectrum of **7** (CD_3OD+D_2O, 600 MHz)
83	**Fig. S74**. HMBC spectrum of **7** (CD_3OD+D_2O, 600 MHz)
84	**Fig. S75**. IR spectrum of **7**
85	**Fig. S76**. UV spectrum of **7**
86	**Fig. S77**. CD spectrum of **7**
87	**Fig. S78**. MS spectrum of **7** (positive mode)
88	**Fig. S79**. MS spectrum of **7** (negative mode)
89	**Fig. S80**. Depiction of **1** (blue) vs. **2** docked with the AG
89	**Fig. S81**. Depiction of **5** (cyan) vs. **7** docked with the AG

Spectroscopic data of compounds 8–12

Chikusetsu Saponin IVa (**8**) $[\alpha]_D^{25}$ -17.2 (*c* 0.1, MeOH)；UV (MeOH) λ_{max} (log ε) 202.0 (3.75)；CD (*c* 1.26 × 10^{-5} M, MeOH) $[\theta]_{202.9}$ 30353.7, $[\theta]_{220.3}$ -2915.7；^{1}H and ^{13}C NMR data see Table S2；(-)-ESIMS m/z 792.7 $[M-H]^-$

β-ecdysone (**9**) $[\alpha]_D^{25}$ +59.2 (*c* 0.1, MeOH)；UV (MeOH) λ_{max} (log ε) 242.0 (3.90)；CD (*c* 2.09 × 10^{-5} M, MeOH) $[\theta]_{225.3}$ 6985.0, $[\theta]_{252.1}$ -9378.2, $[\theta]_{324.5}$ 4456.6；^{1}H and ^{13}C NMR data see Table S3；(-)-ESIMS m/z 478.9 $[M-H]^-$

Makisterone A (**10**) $[\alpha]_D^{25}$ +144.5 (*c* 0.1, MeOH)；UV (MeOH) λ_{max} (log ε) 242.5 (4.03)；CD (*c* 2.02 × 10^{-5} M, MeOH) $[\theta]_{225.3}$ 10840.3, $[\theta]_{253.2}$ -10877.6, $[\theta]_{326.2}$ 4784.8；^{1}H and ^{13}C NMR data see Table S3；(+)-ESIMS m/z 517.0 $[M+Na]^+$

Stophylionoside D (**11**) $[\alpha]_D^{25}$ -47.0 (*c* 0.1, MeOH)；UV (MeOH) λ_{max} (log ε) 231.0 (4.25)；CD (*c* 2.59 × 10^{-5} M, MeOH) $[\theta]_{207.0}$ -131169, $[\theta]_{230}$ 87259.6, $[\theta]_{258}$ -63555.8；^{1}H and ^{13}C NMR data see Table S4；(+)-ESIMS m/z 409.0 $[M+Na]^+$

Austroside B (**12**) $[\alpha]_D^{25}$ -2.3 (*c* 0.1, MeOH)；UV (MeOH) λ_{max} (log ε) 232.0 (4.39)；CD (*c* 2.59 × 10^{-5} M, MeOH) $[\theta]_{207.3}$ -41869.6, $[\theta]_{230.5}$ 31998.4, $[\theta]_{258.8}$ -19045.7；^{1}H NMR data see Table S4；(+)-ESIMS m/z 409.0 [M+Na]

Table S1. HMBC spectroscopic data of aglycon moiety of compounds **1-7**[a] and NOESY data of **1**

Pos.	**1** [b]		**2** [b]	**3** [b]	**4** [b]	**5** [b]	**6** [b]	**7** [c]
	HMBC	NOESY	HMBC	HMBC	HMBC	HMBC	HMBC	HMBC
$1_{(eq)}$		$2_{(eq)}$						
$1_{(ax)}$		9						
2								
3	1′ (Ara)	5	24, 1′ (Ara)	1′ (Ara)		1′ (Ara)		1′ (Ara)
4								
5	4, 10, 24, 25	3, 9	4, 10	4, 6, 10, 24	4, 6, 10, 24	4, 6, 10, 24	10, 24	3,10, 24
$6_{(eq)}$		23						
$6_{(ax)}$		26						
7								
8								
9	1, 8, 10, 11, 14, 25, 26	$1_{(ax)}$, 5, 27	1, 8, 10, 11, 14, 25, 26	1, 8, 10, 11, 14, 25, 26	1, 8, 10, 11, 14, 25, 26	1, 8, 10, 11, 14, 25, 26	11, 14, 25	1, 8, 10, 11, 14, 25, 26
10								
11							9	
12	9, 14, 18	18	9, 14, 18	9, 14, 18	9, 14, 18	9, 14, 18	13, 14	9, 14, 18
13								
14								
$15_{(eq)}$								
$15_{(ax)}$		26						
$16_{(eq)}$								
$16_{(ax)}$		27						
17								
18	14, 16							12, 14, 28
19	18		18, 20, 30	18, 20, 30	18, 30	17, 18, 30	13, 17, 18, 20, 21, 30	18, 20, 21, 28, 30
20								
21								
22						16, 17, 20, 21, 28	16, 17, 18, 20, 21, 28	16, 17, 21, 28
23	3, 4, 5, 24	$6_{(eq)}$	3, 4, 5, 24	3, 4, 5, 24	3, 4, 5, 24	3, 4, 5, 24	3, 4, 5, 24	3, 4, 5, 24
24	3, 4, 5, 23	25	3, 4, 5, 23	3, 4, 5, 23	3, 4, 5, 23	3, 4, 5, 23	3, 4, 5, 23	3, 4, 5, 23
25	1, 5, 9, 10	24, 26	1, 5, 9, 10	1, 5, 9, 10	1, 5, 9, 10	1, 5, 9, 10	1, 5, 10	1, 5, 10
26	7, 8, 9, 14	$6_{(ax)}$, 12, $15_{(ax)}$, 25, 28	7, 8, 9, 14	7, 8, 9, 14	7, 8, 9, 14	7, 8, 9, 14	7, 8, 9, 14	7, 8, 9, 14
27	8, 13, 14, 15	9, $16_{(ax)}$	8, 13, 14, 15	8, 13, 14, 15	8, 11, 14, 15	8, 13, 14, 15	8, 13, 14, 15	8, 13, 14, 15
28	16, 22		16, 22	18, 22		16, 22	16, 17, 18, 22	16, 17, 18, 22
29	19, 20, 21, 30		19, 20, 21, 30	19, 20, 21, 30	19, 20, 21, 30	19, 21, 30	19, 20, 21, 30	19, 20, 21, 30
30	19, 20, 21, 29		19, 20, 21, 29	19, 20, 21, 29	19, 20, 21, 22, 29	19, 21, 29	19,20, 21, 29	19, 20, 21, 29
30-OMe	30							

[a] NMR spectra were measured on Bruker AV-III-600.
[b] Using CD_3OD as solvent.
[b] Using CD_3OD+D_2O = 1:1 as solvent.

Table S2. ^{1}H, ^{13}C and HMBC spectroscopic data of compound **8** (CD_3OD)

Position	**8** [a]		**Chikusetsu Saponin IVa (400 MHz HNMR CD_3OD)**		$\Delta\delta_H$	$\Delta\delta_C$
	δ_H m (J / Hz)	δ_C	δ_H m (J / Hz)	δ_C		
1		39.7 t	1.60, 0.98	40.3		-0.6
2		26.9 t	1.97, 1.69	27.1		-0.2
3	3.16 (dd, 11.8, 4.4)	90.9 d	3.19	91.3	0	-0.4
4		40.1 s	-	40.7		-0.6
5		57.0 d	0.77	57.4		-0.4
6		19.3 d	1.54, 1.39	19.6		-0.3
7		33.9 d	1.48, 1.31	34.2		-0.3
8		40.7 s	-	41.0		-0.3
9		[b]	1.57	49.4		
10		37.8 d	-	38.4		-0.6
11		24.5 t	1.90, 1.90	24.9		-0.4
12	5.24 (t, 3.4)	123.8 d	5.27	124.1	-0.03	-0.3
13		144.7 s	-	145.3		-0.6
14		42.9 s	-	43.3		-0.4
15		28.9 d	1.80, 1.08	29.2		-0.3
16	2.04 (dt, 3.7, 14.1)$_{(ax)}$	24.0 t	2.04, 1.71	24.3	0	-0.3
17		47.9 s	-	48.3		-0.4
18	2.84 (dd, 13.9, 4.3)	42.6 d	2.85	43.1	-0.01	-0.5
19		47.1 t	1.70, 1.15	47.5		-0.4
20		31.5 s	-	31.7		-0.2
21		34.9 t	1.39, 1.21	35.2		-0.3
22		33.1 t	1.73, 1.61	33.5		-0.4
23	1.03 (s)	28.4 q	1.06	28.9	-0.03	-0.5
24	0.84 (s)	16.9 q	0.85	17.3	-0.01	-0.4
25	0.94 (s)	16.0 q	0.95	16.3	-0.01	-0.3
26	0.78 (s)	17.7 q	0.80	18.1	-0.02	-0.4
27	1.14 (s)	26.2 q	1.15	26.7	-0.01	-0.5
28		178.0 s	-	178.4		-0.4
29	0.90 (s)	33.4 q	0.91	33.9	-0.01	-0.5
30	0.92 (s)	23.9 q	0.93	24.4	-0.01	-0.5
1'	4.35 (d, 7.8)	106.9 d	4.38 (d, 7.8)	106.7	-0.03	+0.2
2'	3.22 (t, 8.3)	75.3 d	3.28	75.5	-0.06	-0.2
3'	3.45 (t, 9.6)	73.4 d	3.48	73.7	-0.03	-0.3
4'	3.35 (t, 9.4)	78.3 d	3.45	78.6	-0.10	-0.3
5'	3.67 (d, 9.6)	76.6 d	3.64	76.6	+0.03	0
6'		[c]	-	172.4	-	-
1''	5.36 (d, 8.2)	95.7 d	5.40 (d, 7.7)	96.1	-0.04	-0.4
2''	3.30 (t, 8.3)	73.9 d	3.35	74.1	-0.05	-0.2
3''	3.49 (t, 9.4)	77.8 d	3.43	78.2	+0.06	-0.4
4''	3.33 (m)	71.1 d	3.38	71.2	-0.05	-0.1
5''	3.33 (m)	78.7 d	3.38	78.8	-0.05	-0.1
6''	3.80 (dd, 11.5, 1.5)	62.4 t	3.84	62.7	-0.04	-0.3
	3.67 (dd, 12.1, 4.8)		3.71		-0.04	

[a] NMR spectra were measured on Bruker AV-III-600.
[b] The signal is overlapped with solvent peak.
[c] Signal are not detectable

Table S3. ^{1}H and ^{13}C spectroscopic data of **9** and **10** (CD_3OD)

Position	**9** [a]		**10** [a]	
	δ_H m (J / Hz)	δ_C	δ_H m (J / Hz)	δ_C
1		37.3 t		37.1 t
2	3.83 (dt, 11.5 ,3.2)	68.7 d	3.83 (brd, 11.7)	68.6 d
3	3.94 (d, 2.0)	68.5 d	3.97 (brs)	68.4 d
4		32.8 t		32.6 t
5	2.36 m	51.8 d	2.36 (dd, 12.8, 4.5)	51.2 d
6		206.4 s		208.1 s
7	5.80 (d, 2.0)	122.1 d	5.82 (brs)	122.1 d
8		167.9 s		168.4 s
9	3.14 (t, 8.3)	35.1 d	3.14 (t, 7.3)	34.5 d
10		39.3 s		39.3 s
11		21.5 t		21.4 t
12		31.8 t		31.6 t
13		b		b
14		85.2 s		85.4 s
15		32.5 t		32.4 t
16		21.5 t		21.3 t
17	2.38 (m)	50.5 d		50.3 d
18	0.88 (s)	18.0 q	0.88 (s)	18.1 q
19	0.96 (s)	24.4 q	0.96 (s)	24.3 q
20		77.9 s		78.3 s
21	1.18 (s)	21.0 q	1.13 (s)	20.9 q
22	3.33	78.4 d	3.47 (brs, 11.0)	75.3 d
23		27.3 t		34.2 t
24		42.4 t		41.5 d
25		71.3 s		74.0 s
26	1.19 (s)	28.9 q	1.16 (s)	26.0 q
27	1.20 (s)	29.7 q	1.19 (s)	27.4 q
28			0.92 (d, 6.8)	14.8 q

[a] NMR spectra were measured on Bruker AV-III-600.
[b] The signals are overlapped with solvent peak.

Table S4. ^{1}H and ^{13}C spectroscopic data of **11** and **12** (CD_3OD)

Position	**11** [a]		**12** [a]
	δ_H m (J / Hz)	δ_C	δ_H m (J / Hz)
1		37.0 s	
2	2.09 (dd, 11.6 ,1.8)$_{(eq)}$<br>1.47 (dd, 12.2 ,4.7)$_{(ax)}$	46.7 t	2,05 (brd, 12,8)<br>1.42 (d, 4.2) [b]
3	4.34 (tt, 11.3, 3.7)	72.6 d	4.34 (tt, 11.6 4.0)
4	2.36 (dd, 12.2, 1.8)$_{(eq)}$<br>1.45 (dd, 4.7, 12.2)$_{(ax)}$	48.2 t	2.33 (brd, 13.1)<br>1.37 (d, 4.1) [b]
5		72.4 s	
6		120.1 s	
7		200.8 s	
8	5.82 (s)	101.2 d	5,90 (s)
9	3.14 (t, 8.3)	211.4 s	[c]
10	2.18 (s)	26.5 q	2.27 (s)
11	1.32 (s)	29.4 q	1.12 (s)
12	1.11 (s)	32.2 q	1.43 (s)
13	1.40 (s)	30.8 q	1.37 (s)
1'	4.43 (d, 7.8)	102.7 d	4.42 (d, 7.8)
2'	3.13 (t, 8.5)	75.1 d	3.13 (dd, 9.2, 8.0)
3'		78.2 d	
4'		71.7 d	
5'		77.9 q	
6'	3.86 (brd, 11.9)<br>3.68 (dd, 12.0, 4.2)	62.8 t	3.86 (dd, 11.9, 1.7)<br>3.68 (dd, 11.9, 5.1)

[a] NMR spectra were measured on Bruker AV-III-600.
[b] The signals are overlapped with solvent peak and could calculated coupling constant.
[c] The signal is overlapped with solvent peak

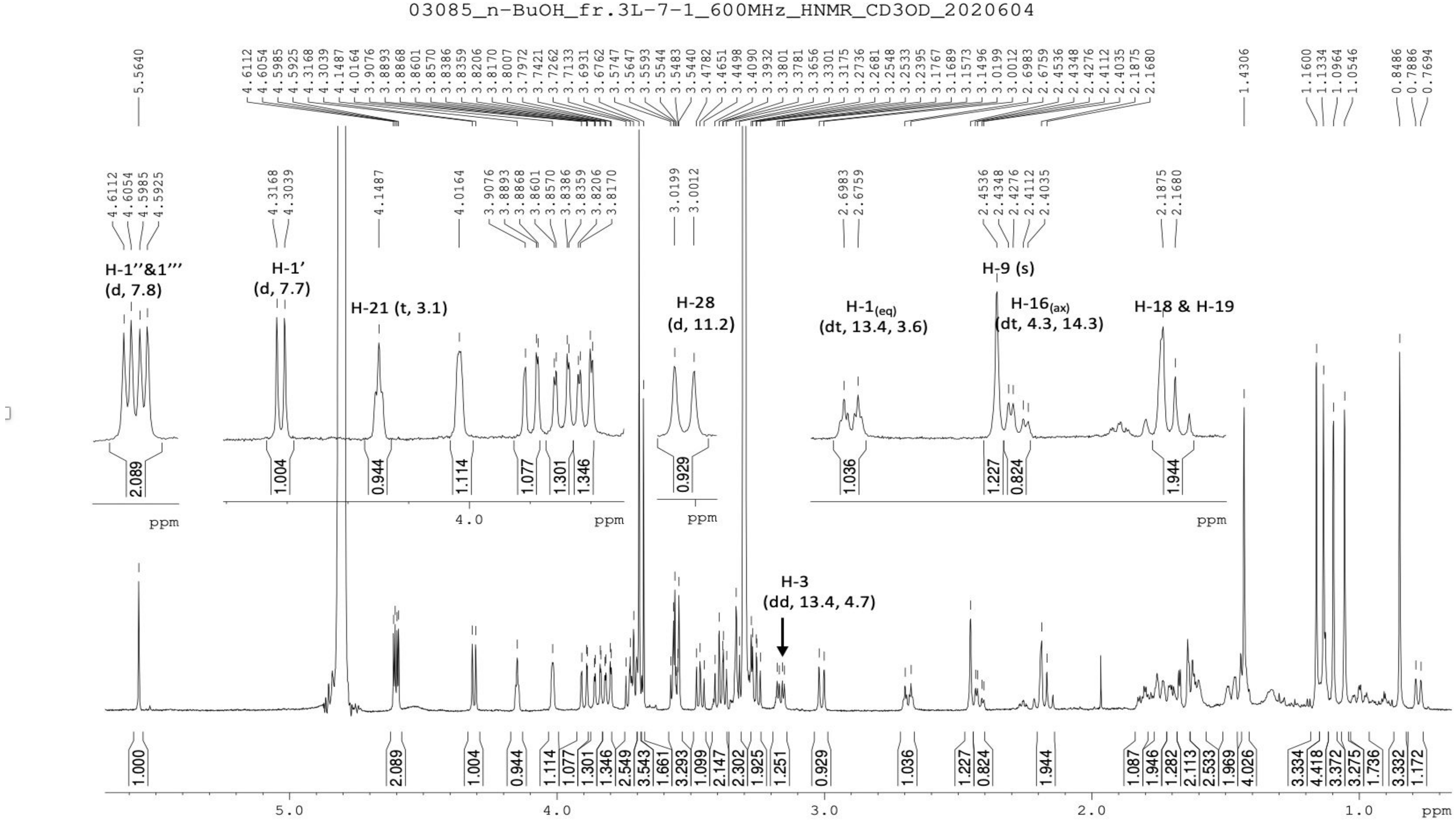

Fig. S1. ^{1}H NMR spectrum of **1** (CD_3OD, 600 MHz)

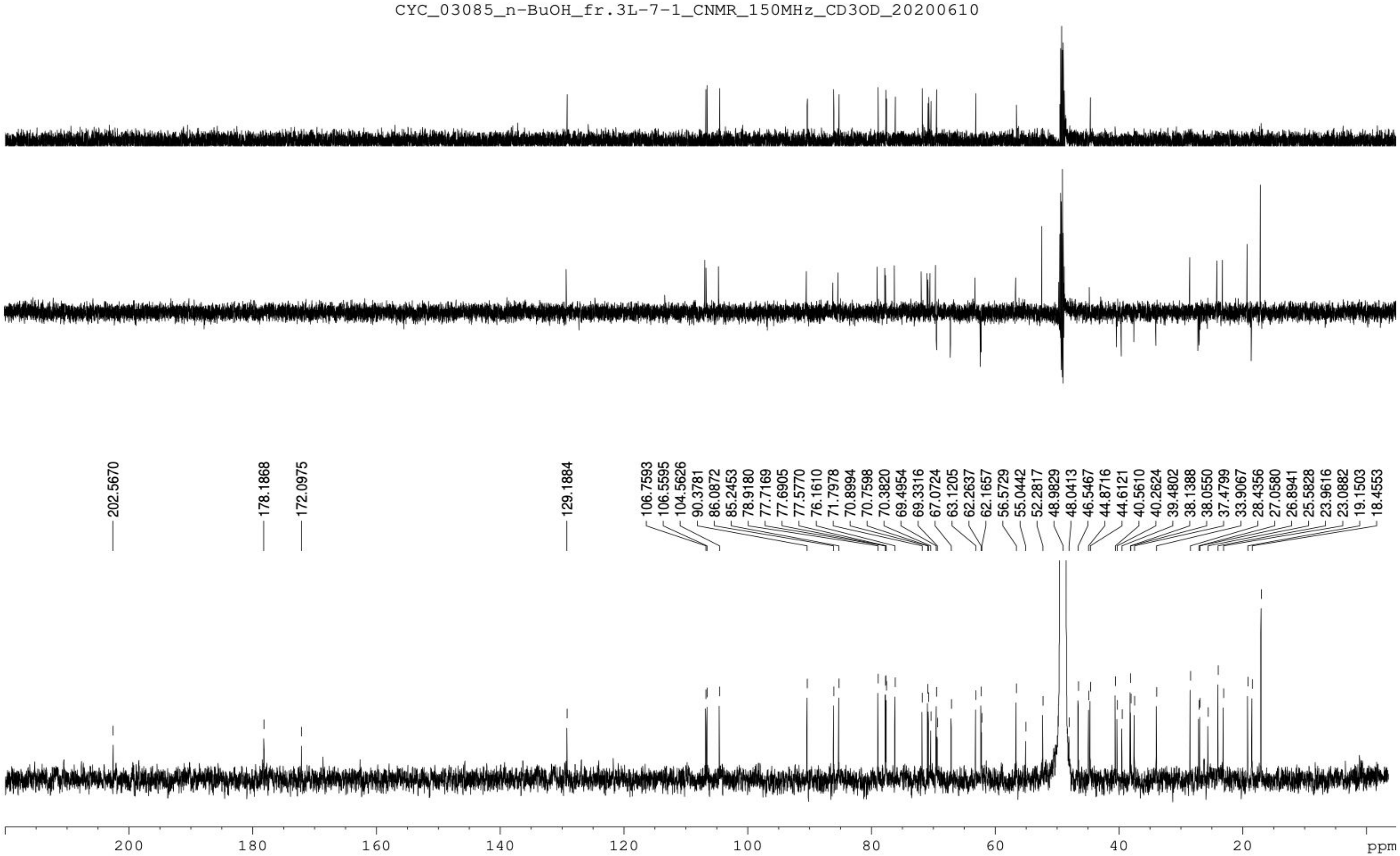

Fig. S2. ^{13}C NMR spectrum of **1** (BBD, bot.; DEPT-135, mid.; DEPT-90, top) (CD_3OD, 150 MHz)

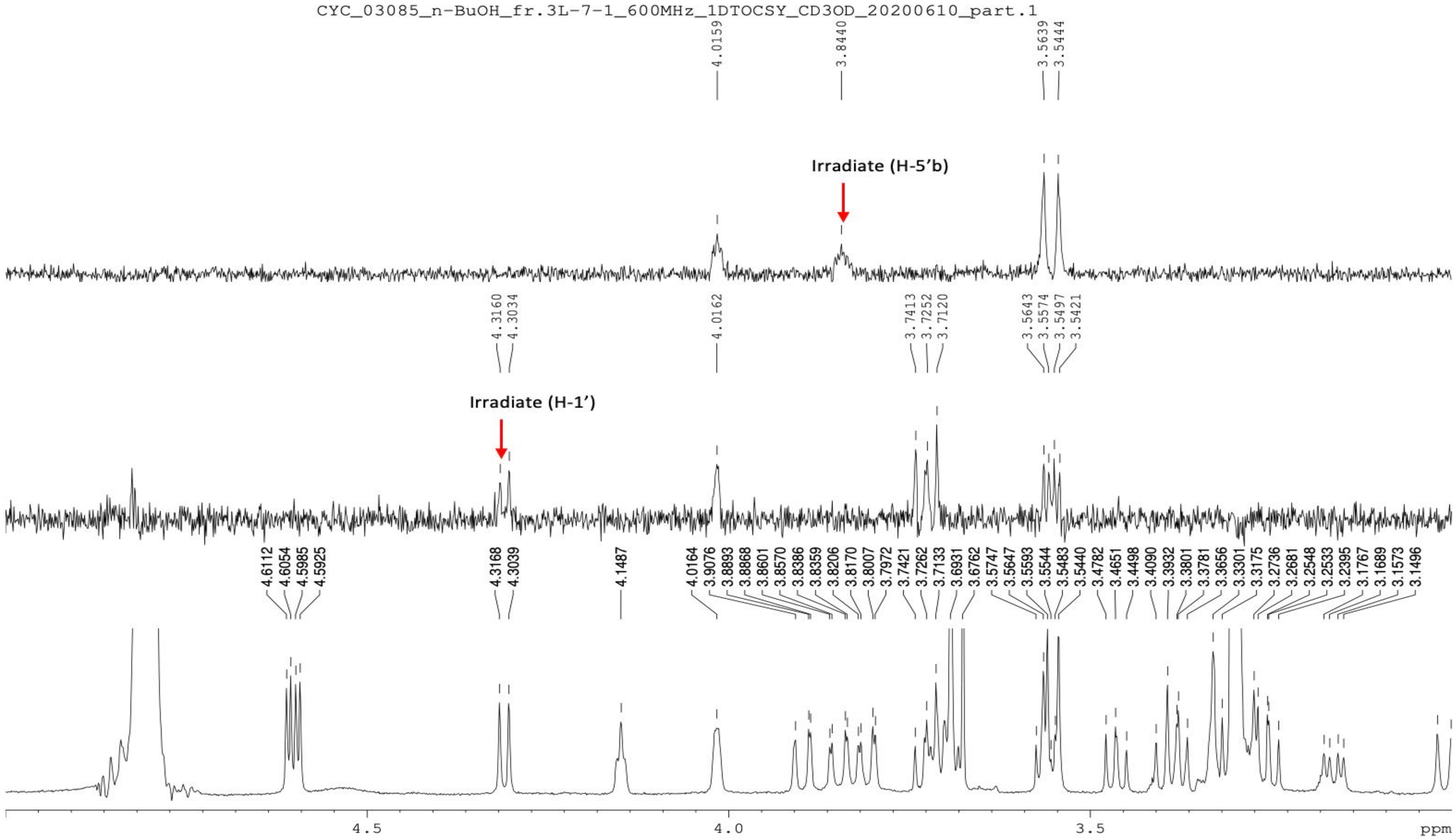

Fig. S3. 1D-TOCSY spectrum of **1** (CD_3OD, 600 MHz) (H-1′ &5′b)

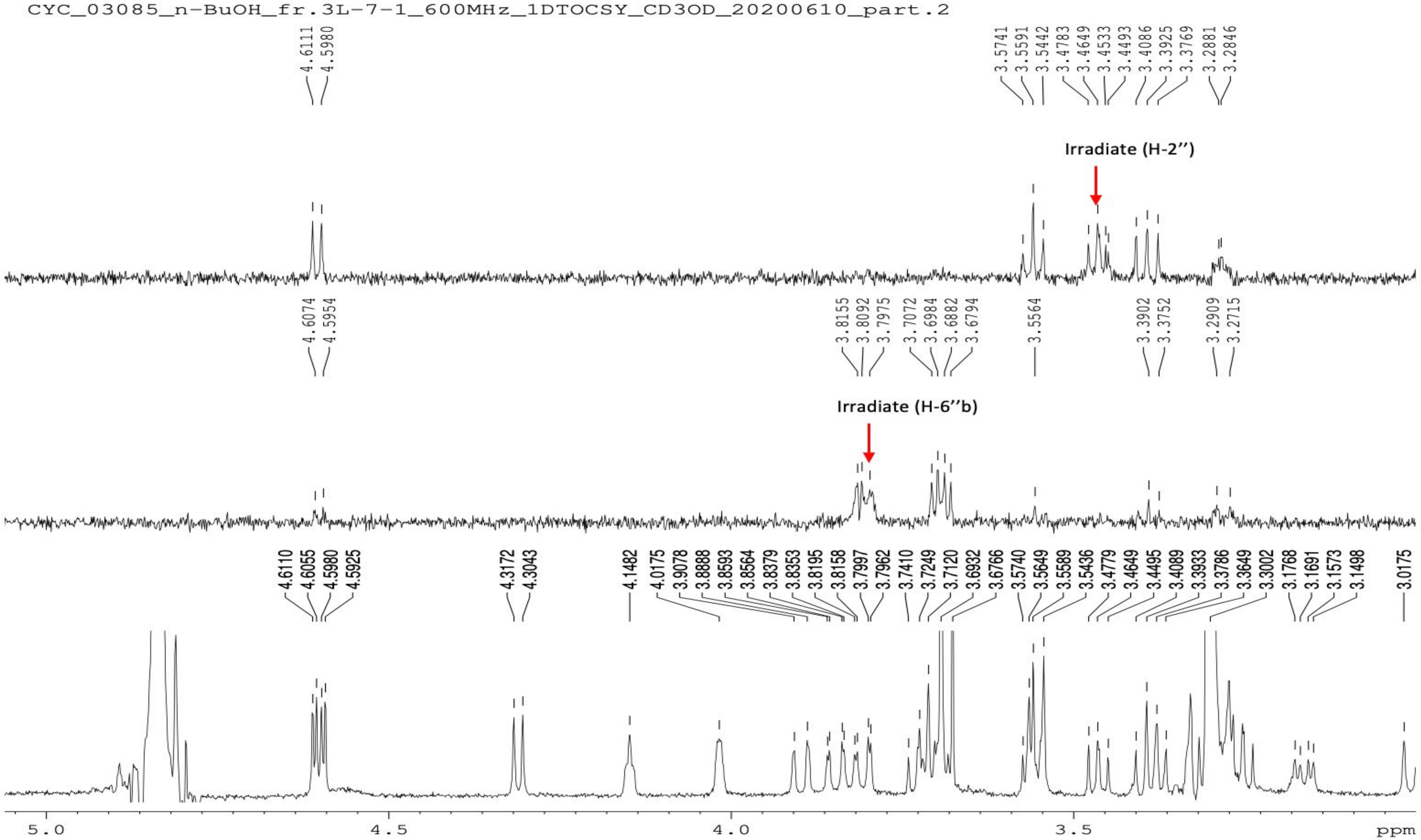

Fig. S4. 1D-TOCSY spectrum of **1** (CD_3OD, 600 MHz) (H-2″ &6″ b)

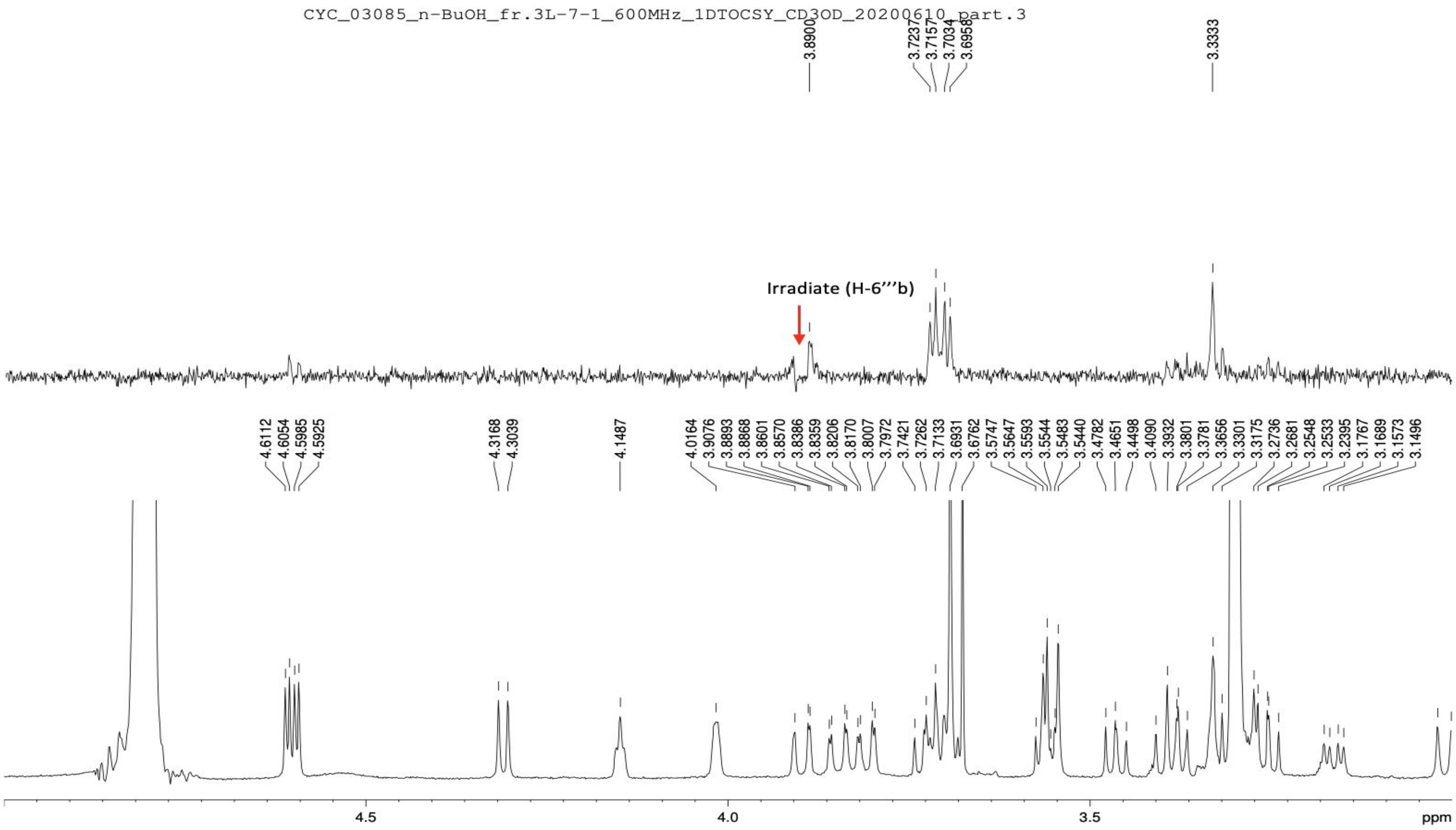

Fig. S5. 1D-TOCSY spectrum of **1** (CD_3OD, 600 MHz) (H-6‴ b)

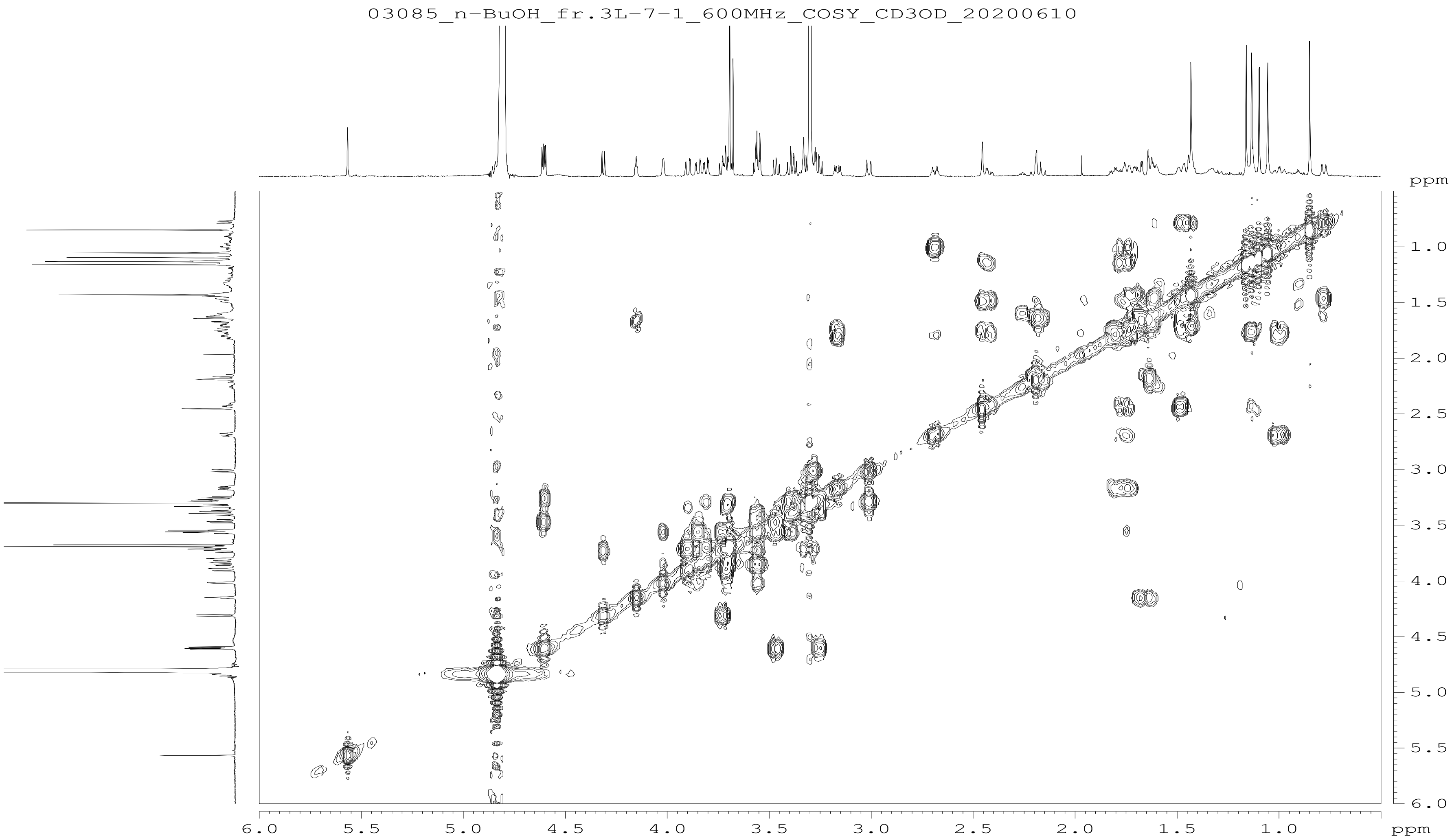

Fig. S6. COSY spectrum of **1** (CD_3OD, 600 MHz)

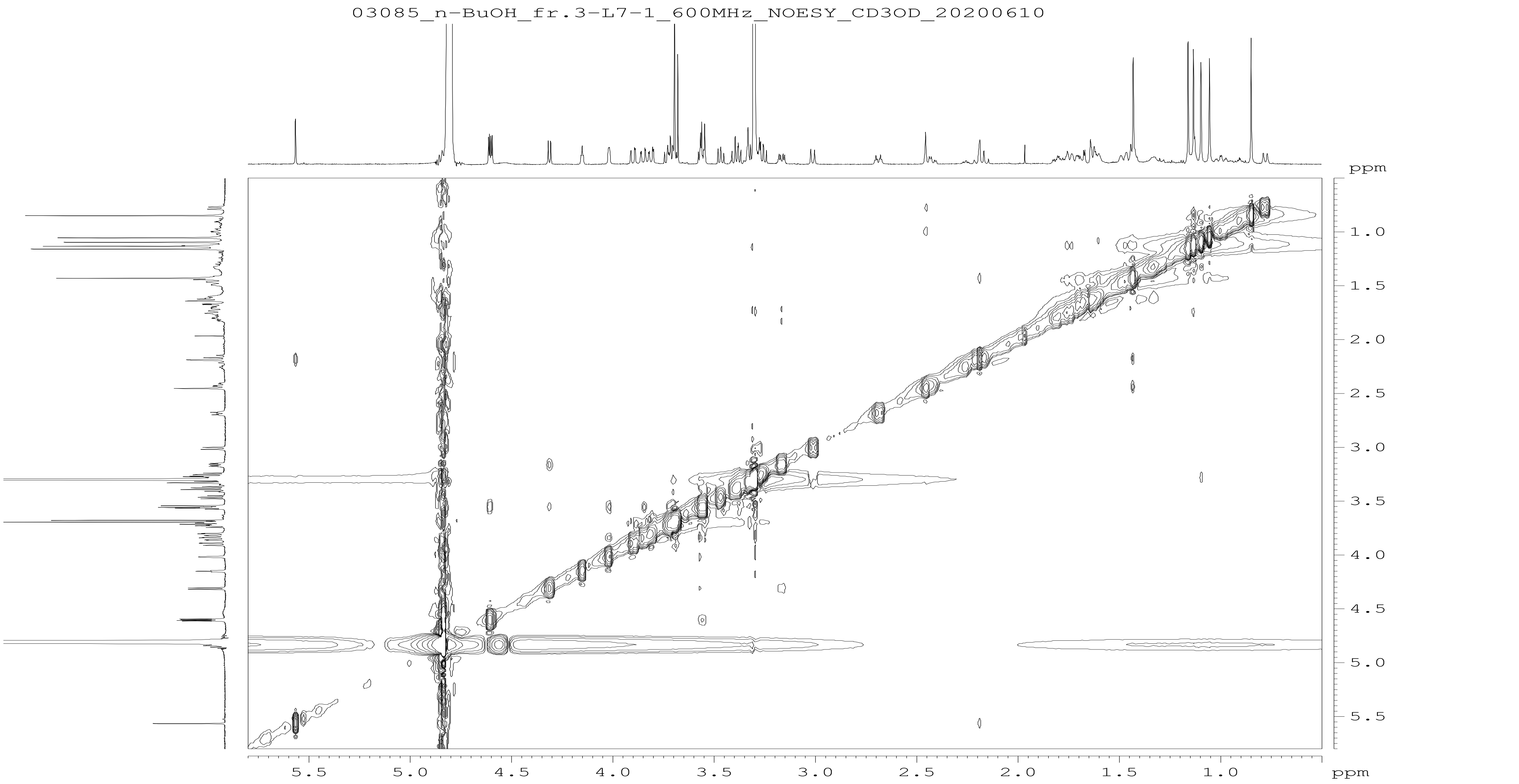

Fig. S7. NOESY spectrum of **1** (CD_3OD, 600 MHz)

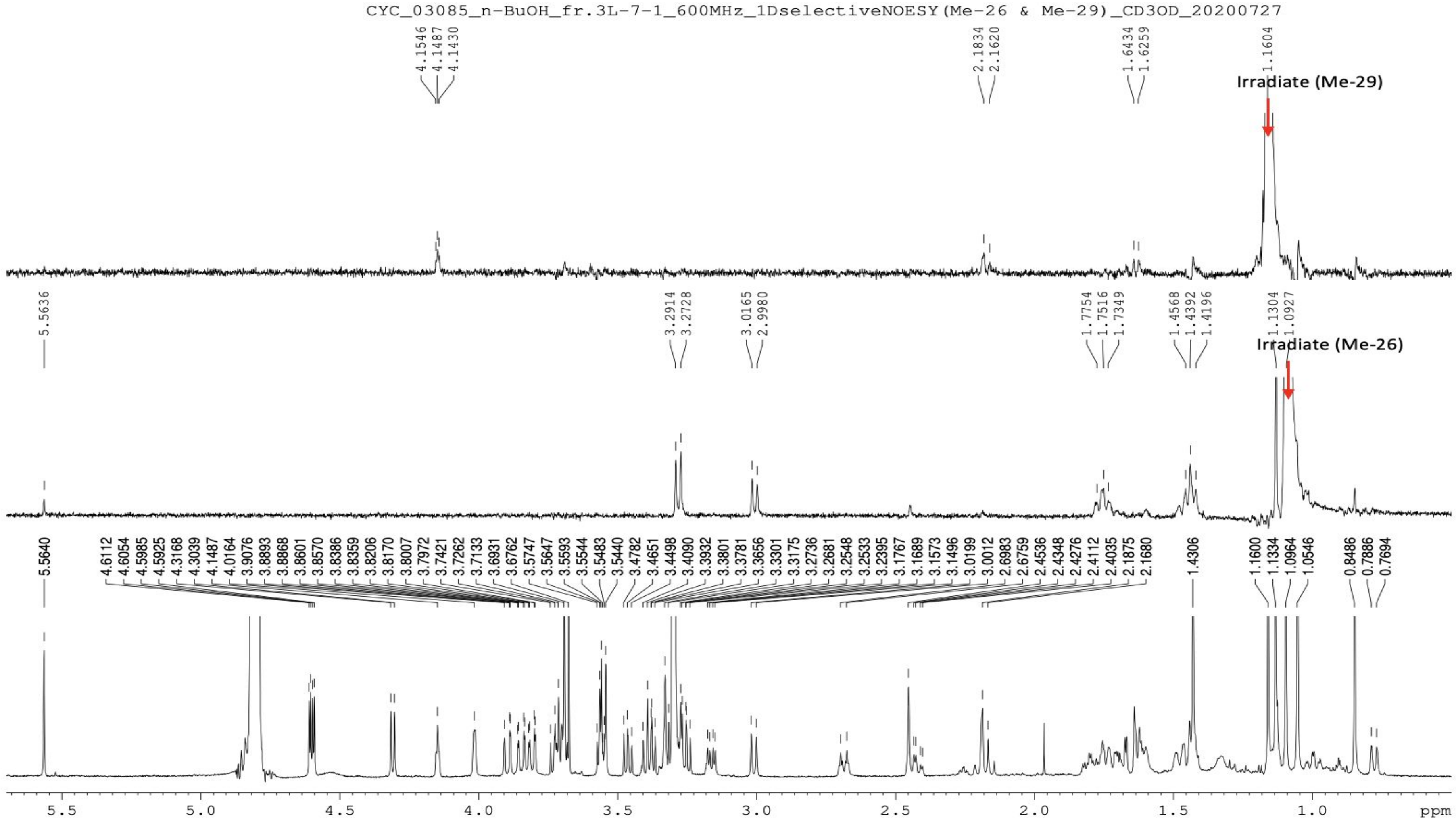

Fig. S8. 1D-NOESY spectrum of **1** (CD_3OD, 600 MHz) (Me-26&Me-29)

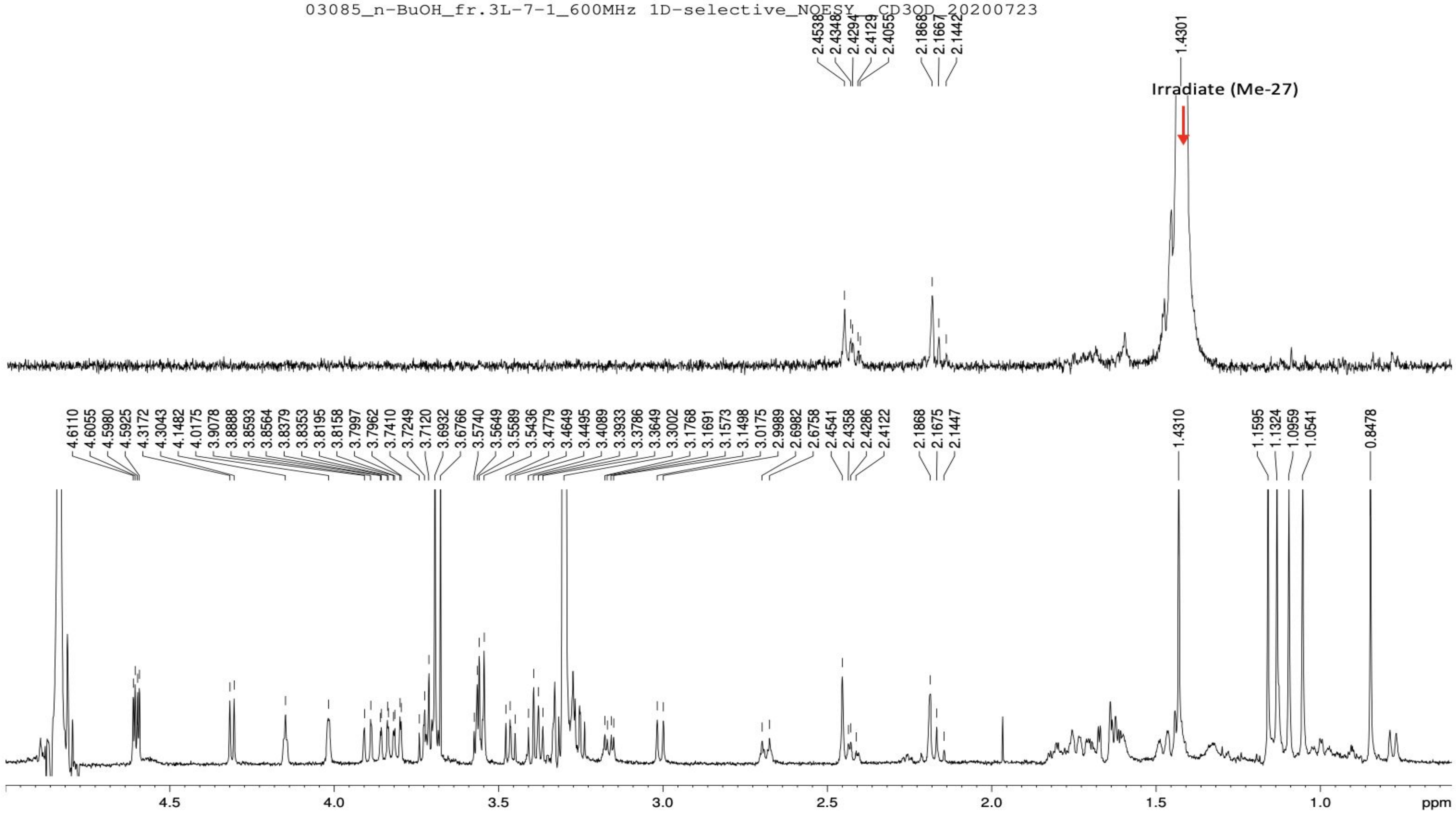

Fig. S9. 1D-NOESY spectrum of **1** (CD_3OD, 600 MHz) (Me-27)

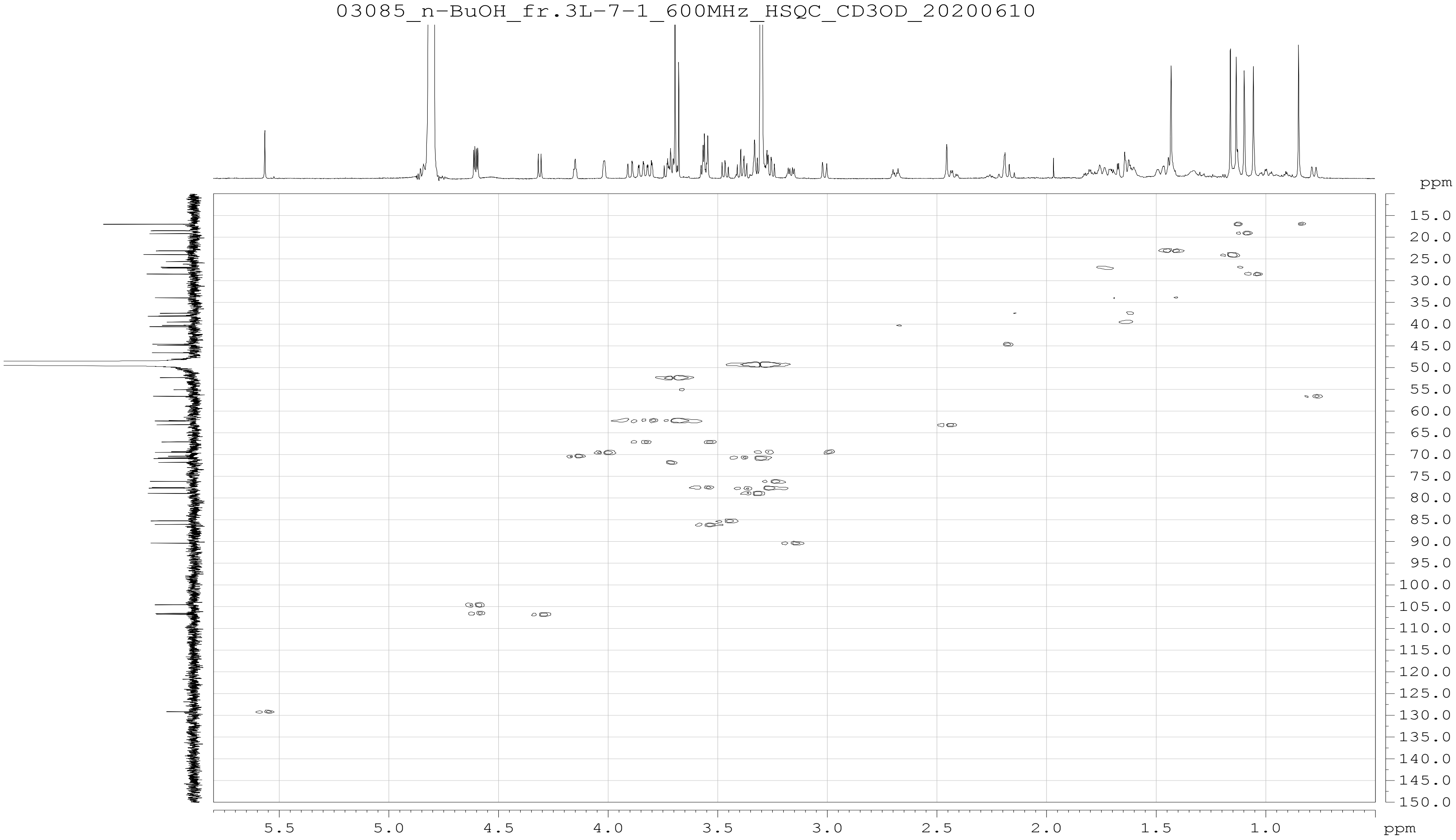

Fig. S10. HSQC spectrum of **1** (CD_3OD, 600 MHz)

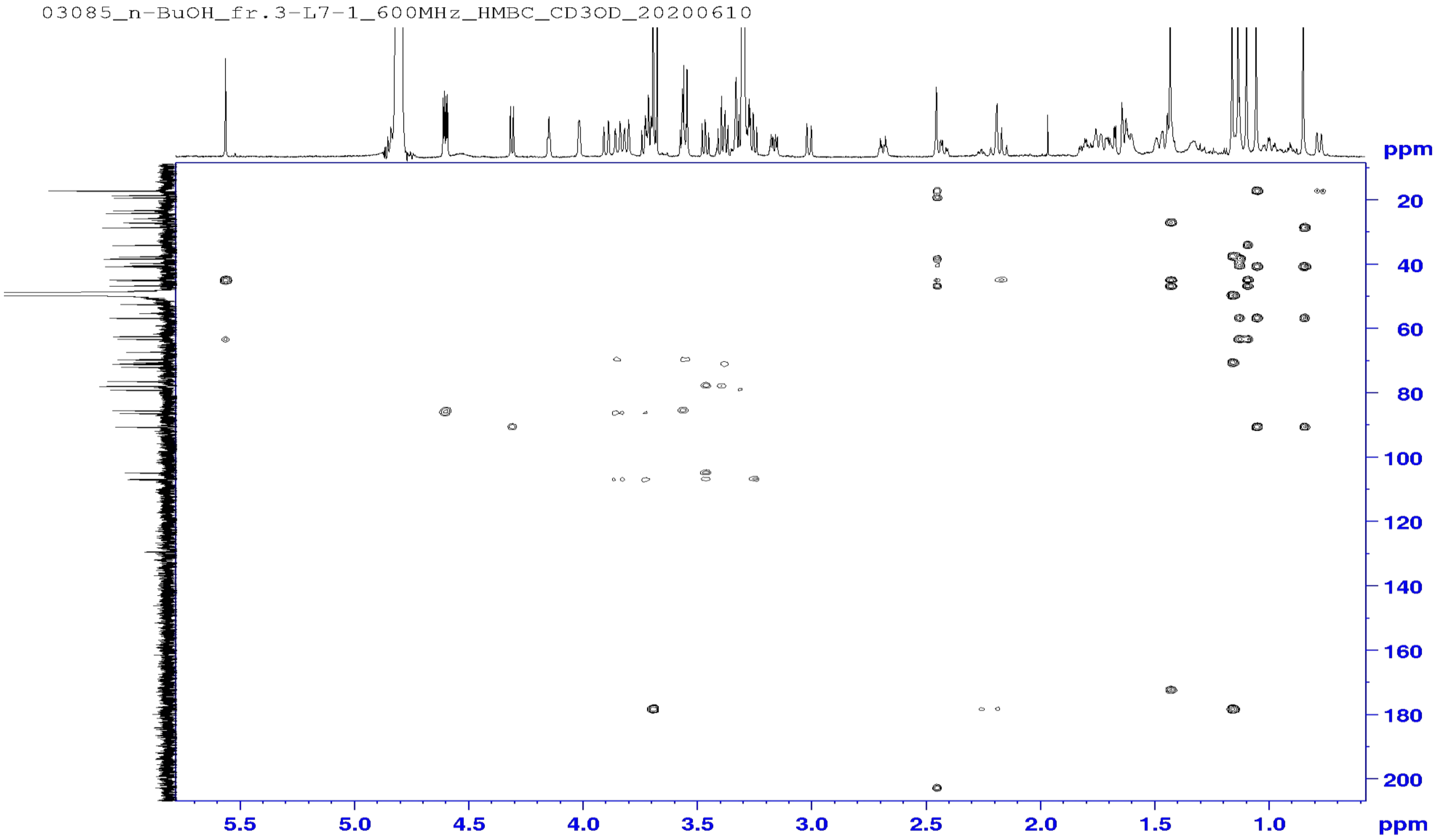

Fig. S11. HMBC spectrum of **1** (CD_3OD, 600 MHz)

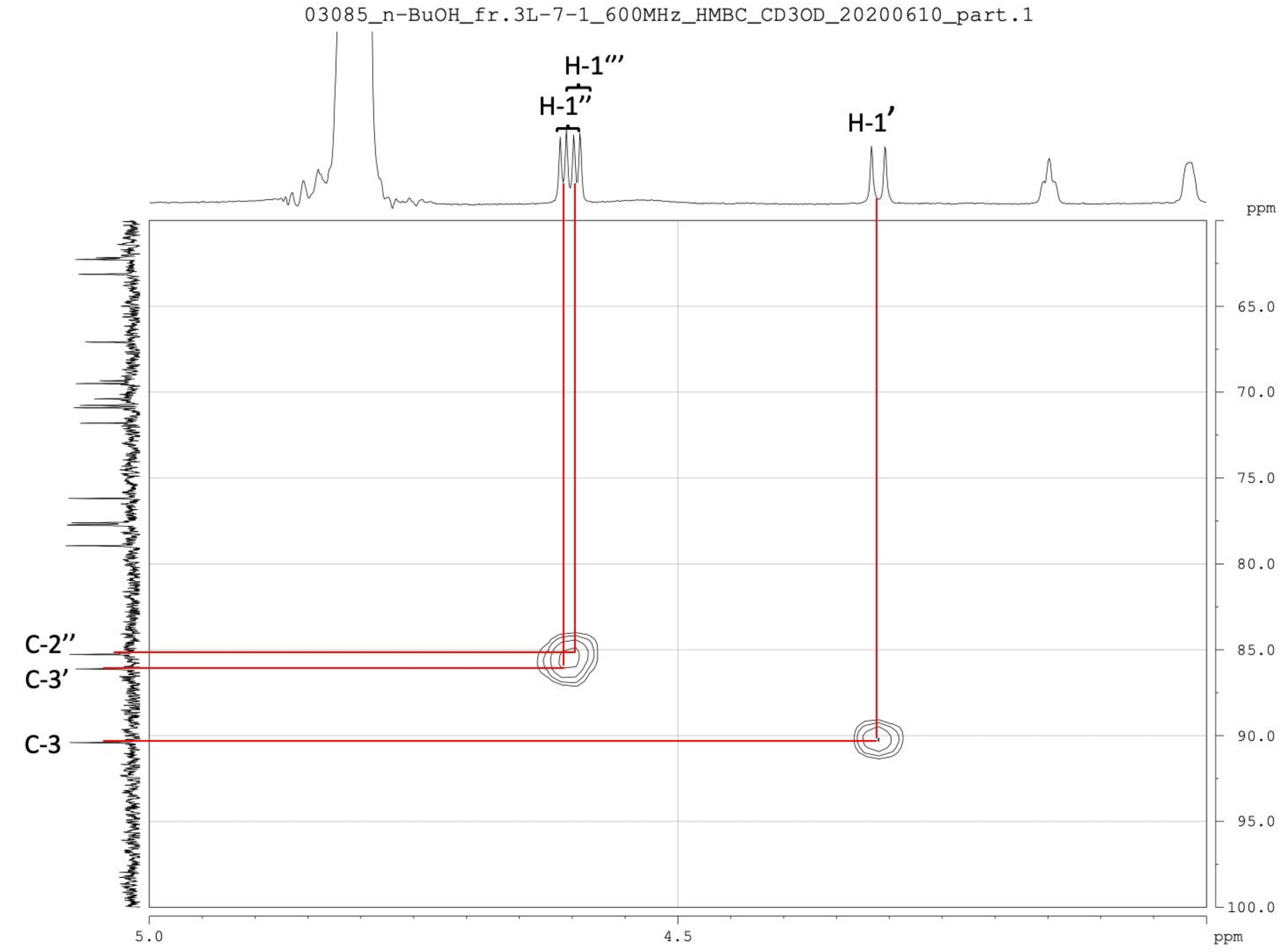

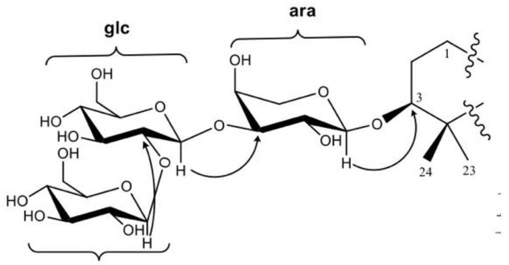

1

Fig. S12. HMBC spectrum of **1** (CD_3OD, 600 MHz) (glycosidic linkages)

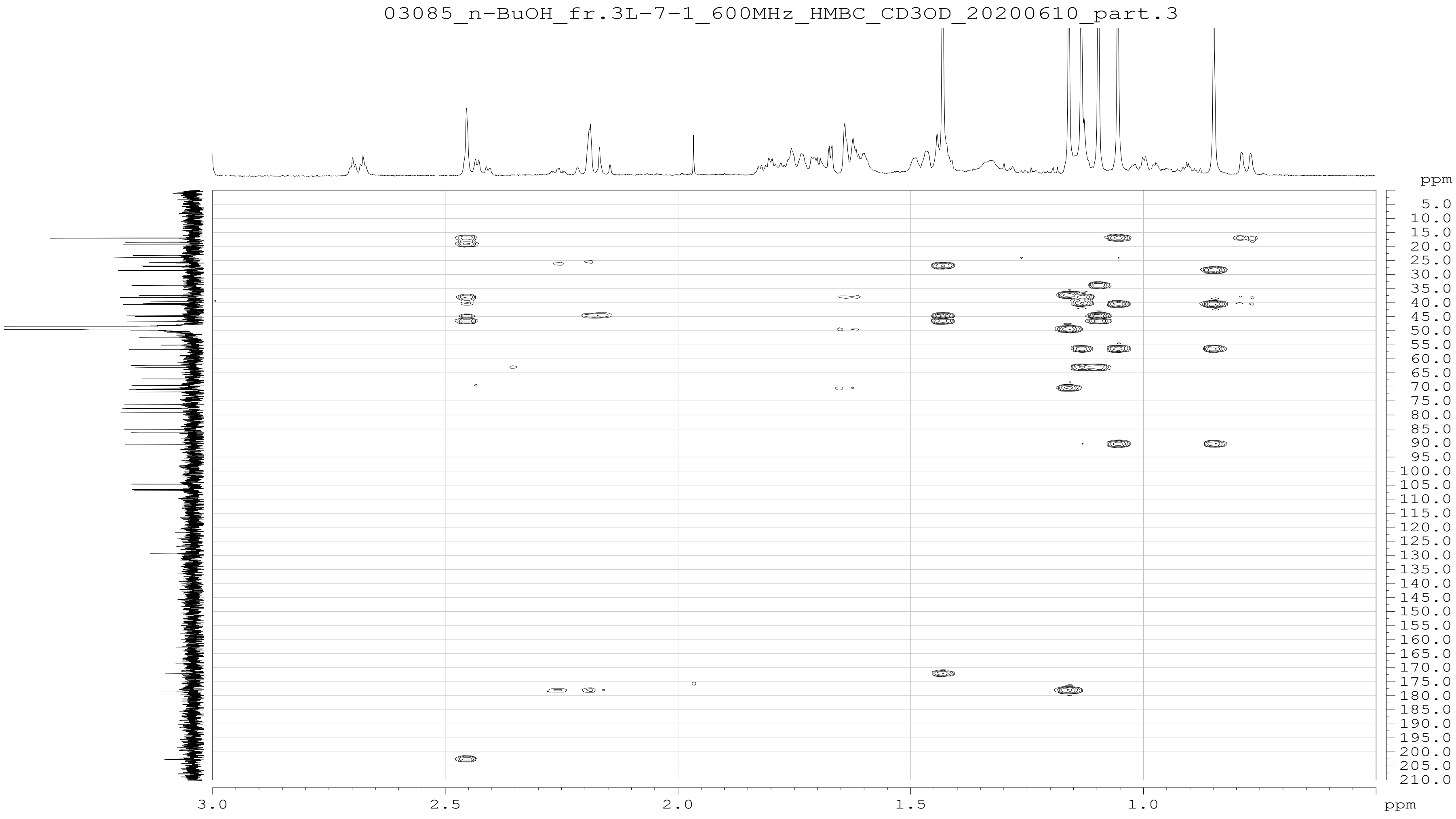

Fig. S13. HMBC spectrum of **1** (CD_3OD, 600 MHz) (aliphatic region)

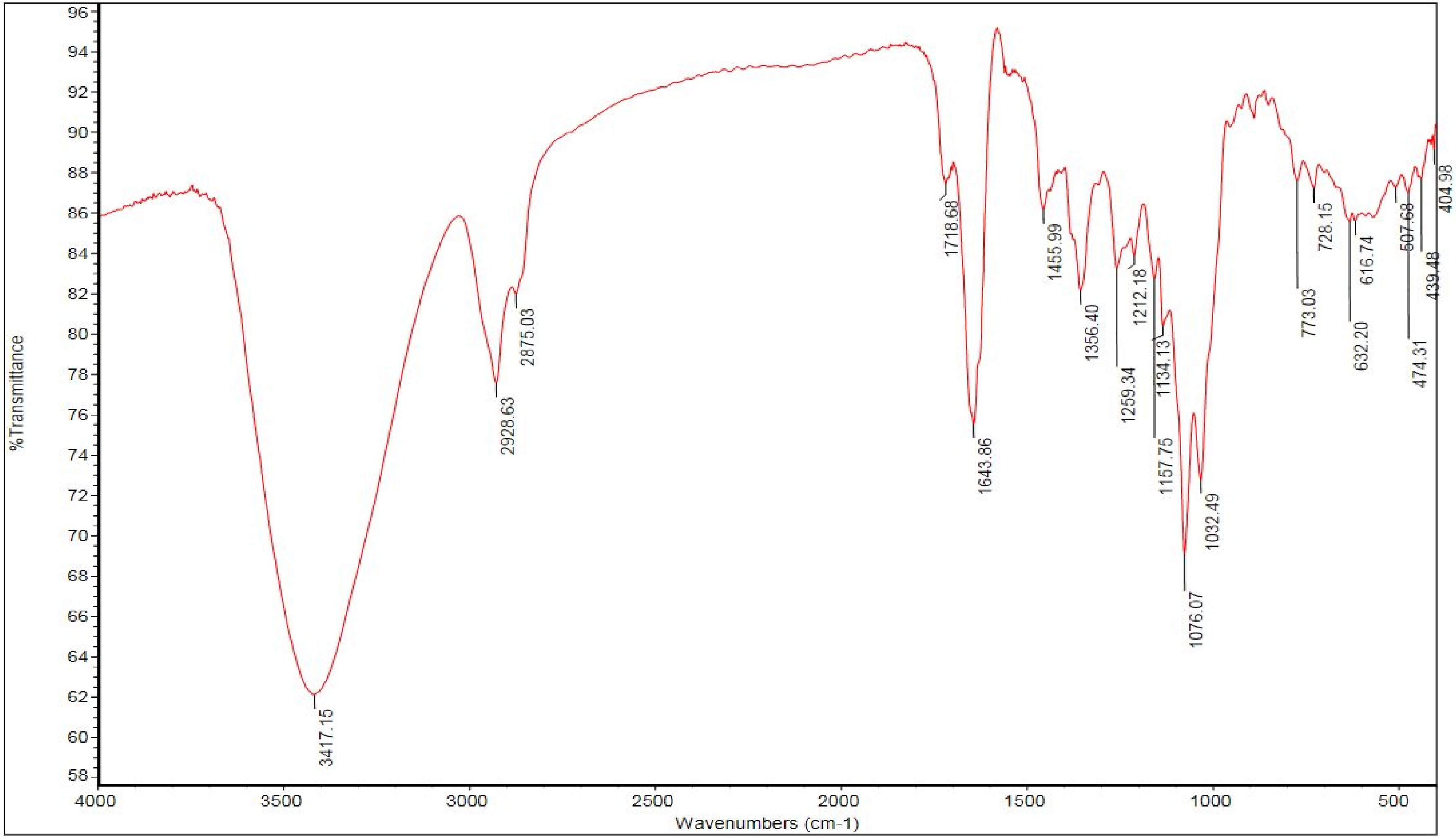

Fig. S14. IR spectrum of **1**

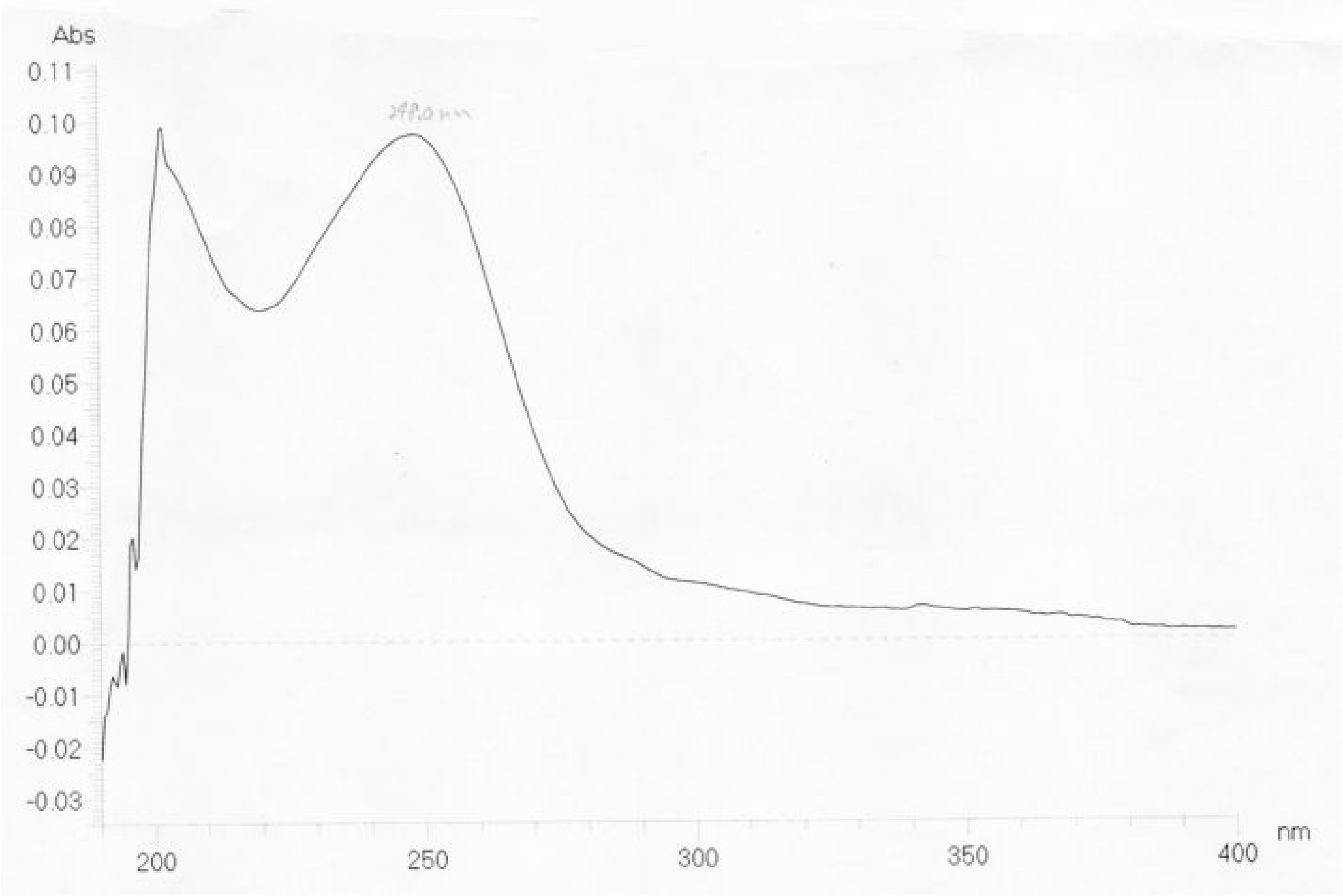

Fig. S15. UV spectrum of **1**

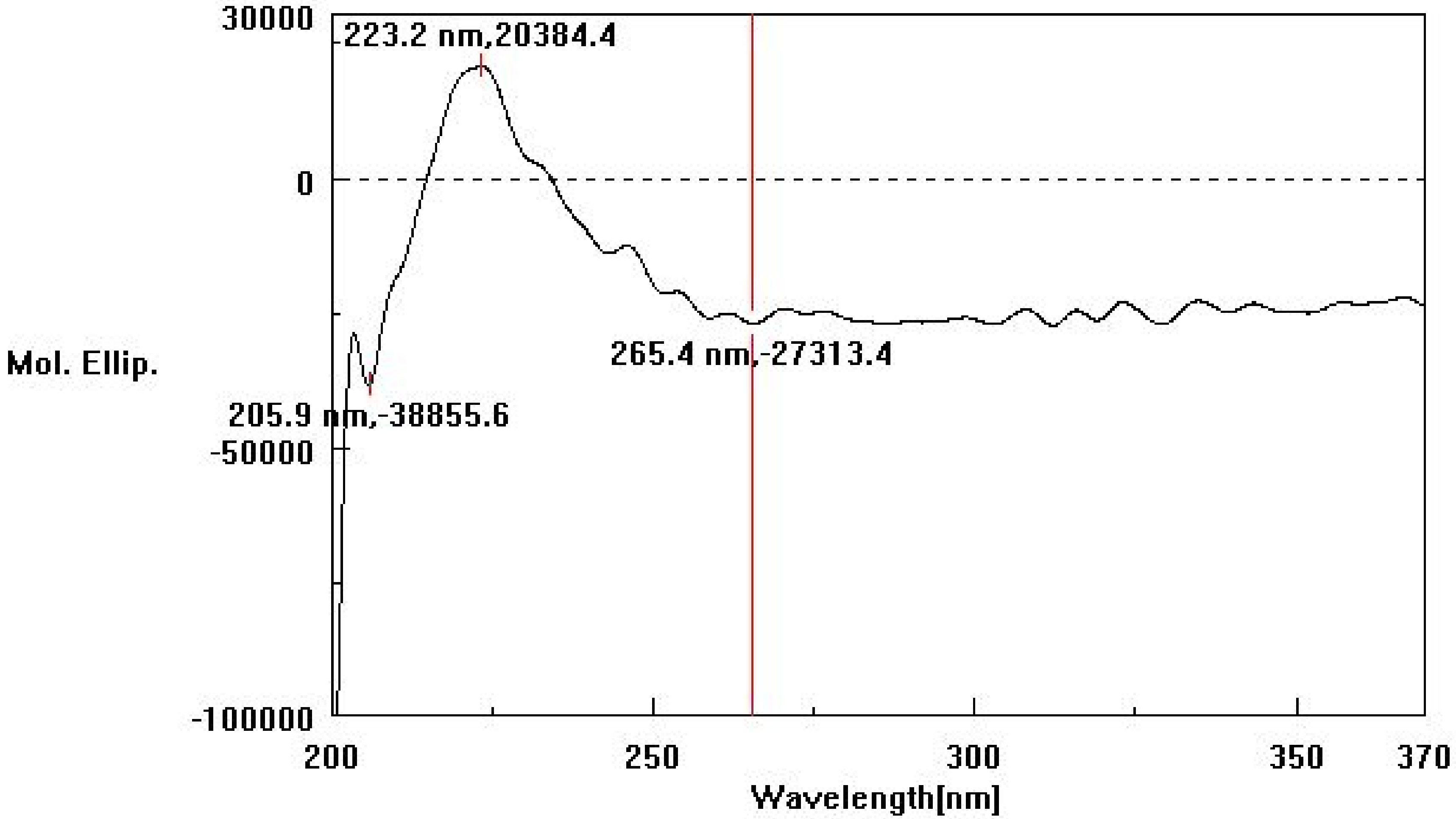

Fig. S16. CD spectrum of **1**

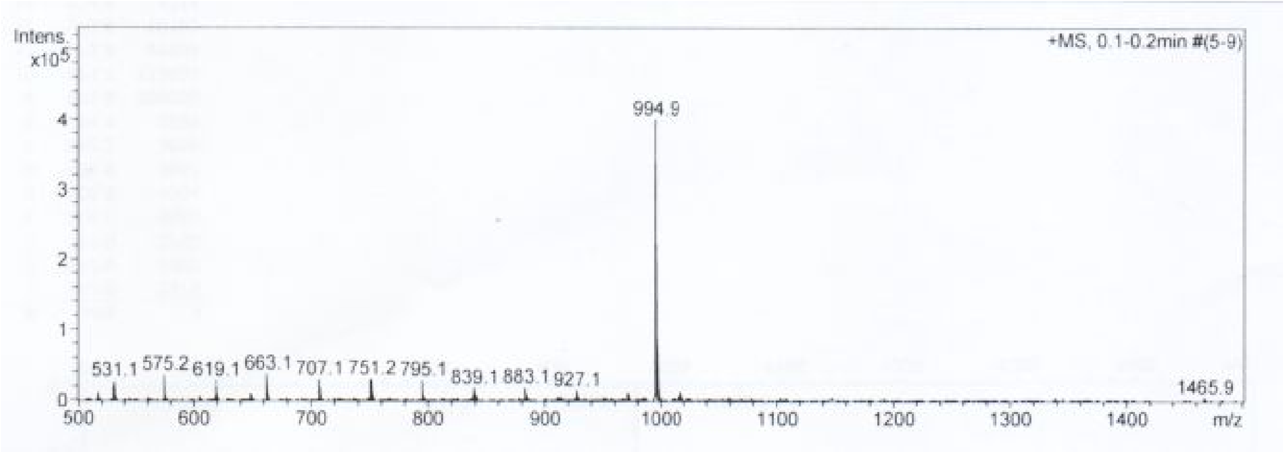

Fig. S17. MS spectrum of **1** (positive mode)

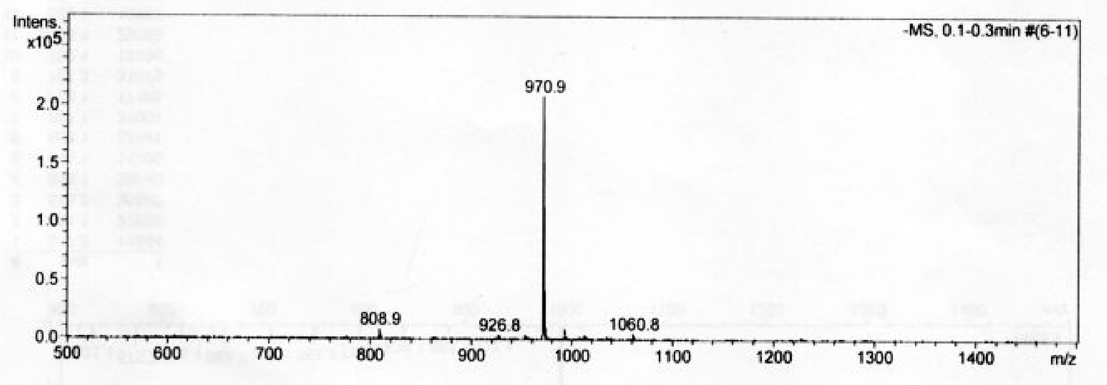

Fig. S18. MS spectrum of **1** (negative mode)

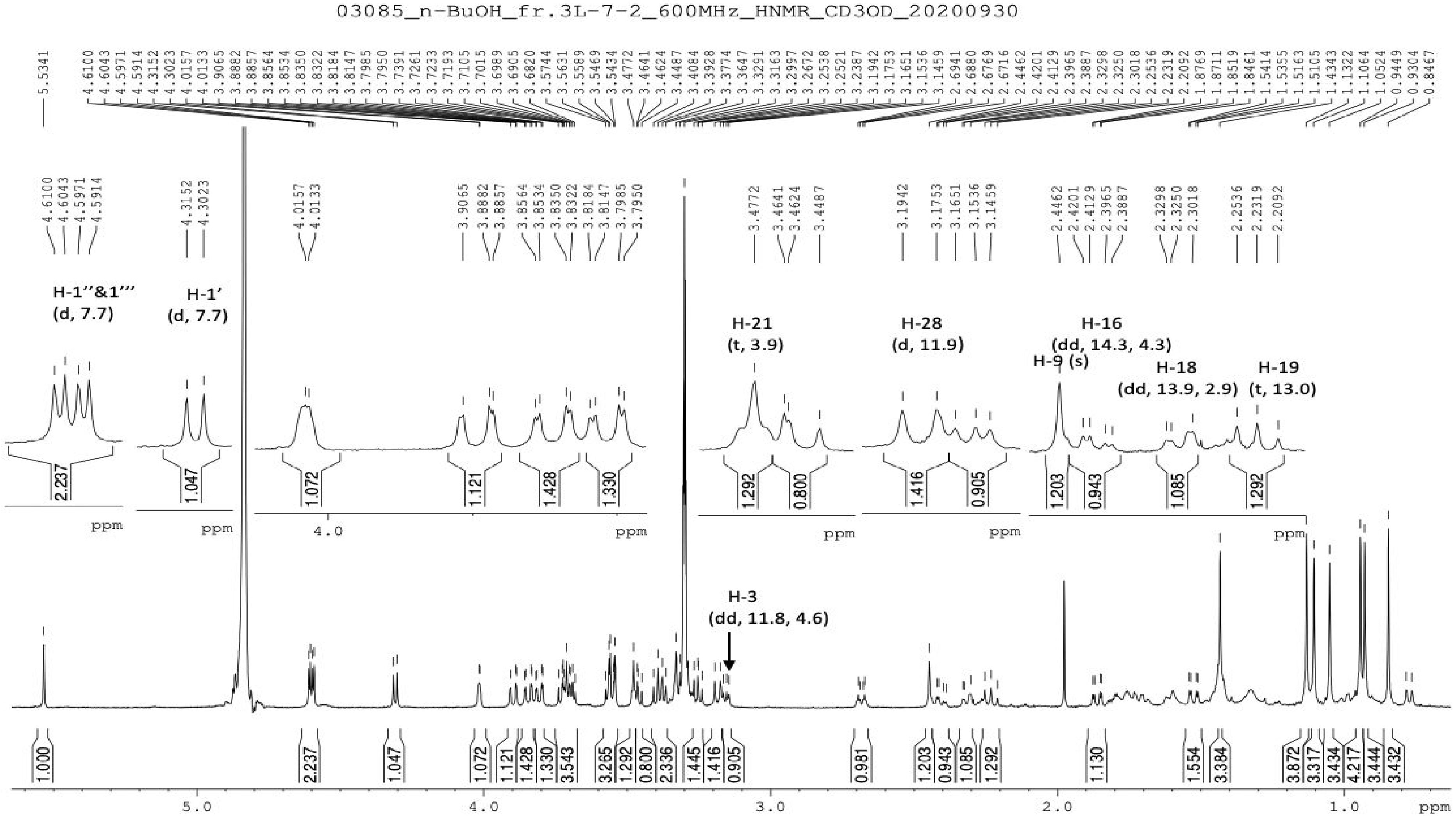

Fig. S19. ^{1}H NMR spectrum of **2** (CD_3OD, 600 MHz)

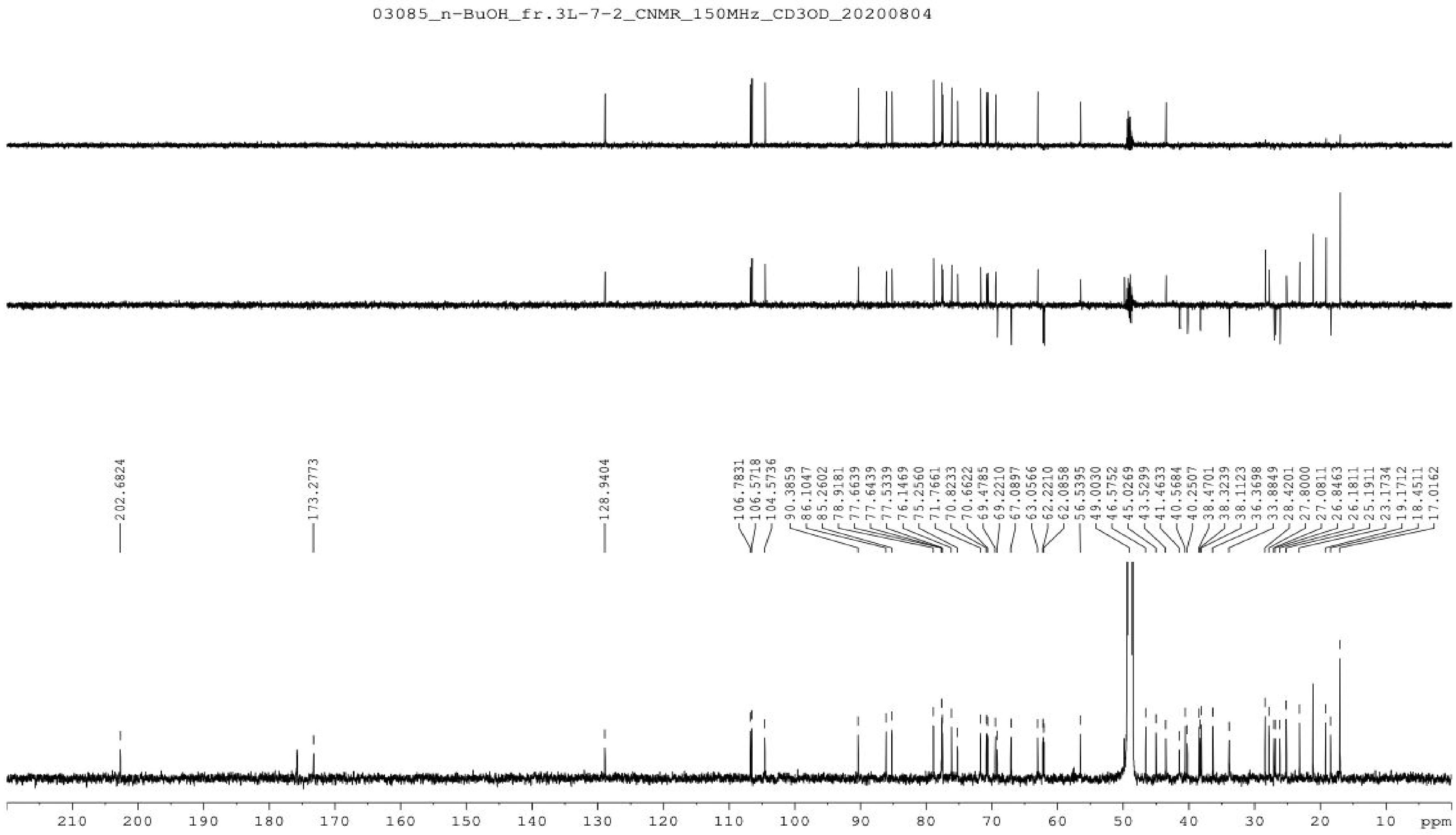

Fig. S20. ^{13}C NMR spectrum of **2** (BBD, bot.; DEPT-135, mid.; DEPT-90, top) (CD_3OD, 150 MHz)

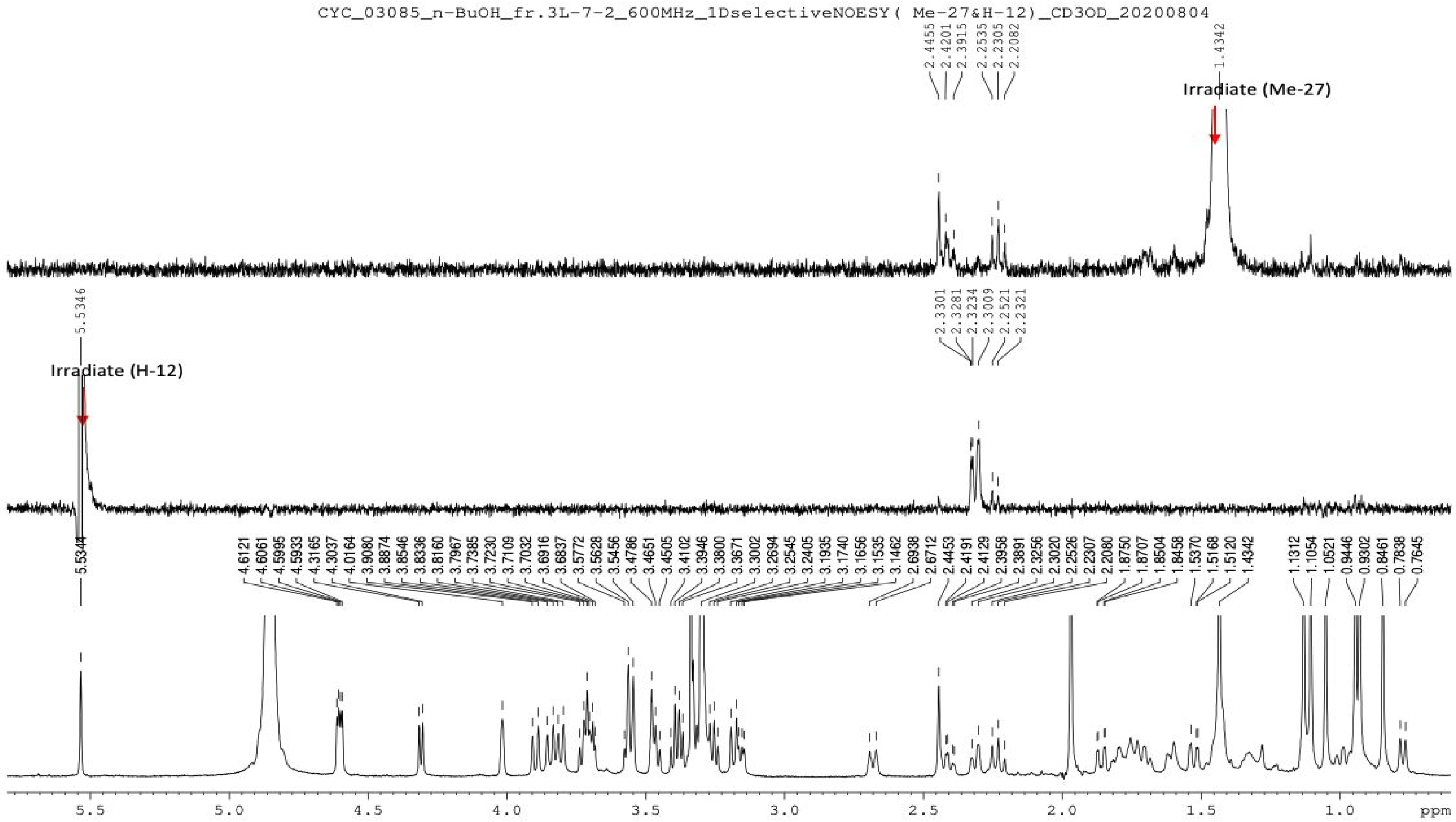

Fig. S21. 1D-NOESY spectrum of **2** (CD_3OD, 600 MHz) (Me-27&H-12)

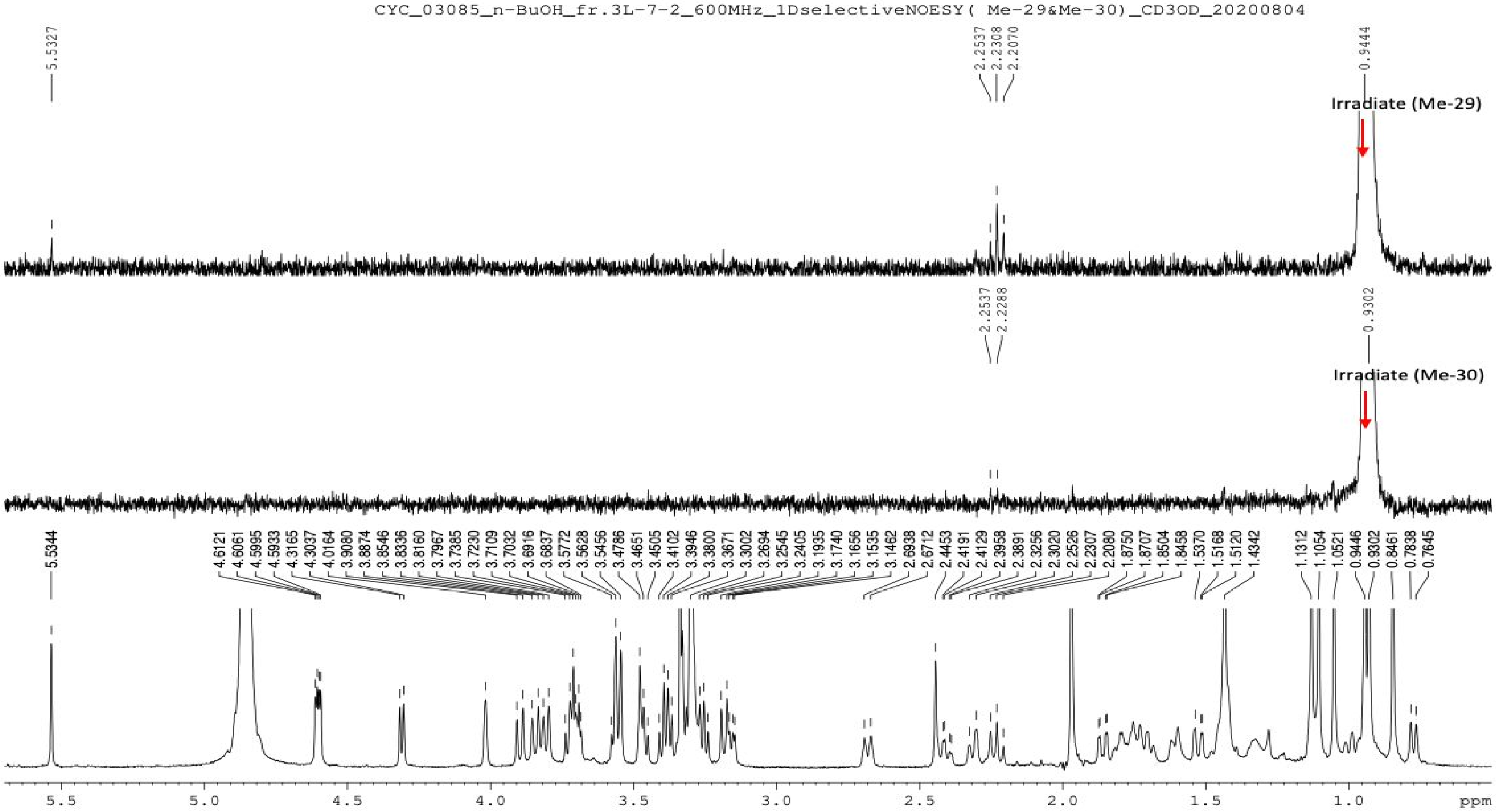

Fig. S22. 1D-NOESY spectrum of **2** (CD_3OD, 600 MHz) (Me-29&Me-30)

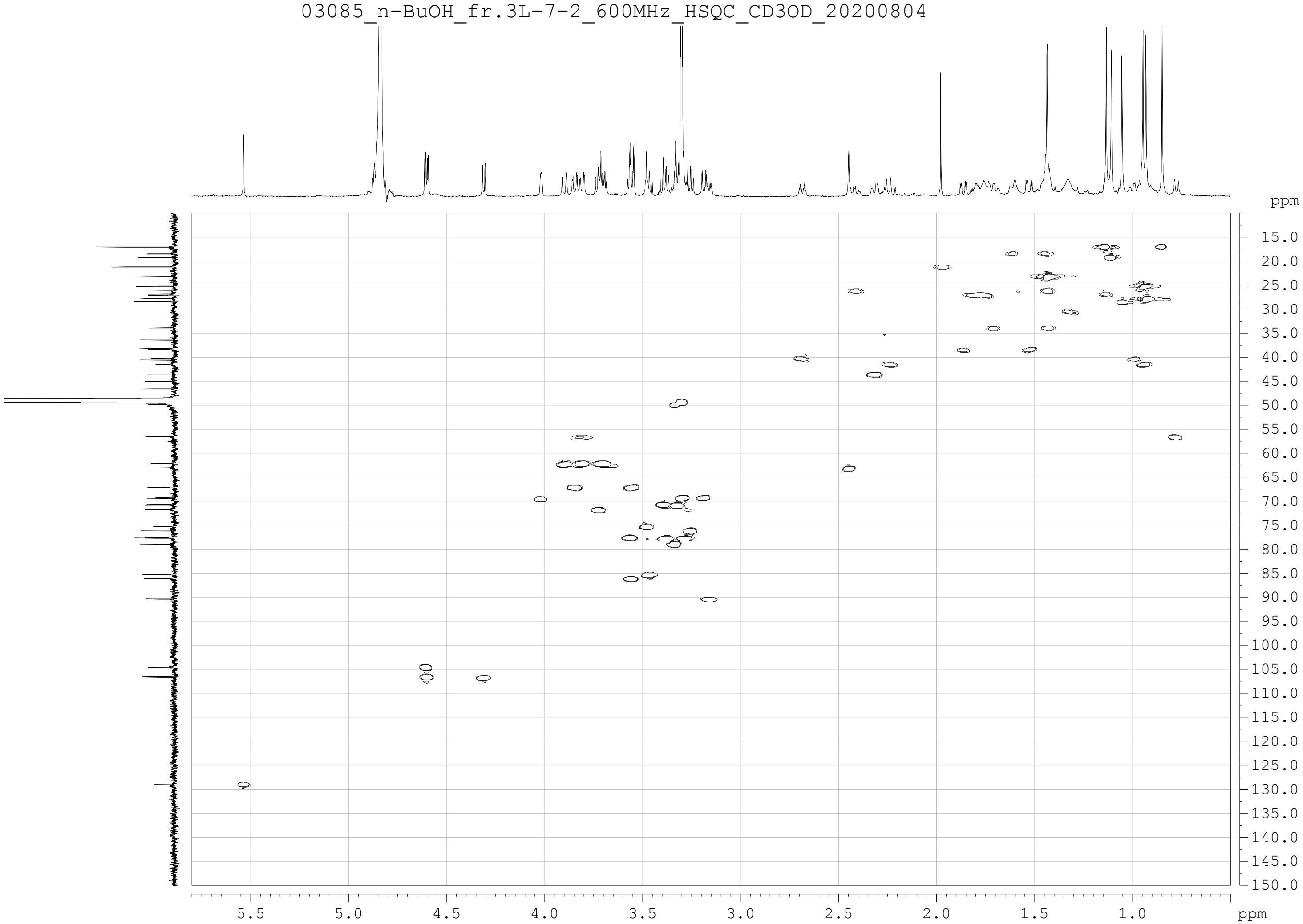

Fig. S23. HSQC spectrum of **2** (CD_3OD, 600 MHz)

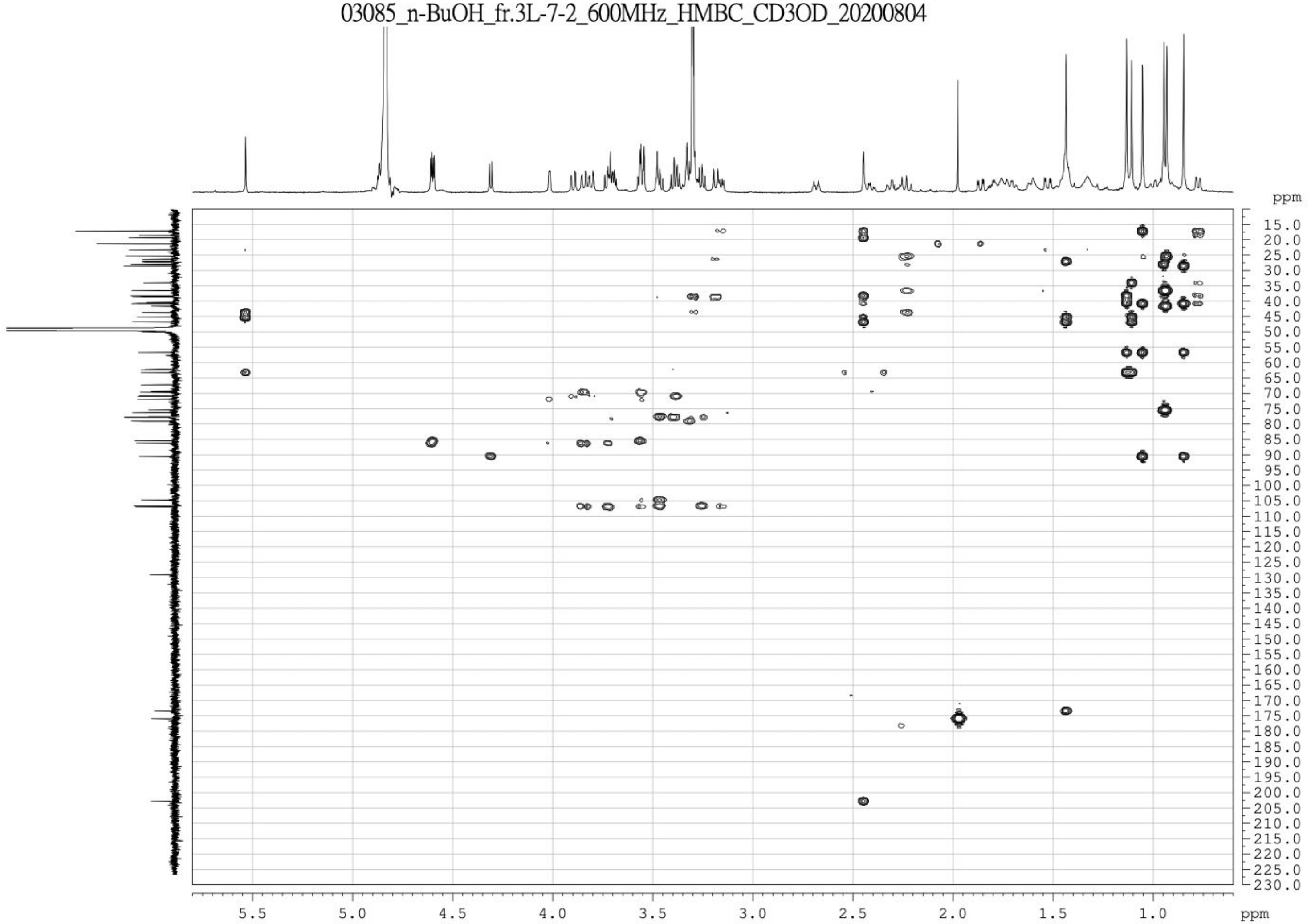

Fig. S24. HMBC spectrum of **2** (CD_3OD, 600 MHz)

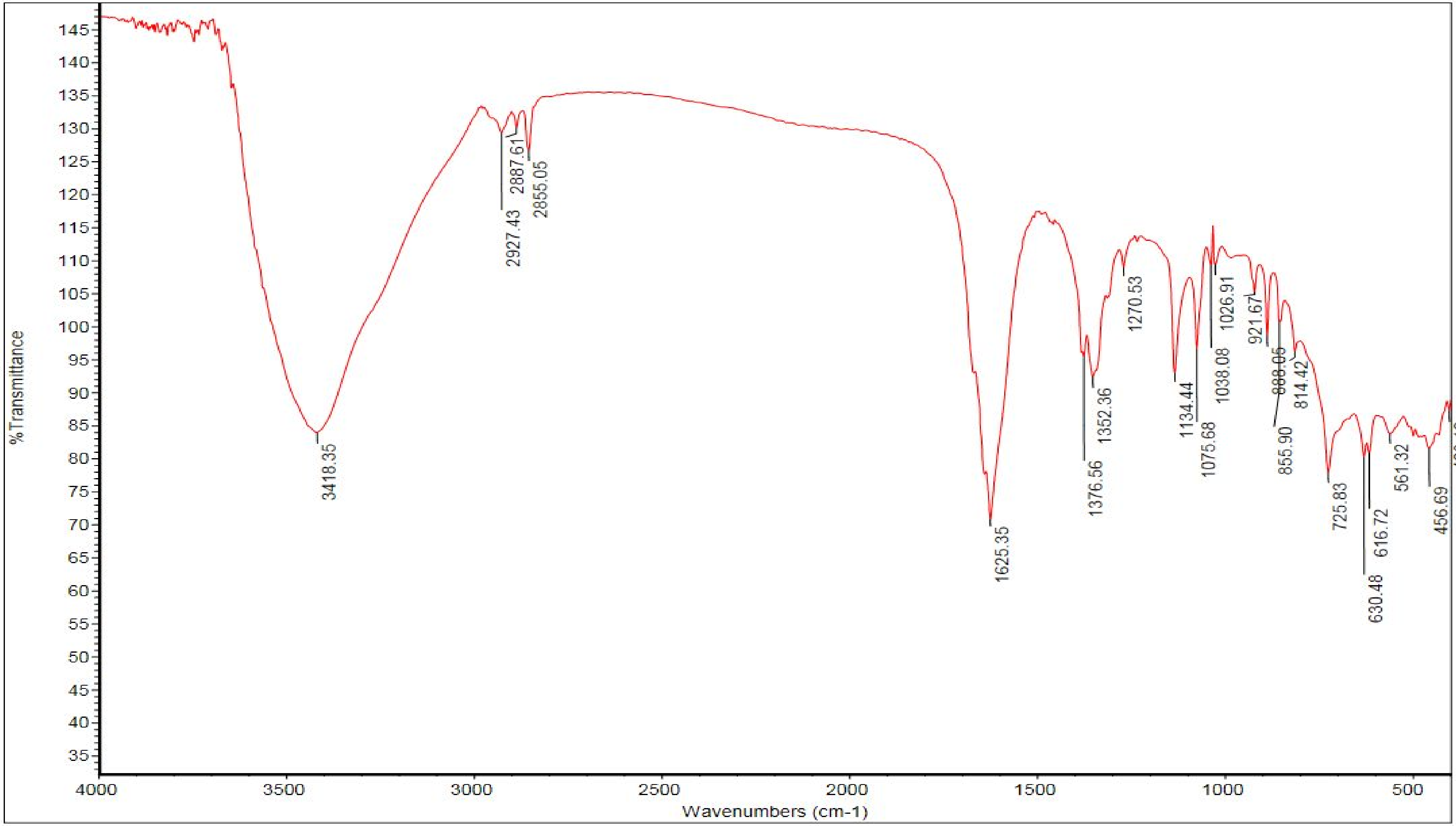

Fig. S25. IR spectrum of **2**

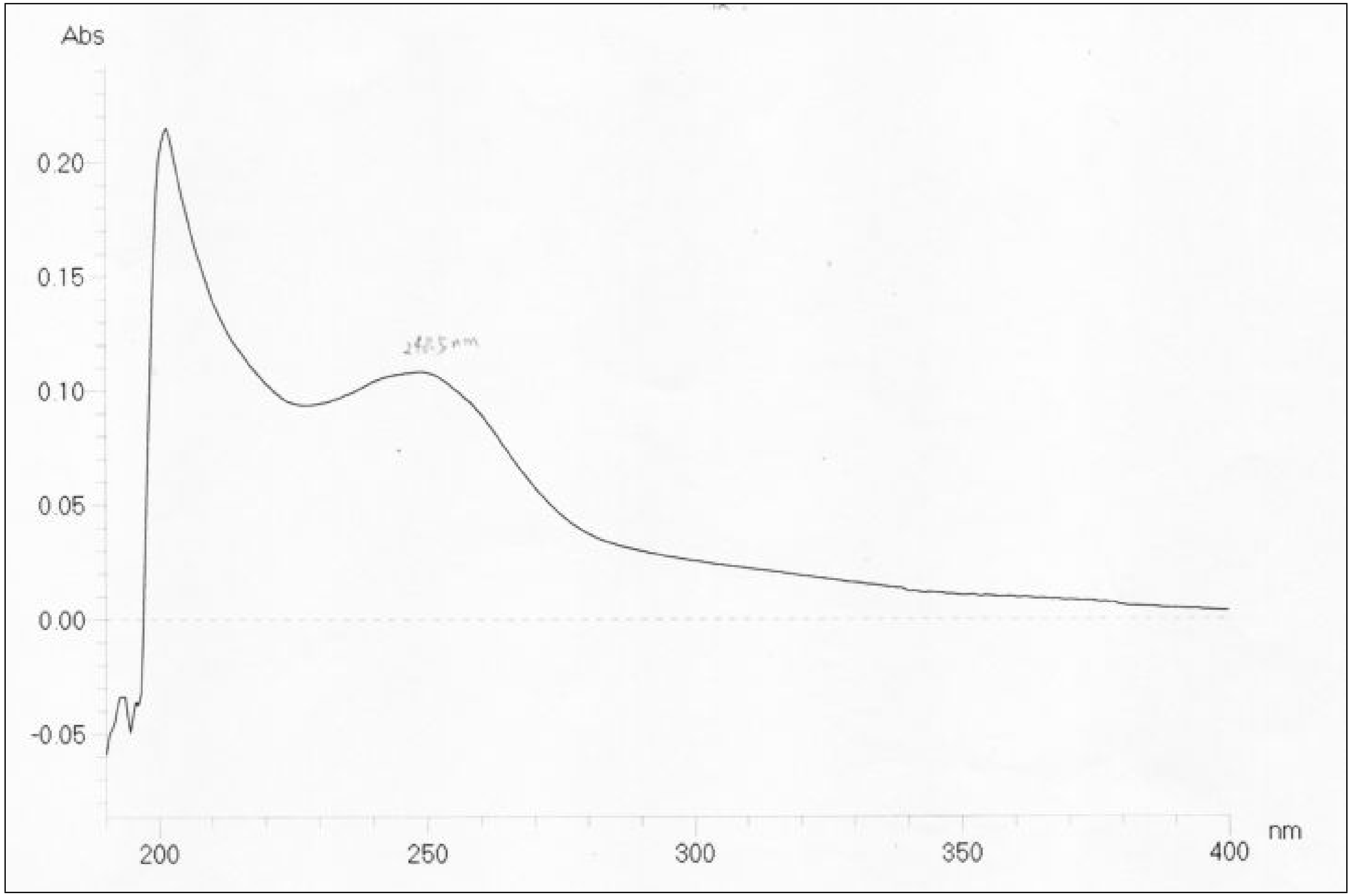

Fig. S26. UV spectrum of **2**

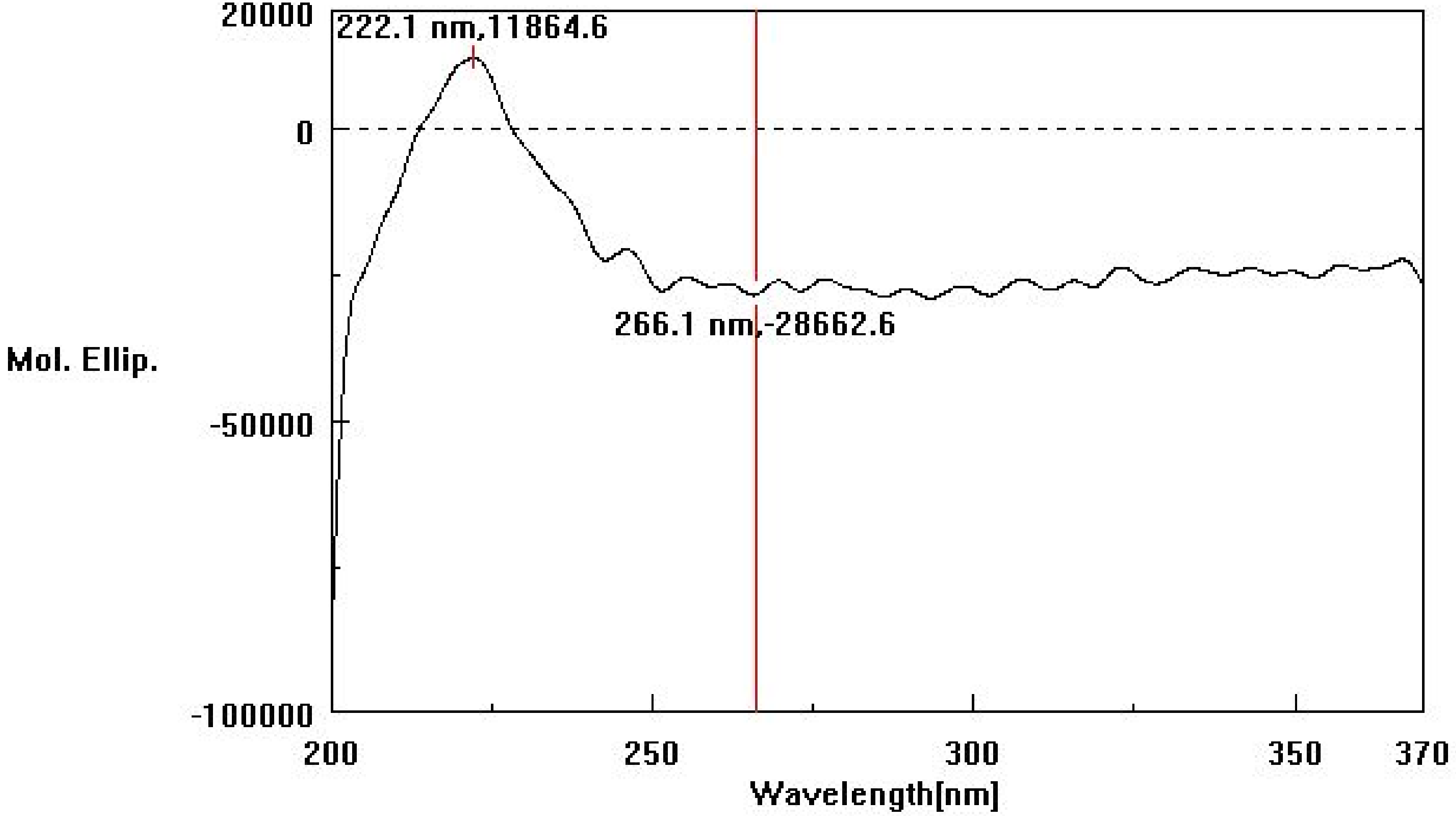

Fig. S27. CD spectrum of **2**

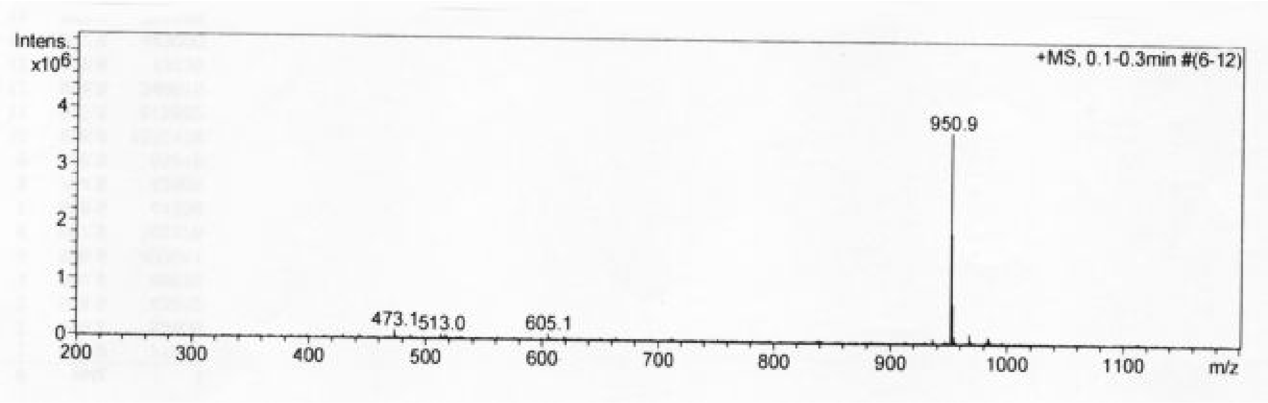

Fig. S28. MS (+) spectrum of **2** (positive mode)

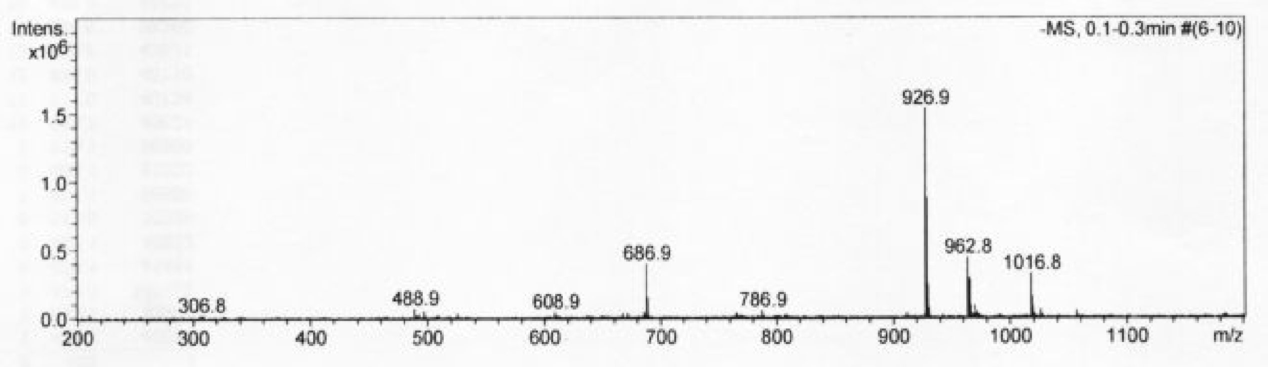

Fig. S29. MS spectrum of **2** (negative mode)

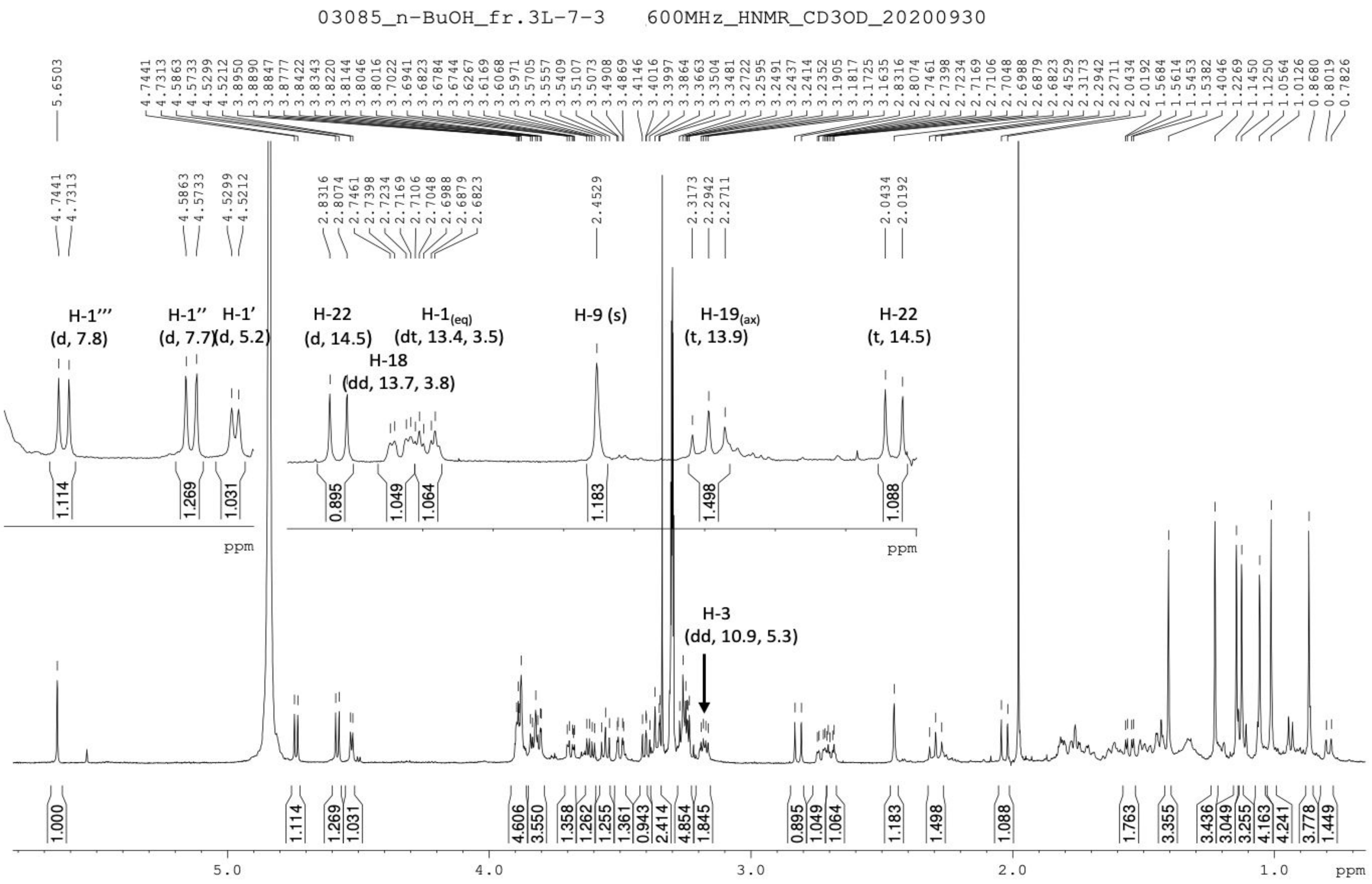

Fig. S30. ^{1}H NMR spectrum of **3** (CD_3OD, 600 MHz)

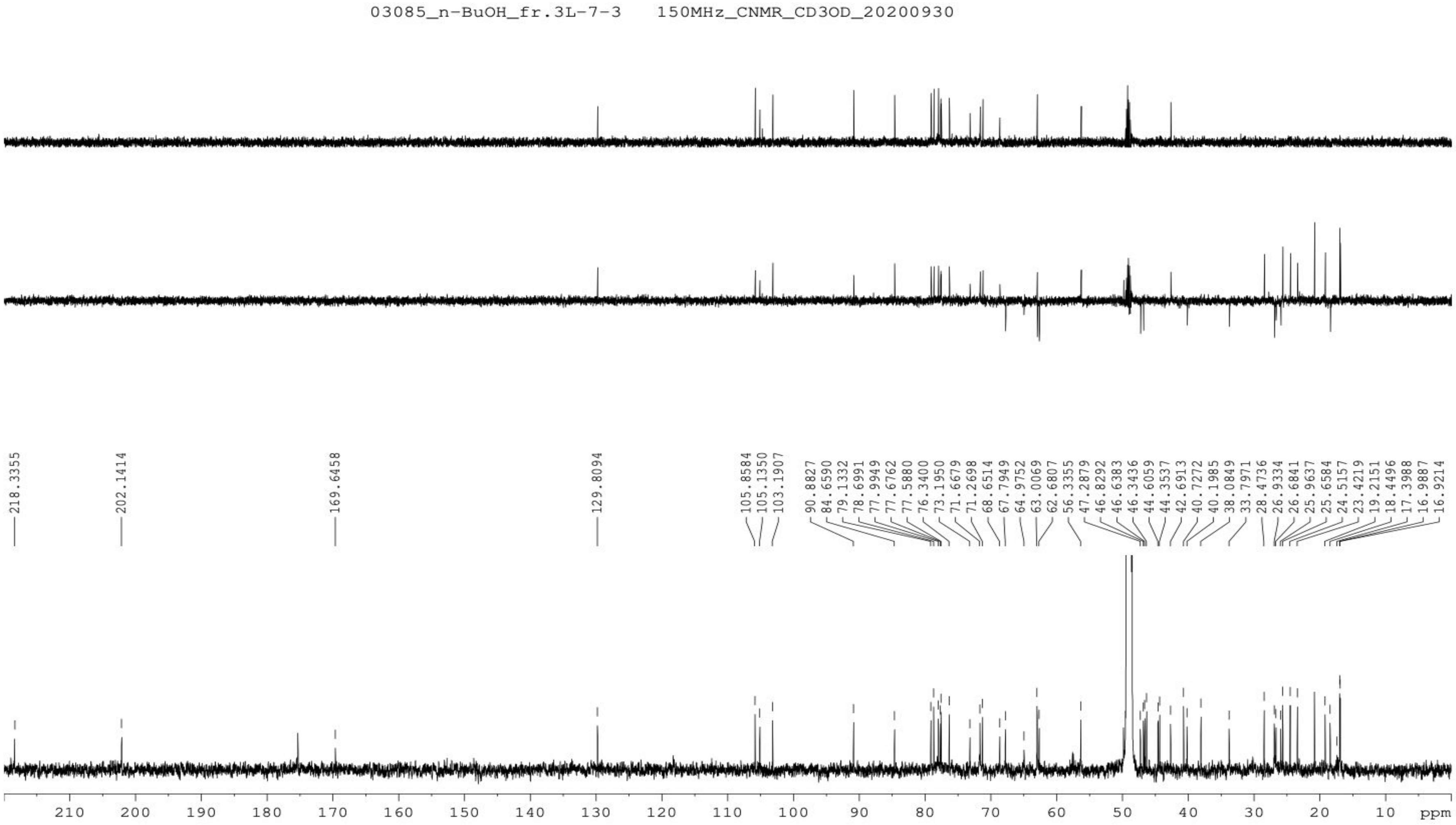

Fig. S31. ^{13}C NMR spectrum of **3** (BBD, bot.; DEPT-135, mid.; DEPT-90, top) (CD_3OD, 150 MHz)

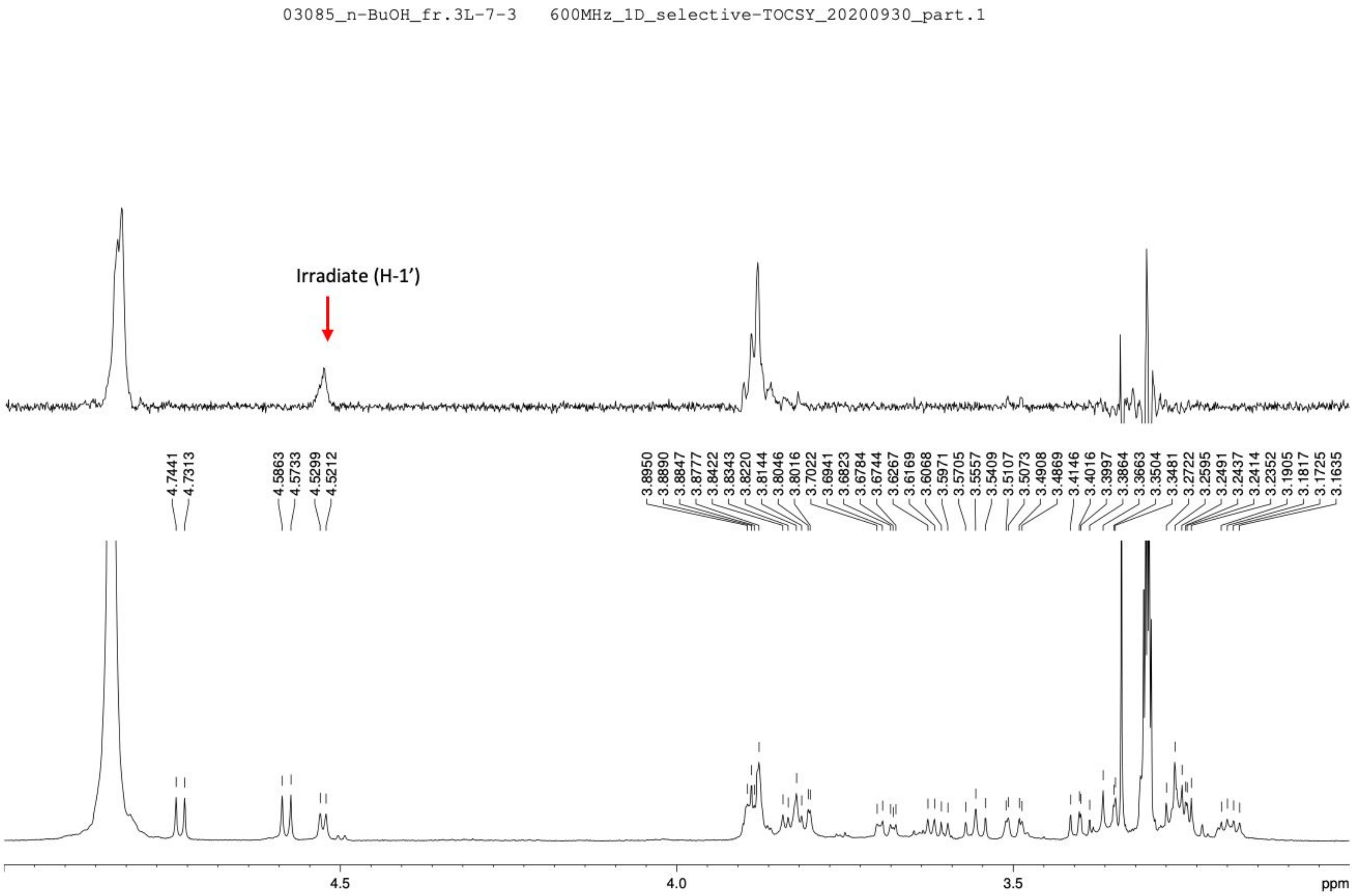

Fig. S32. 1D-TOCSY spectrum of **3** (CD_3OD, 600 MHz) (H-1′)

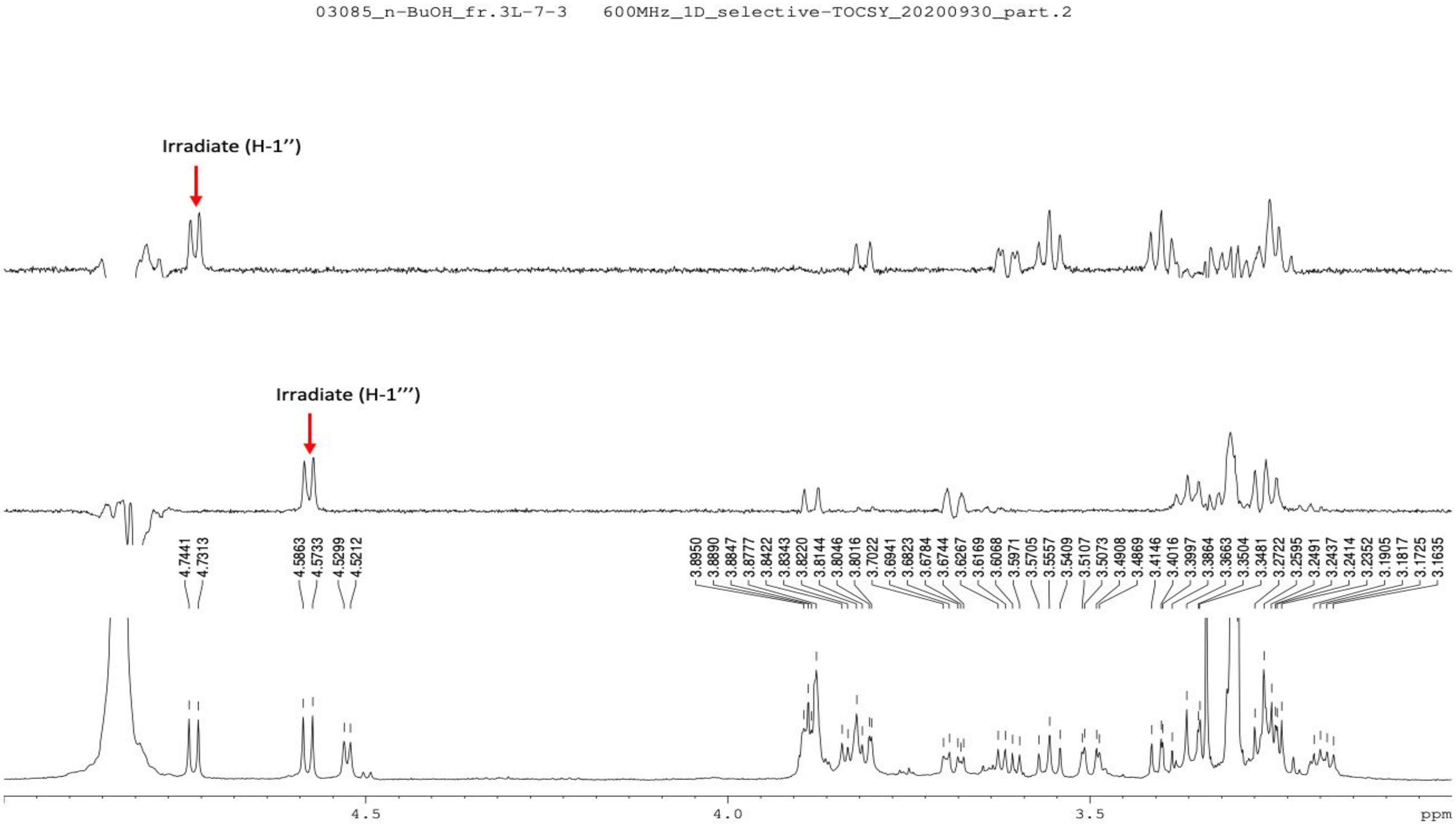

Fig. S33. 1D-TOCSY spectrum of **3** (CD_3OD, 600 MHz) (H-1″ &1‴)

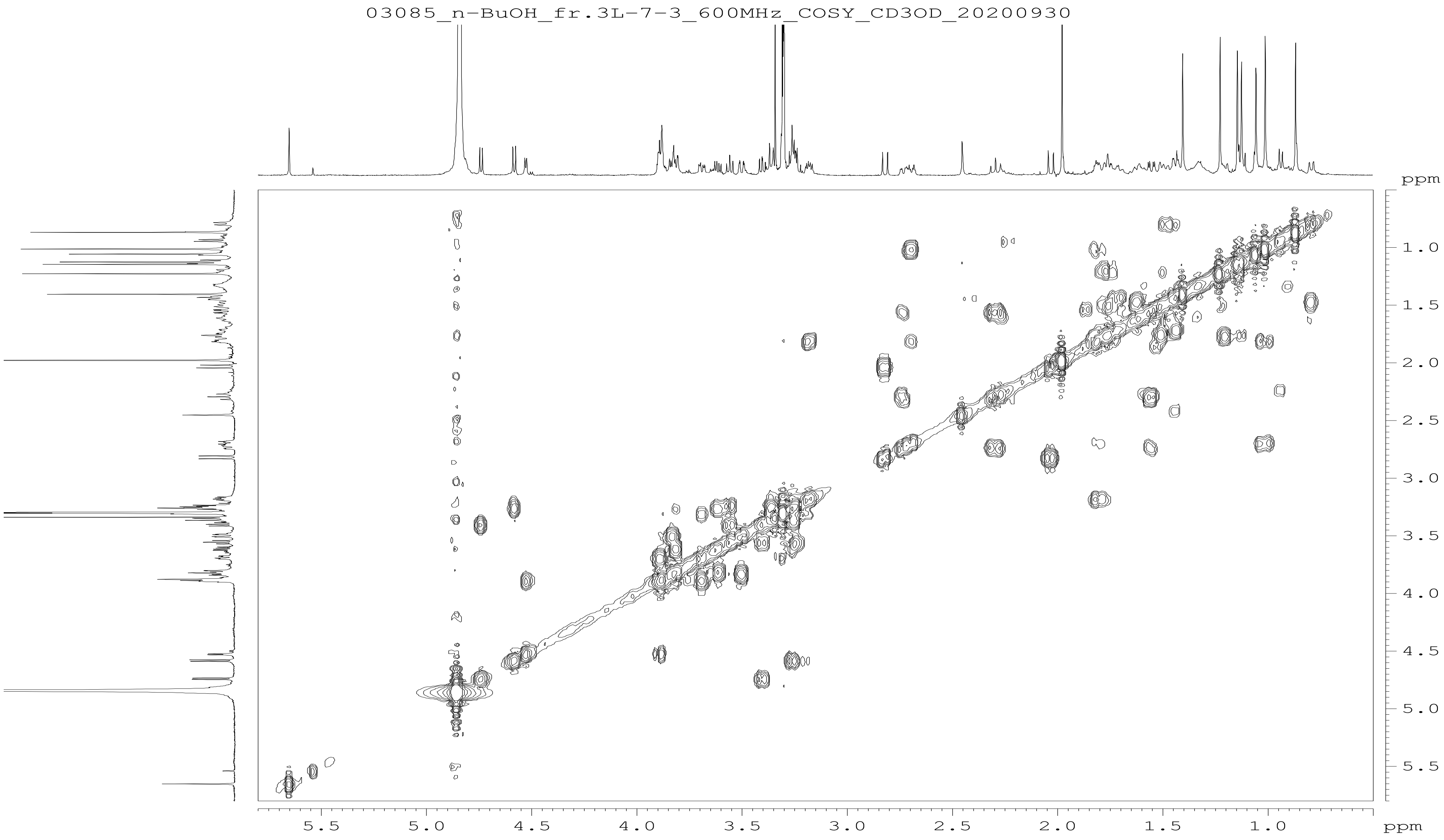

Fig. S34. COSY spectrum of **3** (CD_3OD, 600 MHz).

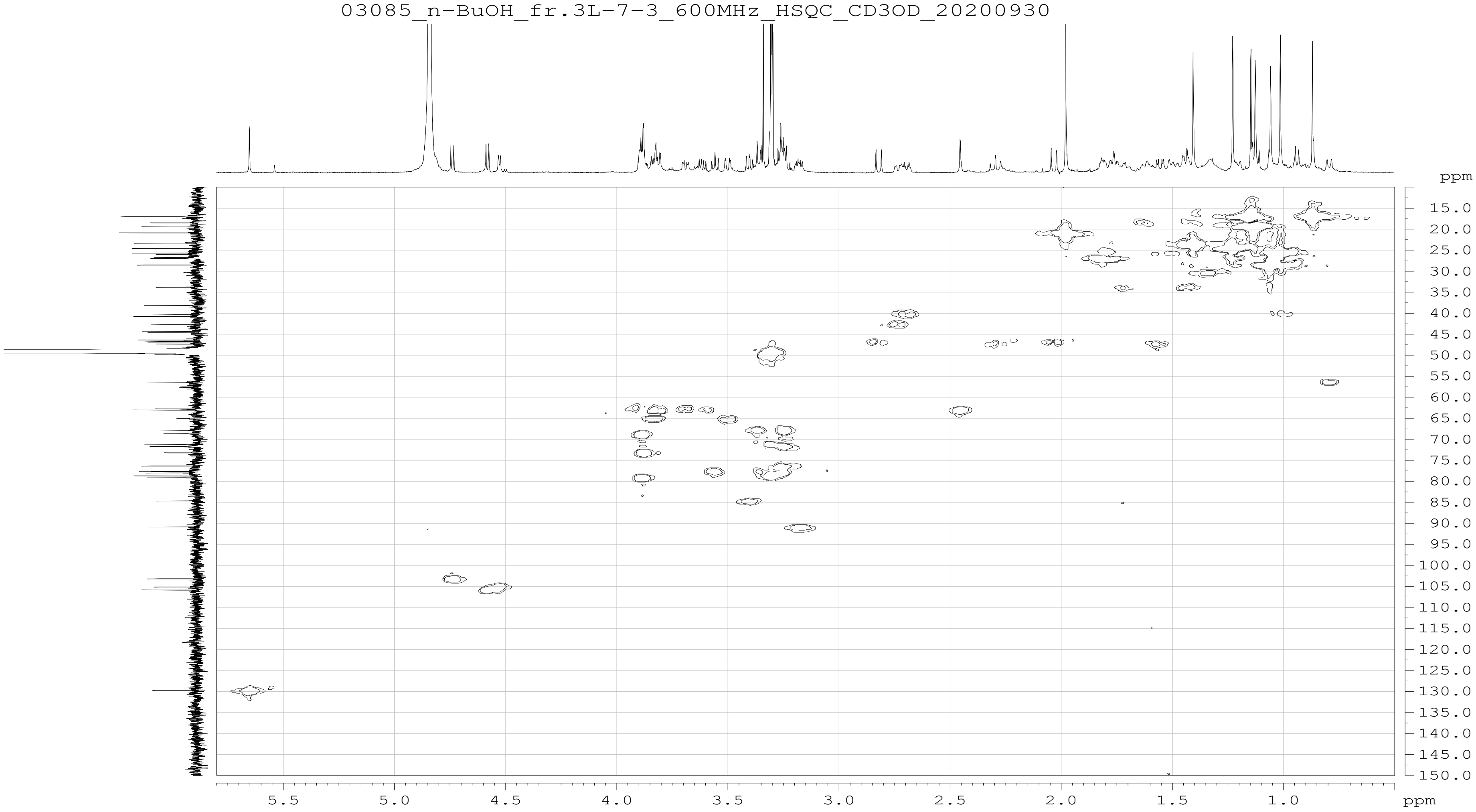

Fig. S35. HSQC spectrum of **3** (CD_3OD, 600 MHz)

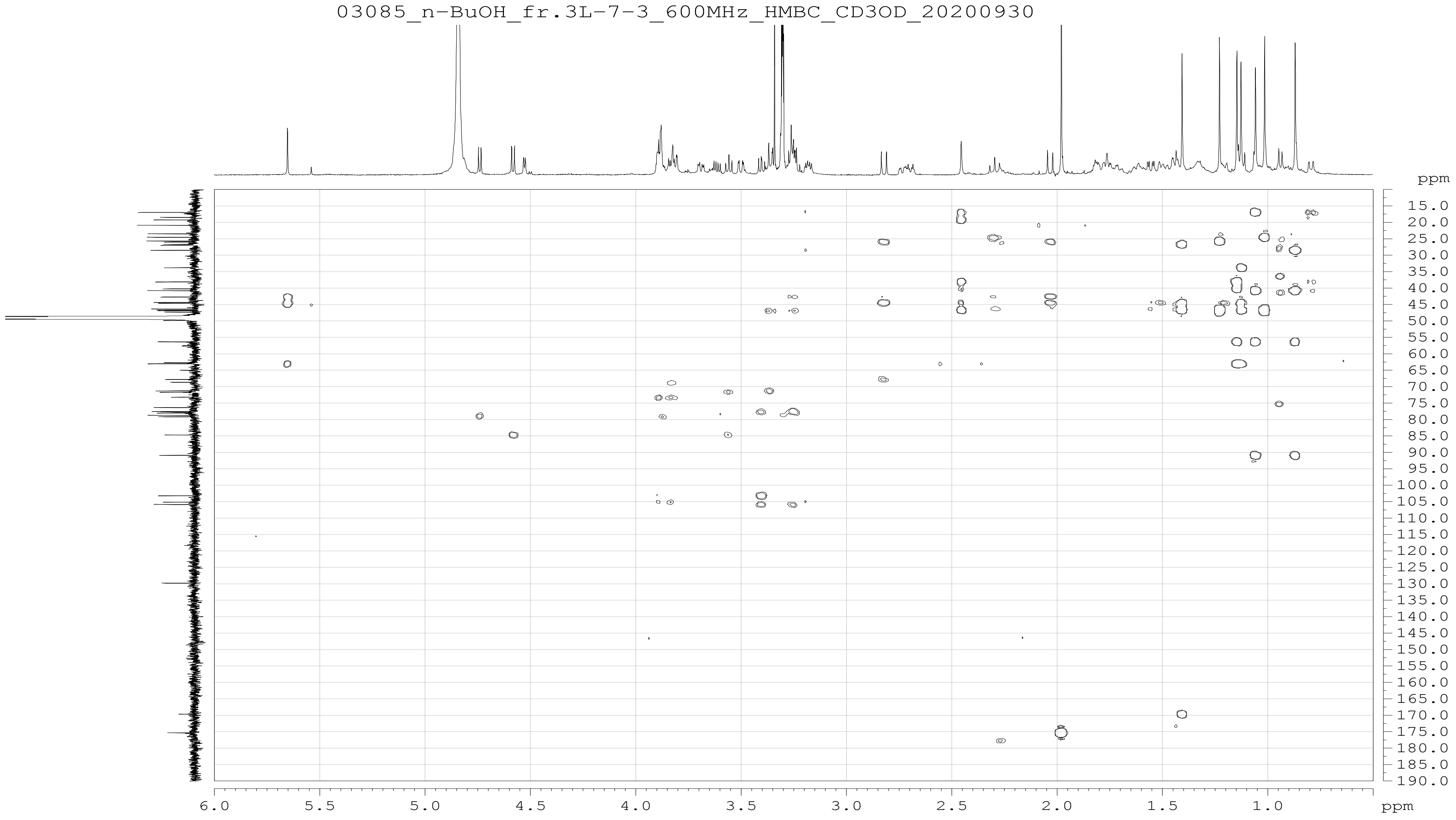

Fig. S36. HMBC spectrum of **3** (CD_3OD, 600 MHz)

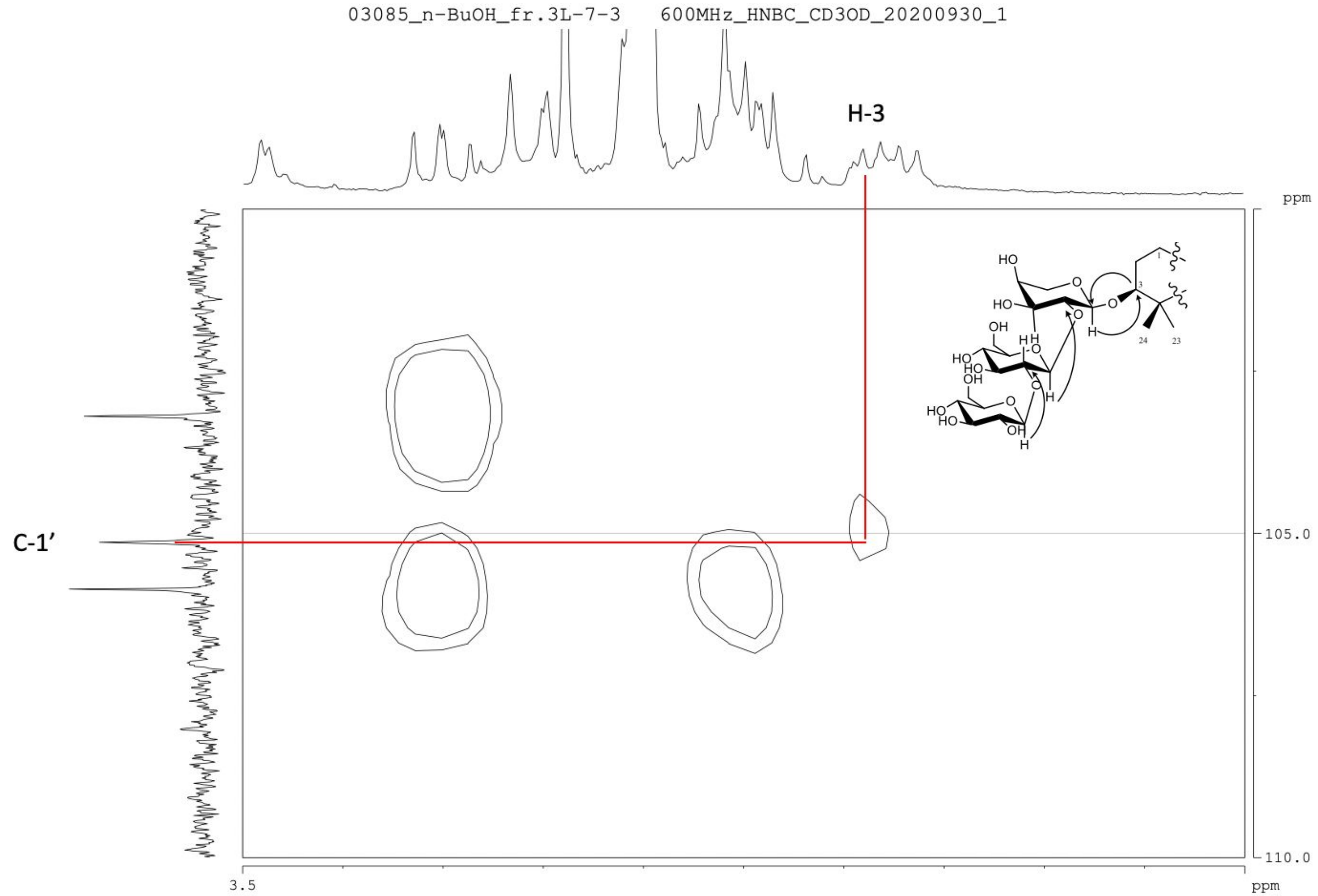

Fig. S37. HMBC spectrum of **3** (CD_3OD, 600 MHz) (glycosidic linkages-1)

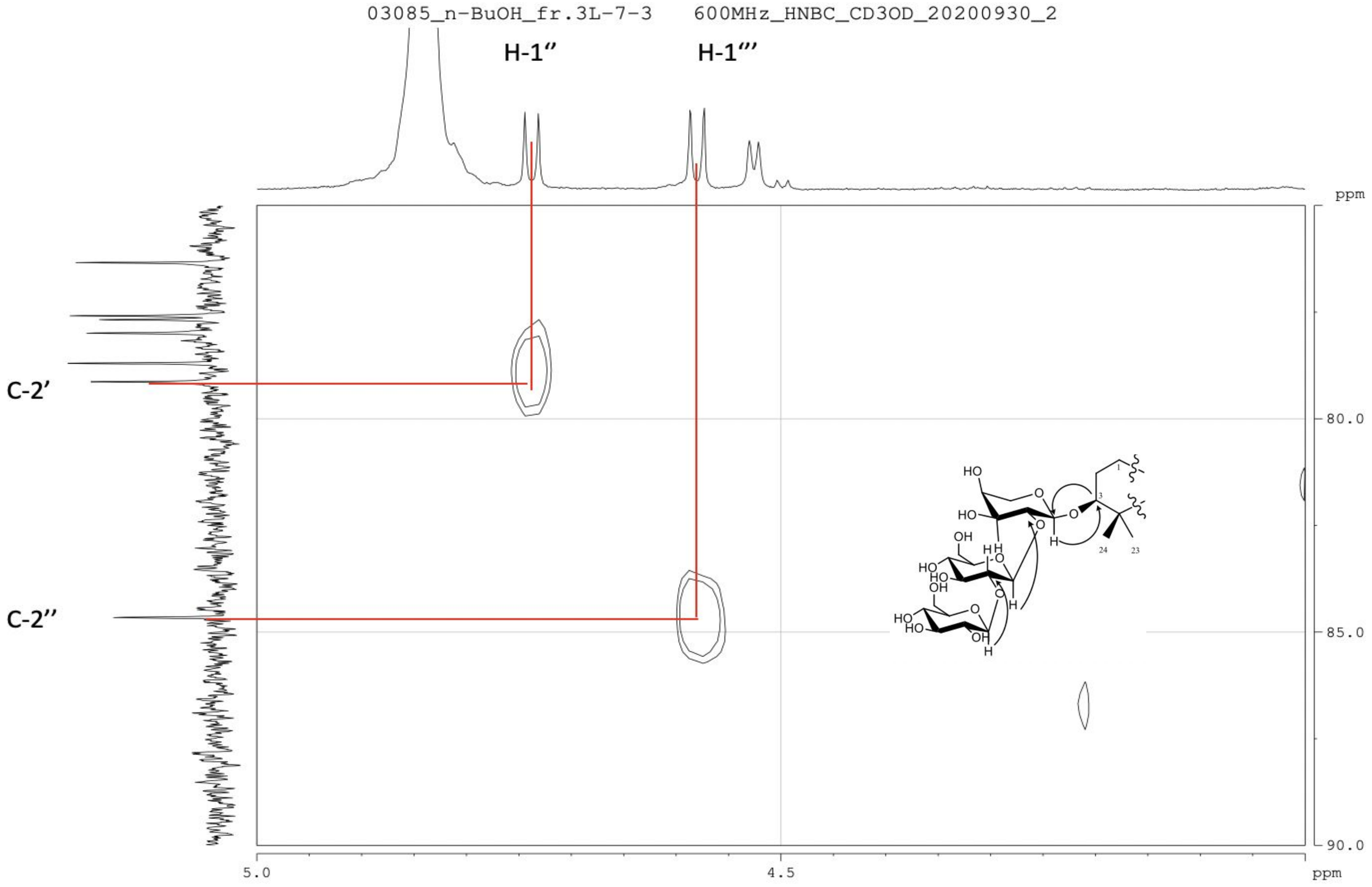

Fig. S38. HMBC spectrum of **3** (CD_3OD, 600 MHz) (glycosidic linkages-2)

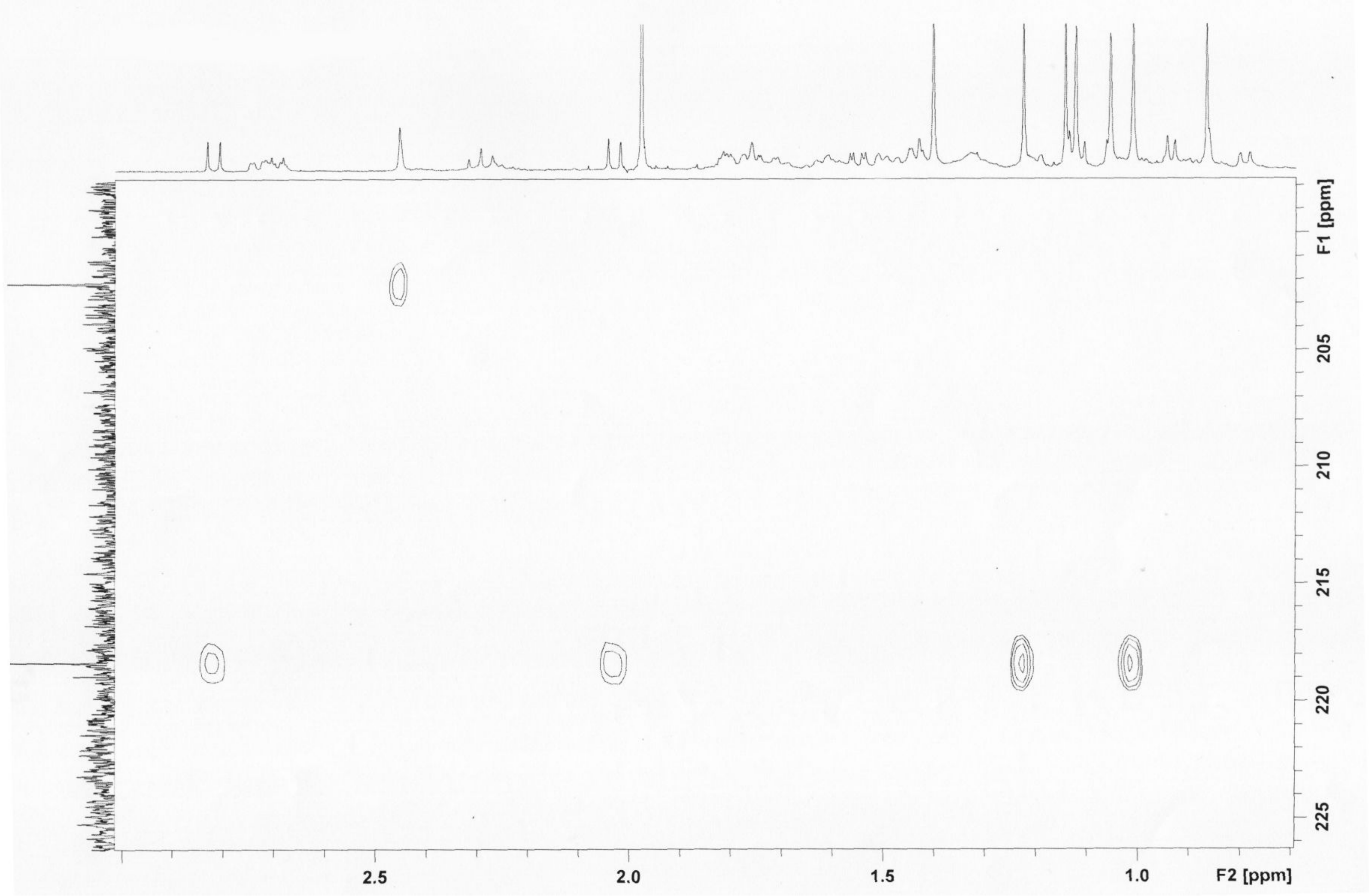

Fig. S39. HMBC spectrum of **3** (CD_3OD, 600 MHz) (Proof of the ketone at C-21)

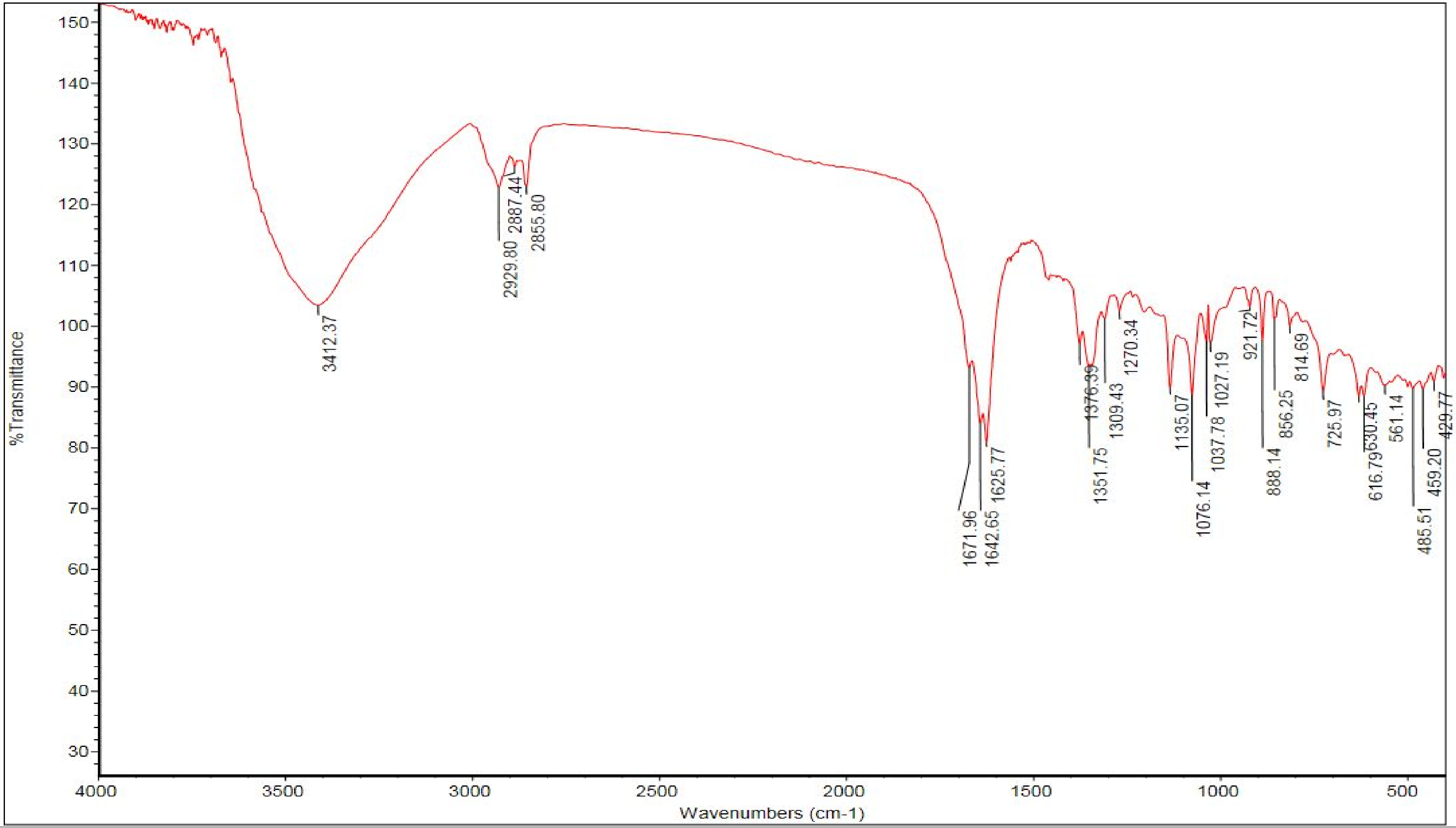

Fig. S40. IR spectrum of **3**

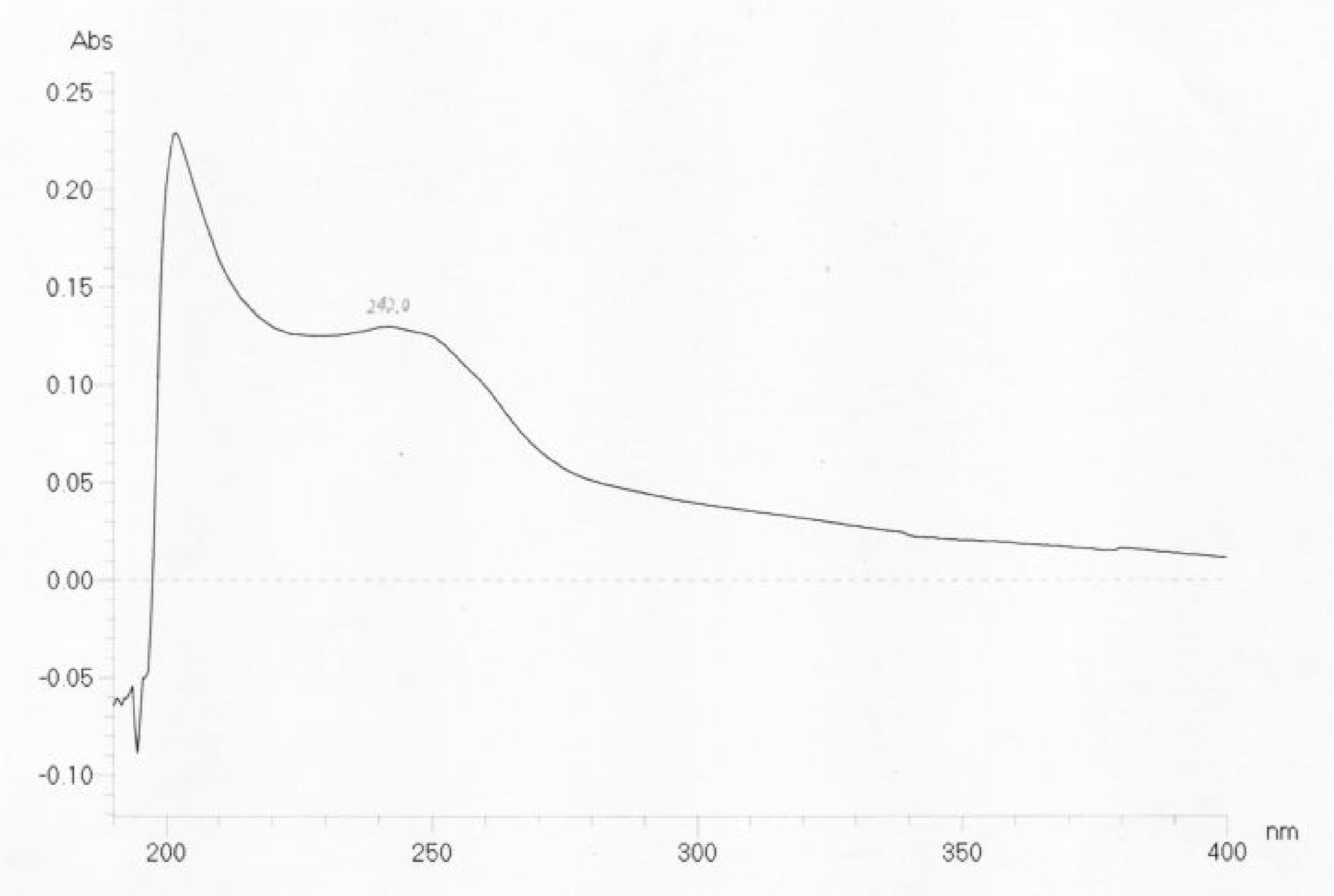

Fig. S40. UV spectrum of **3**

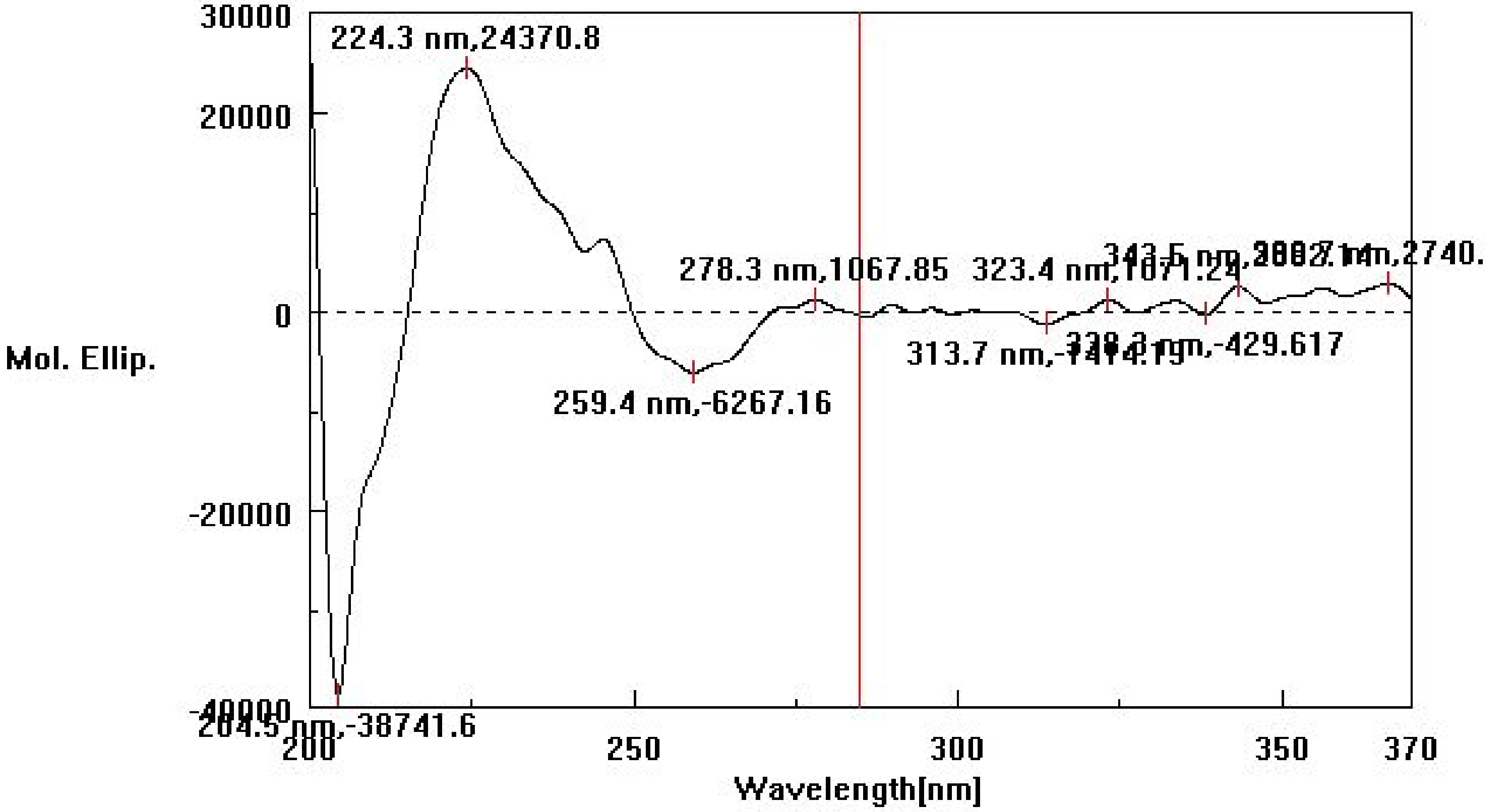

Fig. S42. CD spectrum of **3**

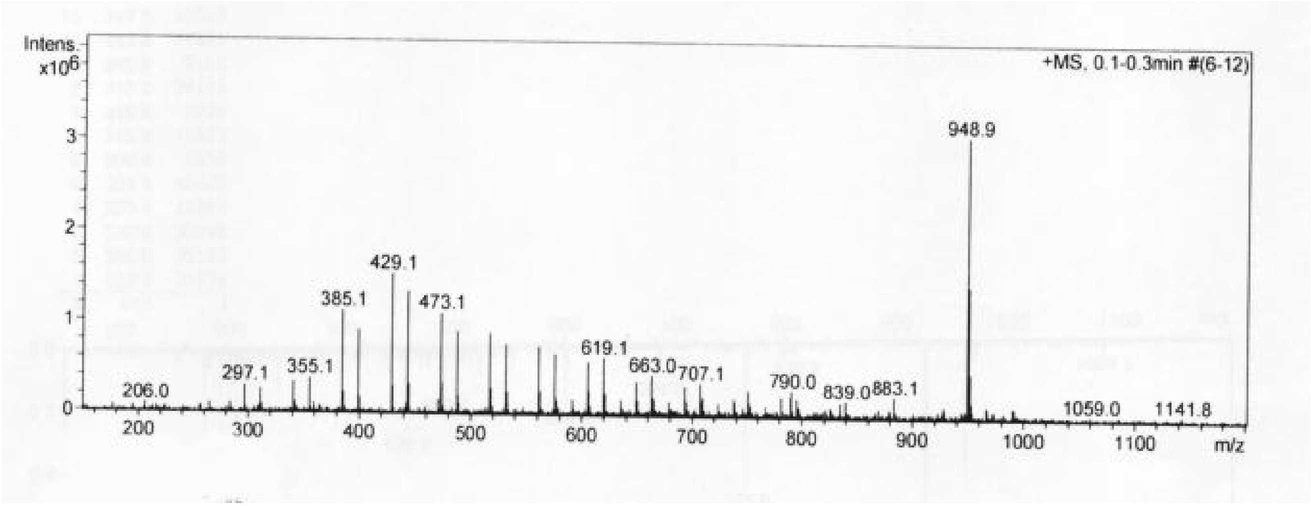

Fig. S43. MS spectrum of **3** (positive mode)

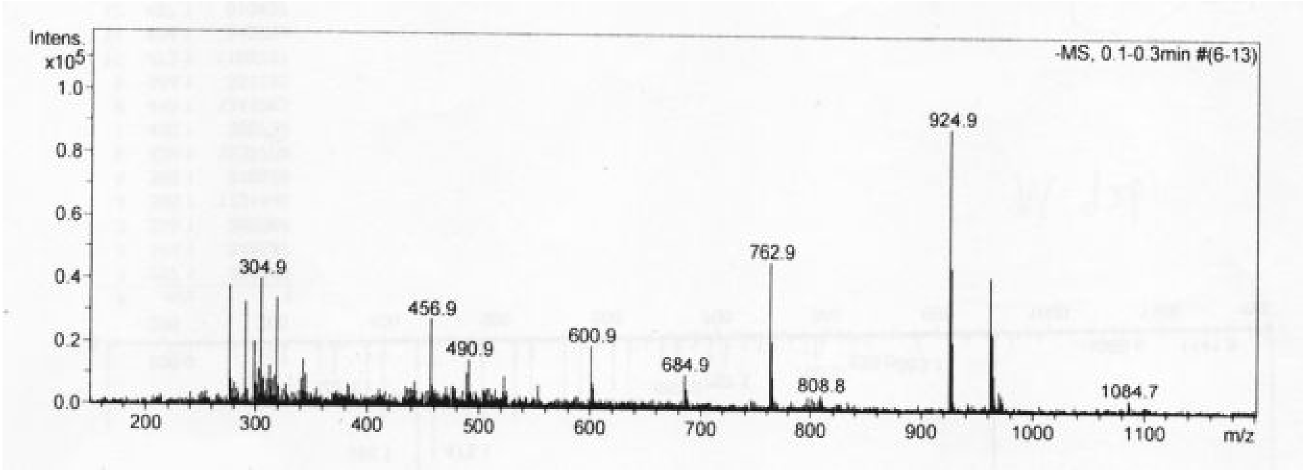

Fig. S44. MS spectrum of **3** (negative mode)

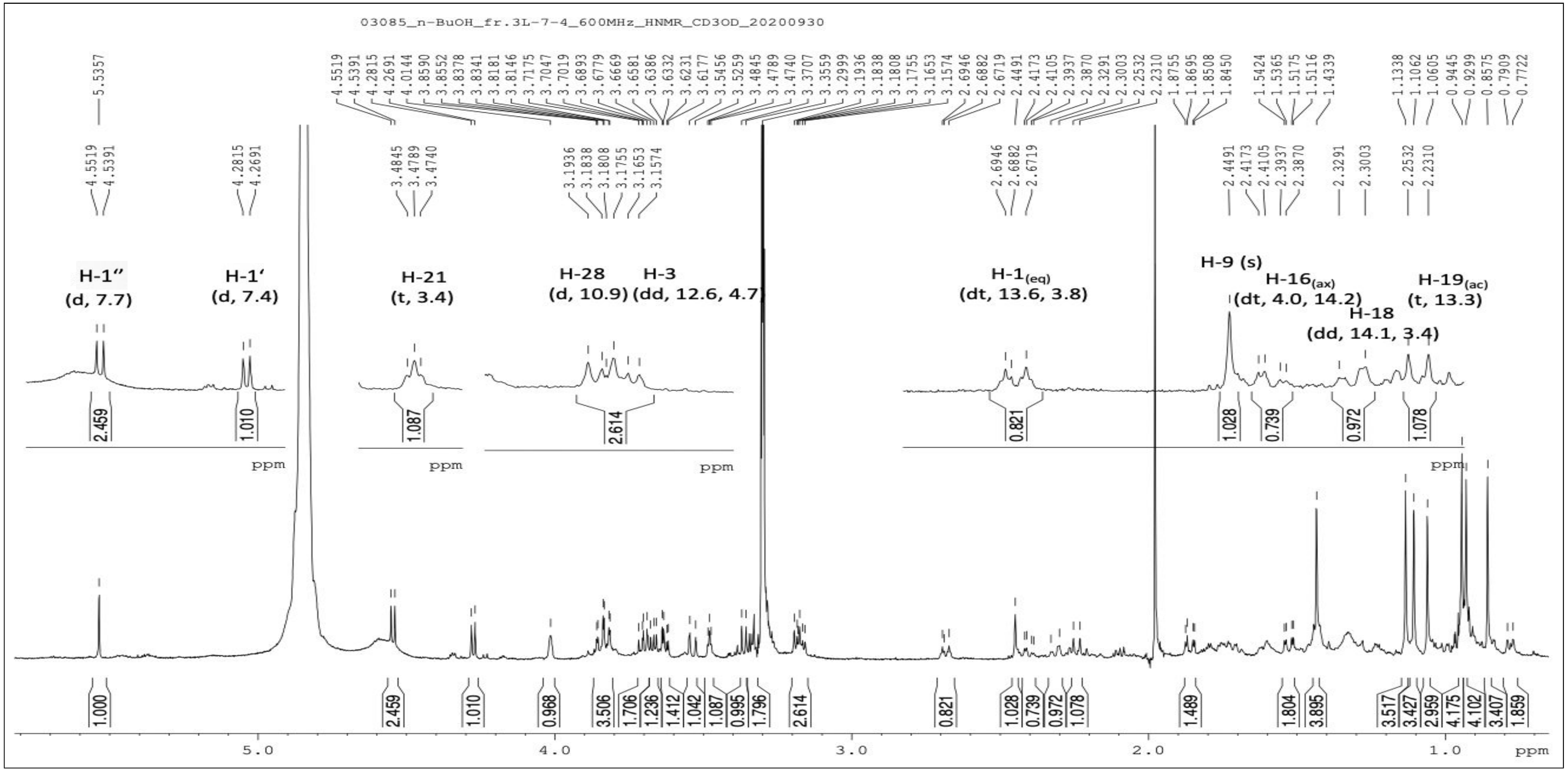

Fig. S45. ^{1}H NMR spectrum of **4** (CD_3OD, 600 MHz)

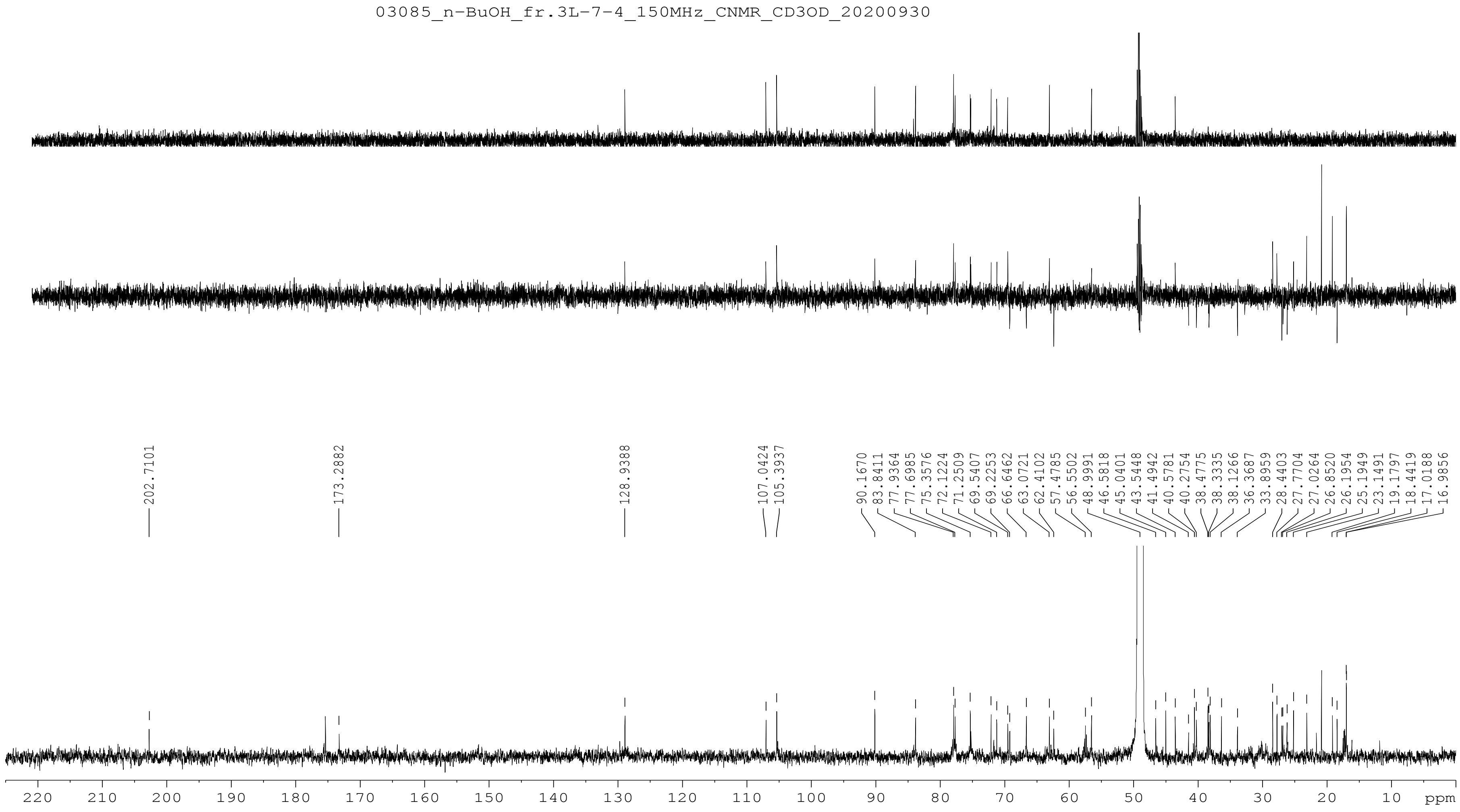

Fig. S46. ^{13}C NMR spectrum of **4** (BBD, bot.; DEPT-135, mid.; DEPT-90, top) (CD_3OD, 150 MHz)

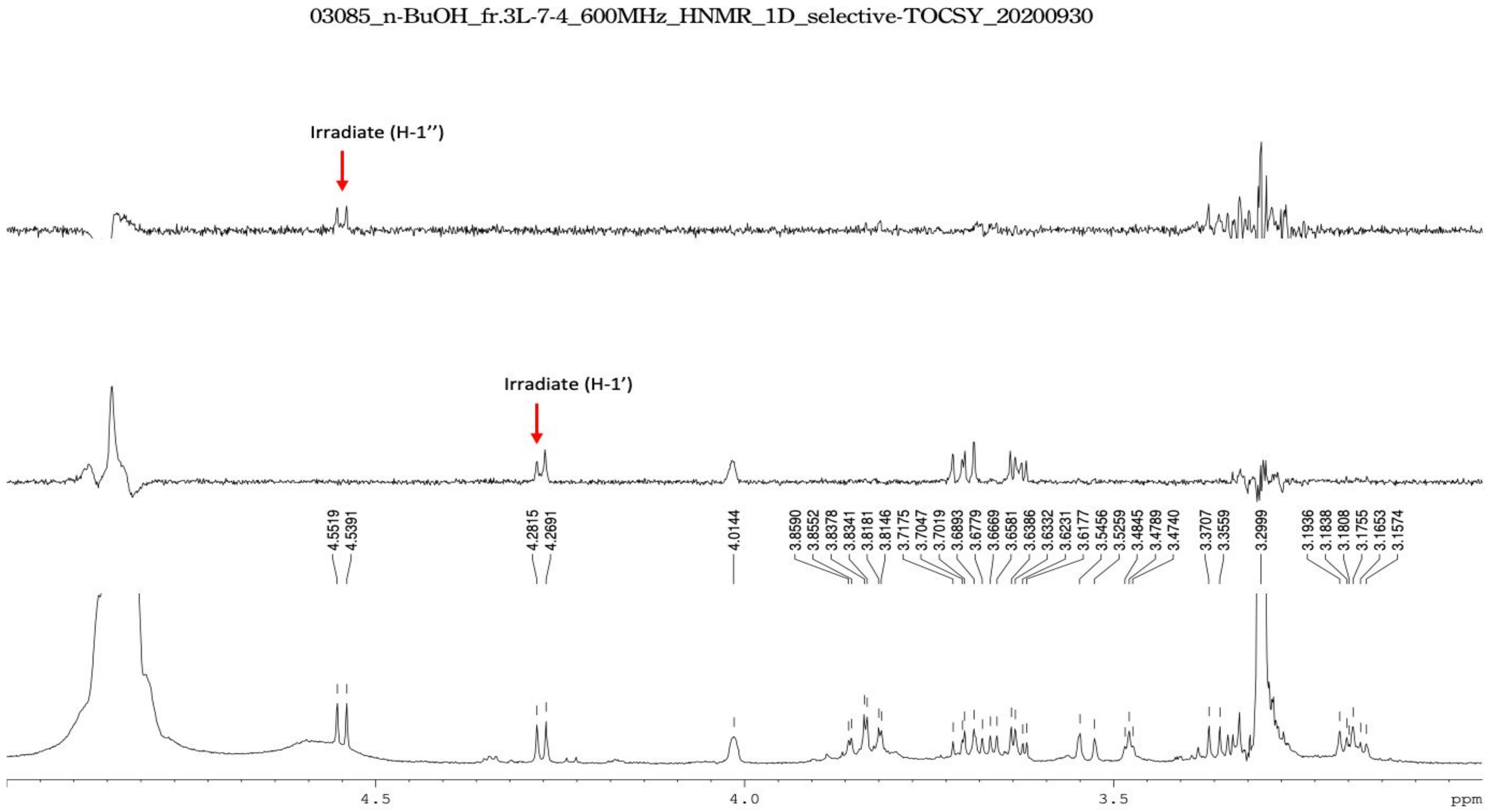

Fig. S47. 1D-TOCSY spectrum of **4** (CD_3OD, 600 MHz) (H-1′ &1″)

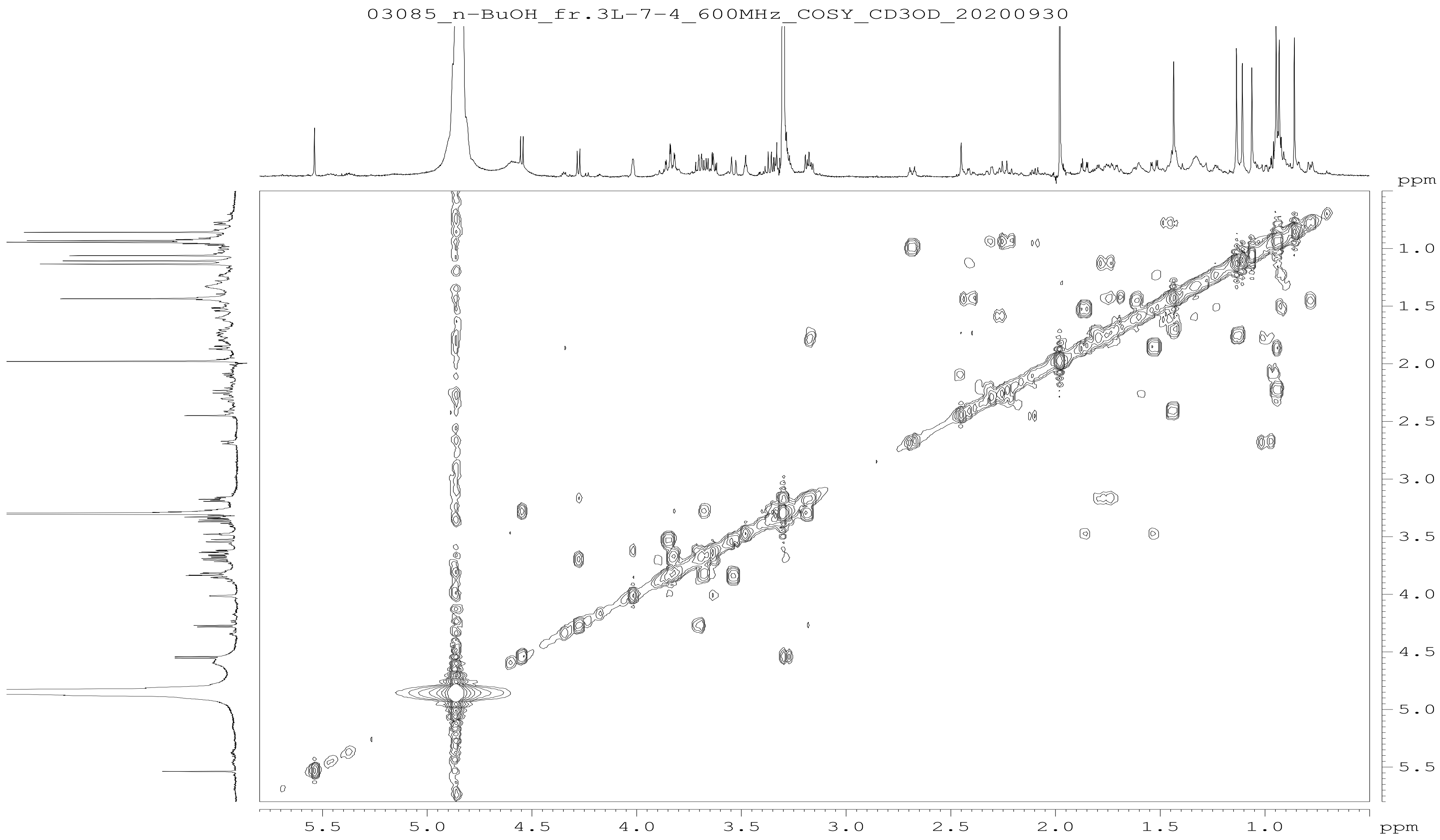

Fig. S48. COSY spectrum of **4** (CD_3OD, 600 MHz)

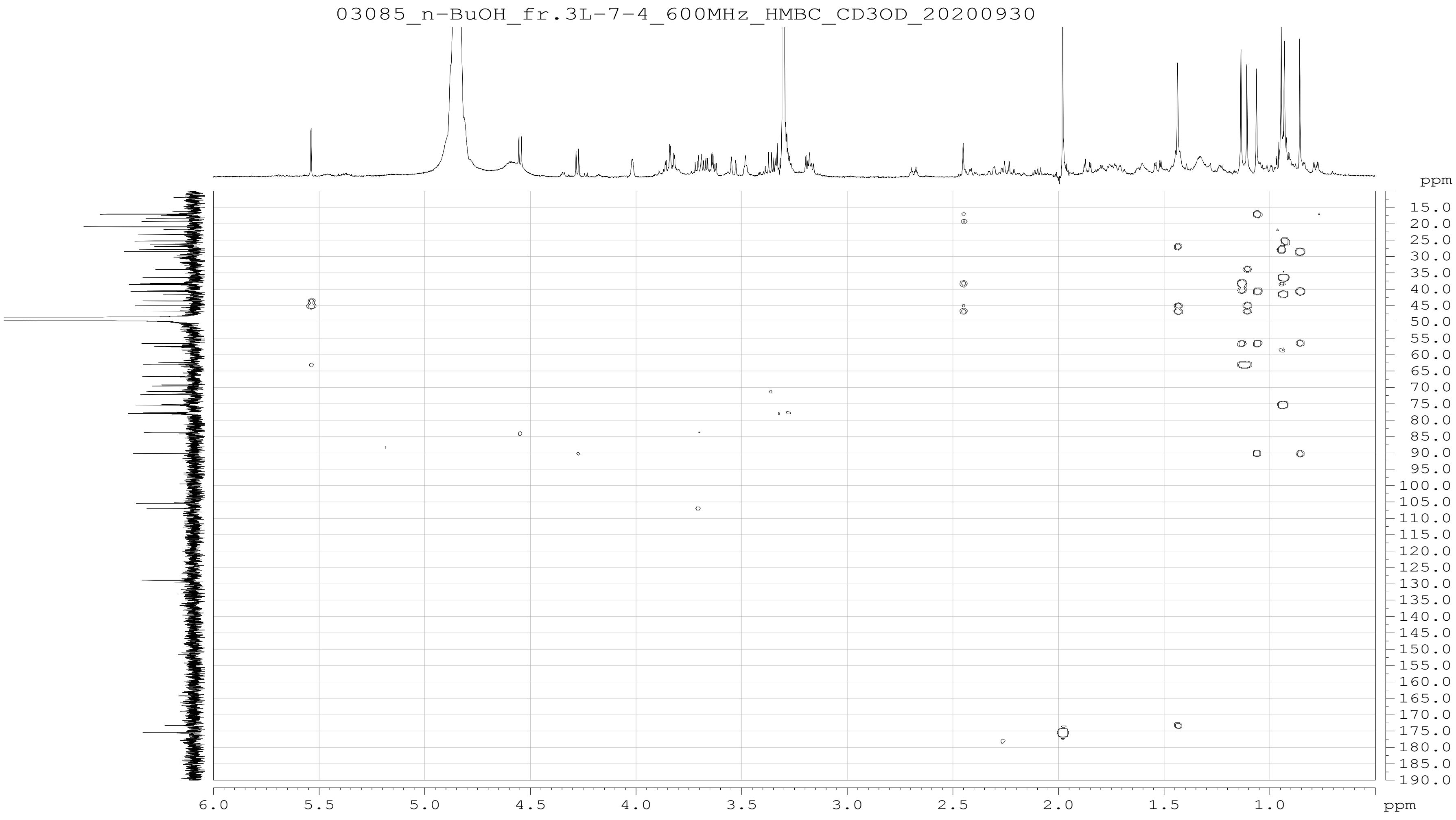

Fig. S49. HMBC spectrum of **4** (CD_3OD, 600 MHz)

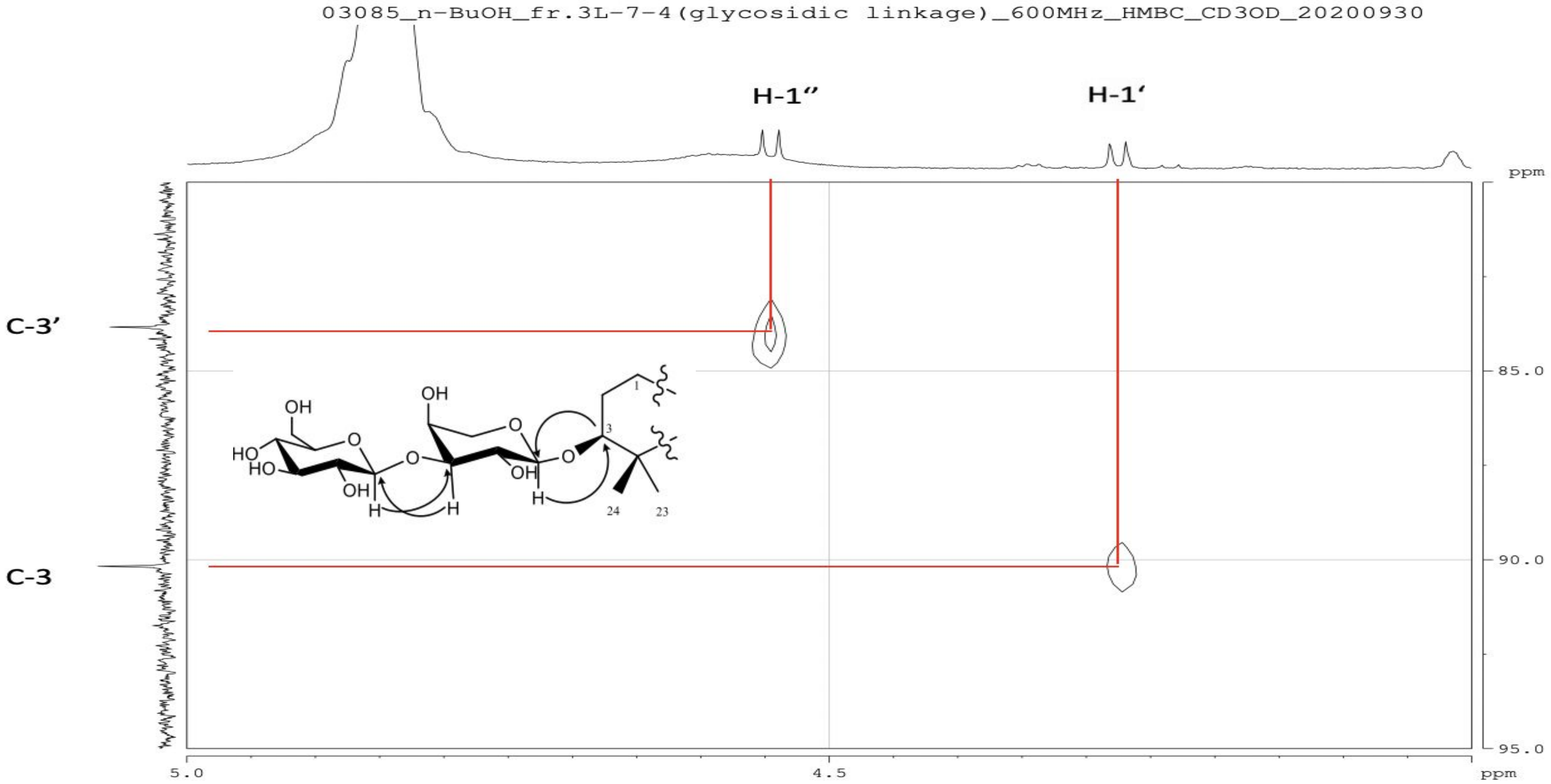

Fig. S50. HMBC spectrum of **4** (CD_3OD, 600 MHz) (glycosidic linkages)

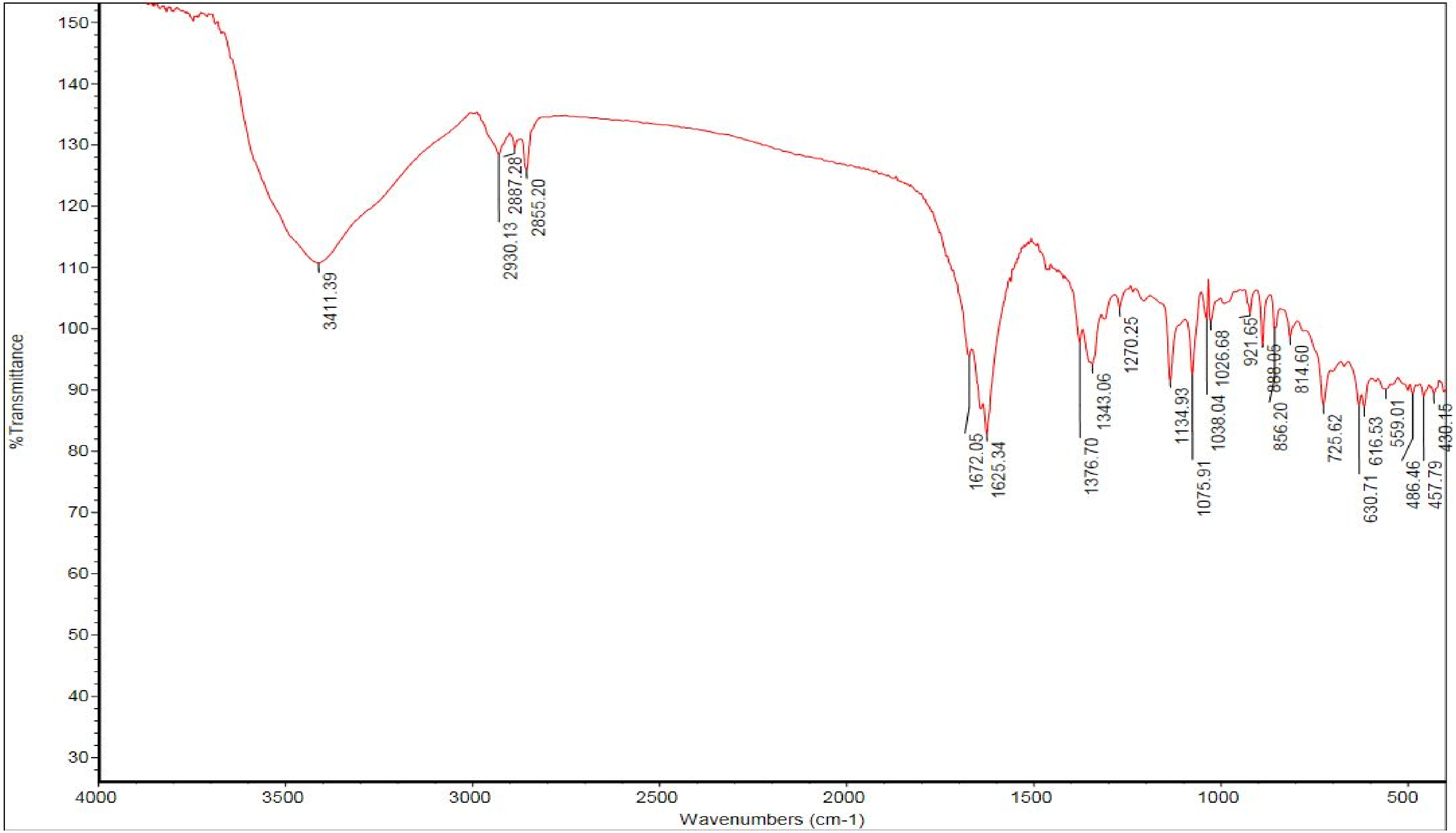

Fig. S51. IR spectrum of **4**

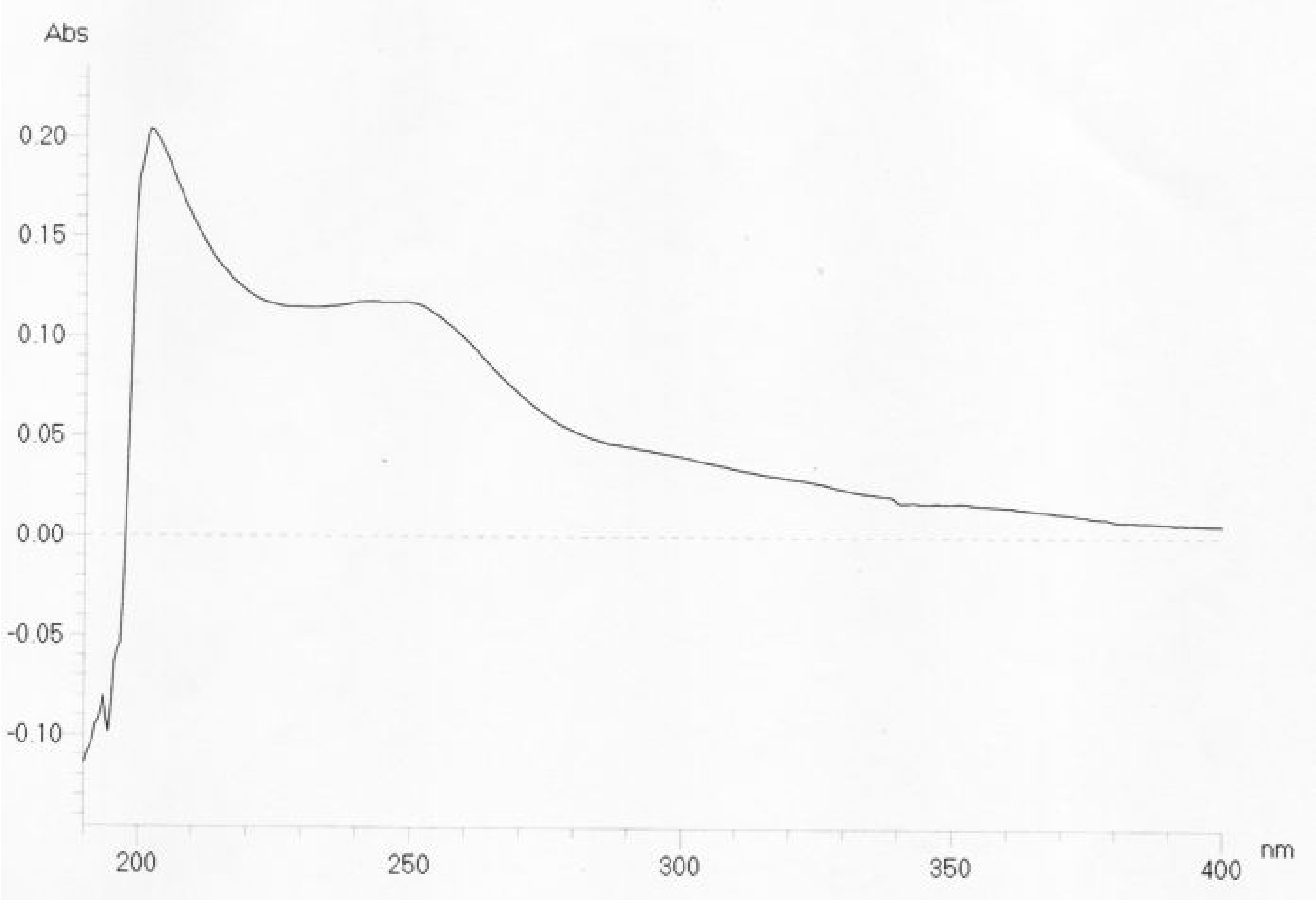

Fig. S52. UV spectrum of **4**

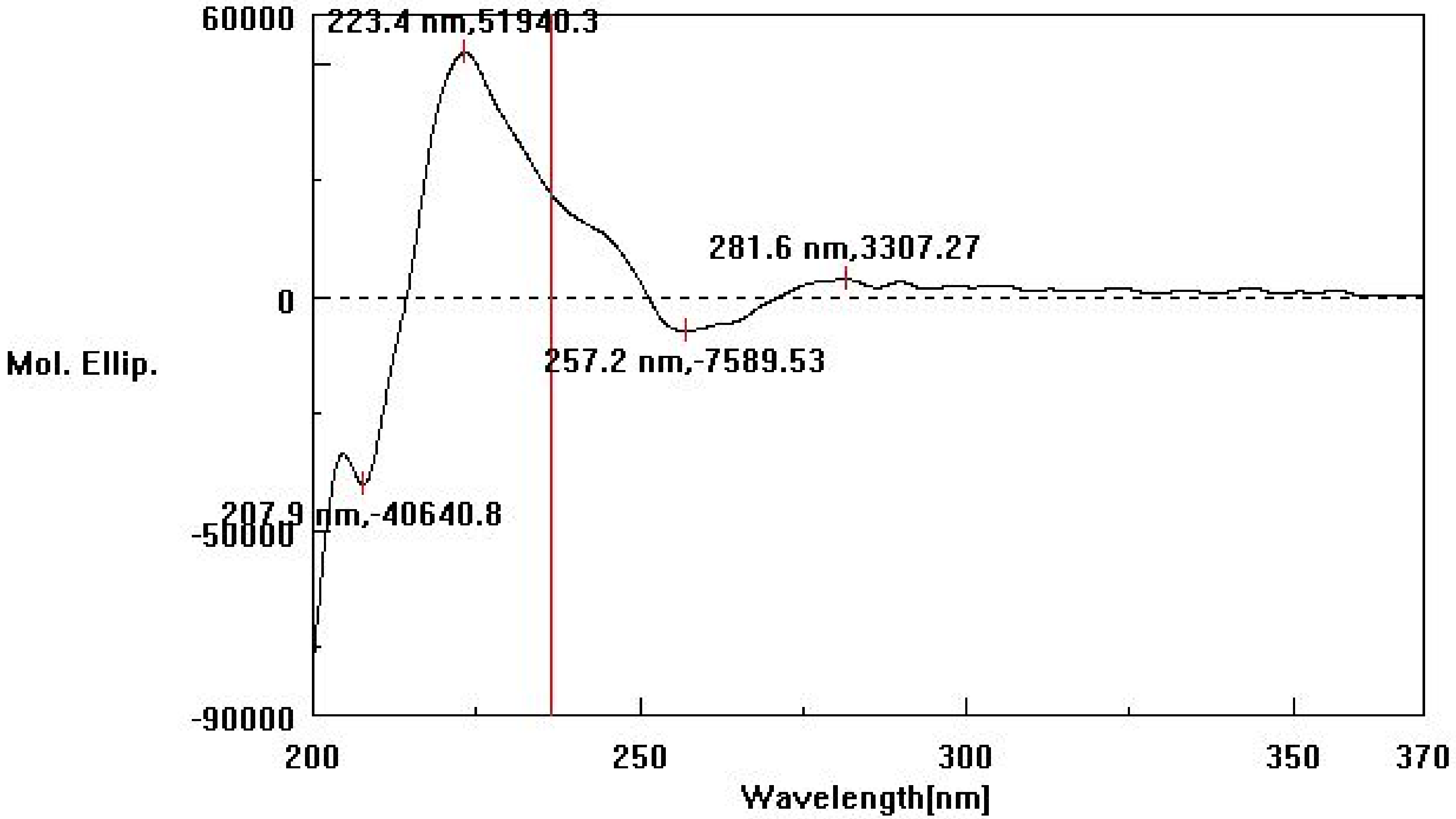

Fig. S53. CD spectrum of **4**

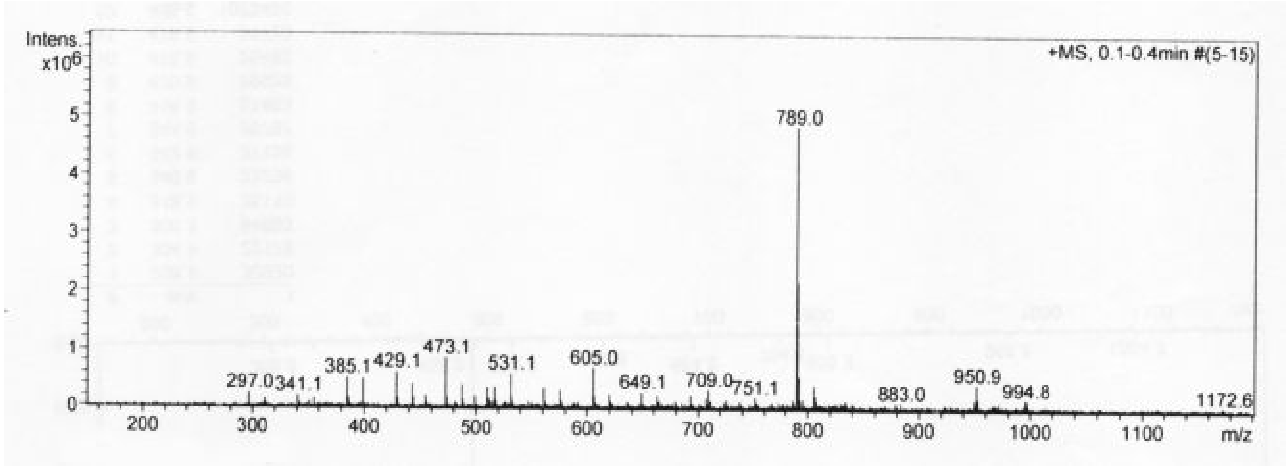

Fig. S54. MS spectrum of **4** (positive mode)

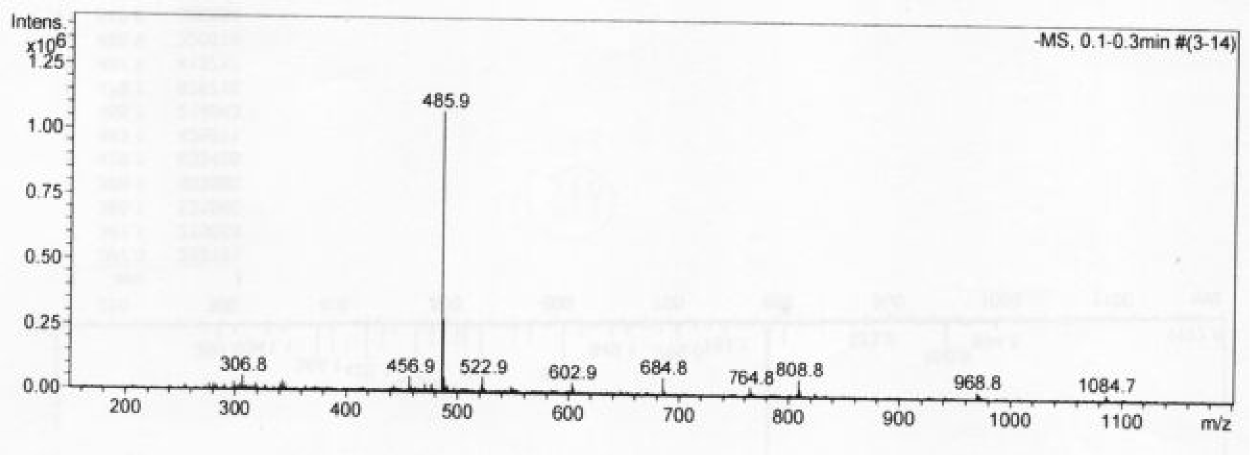

Fig. S55. MS spectrum of **4** (negative mode)

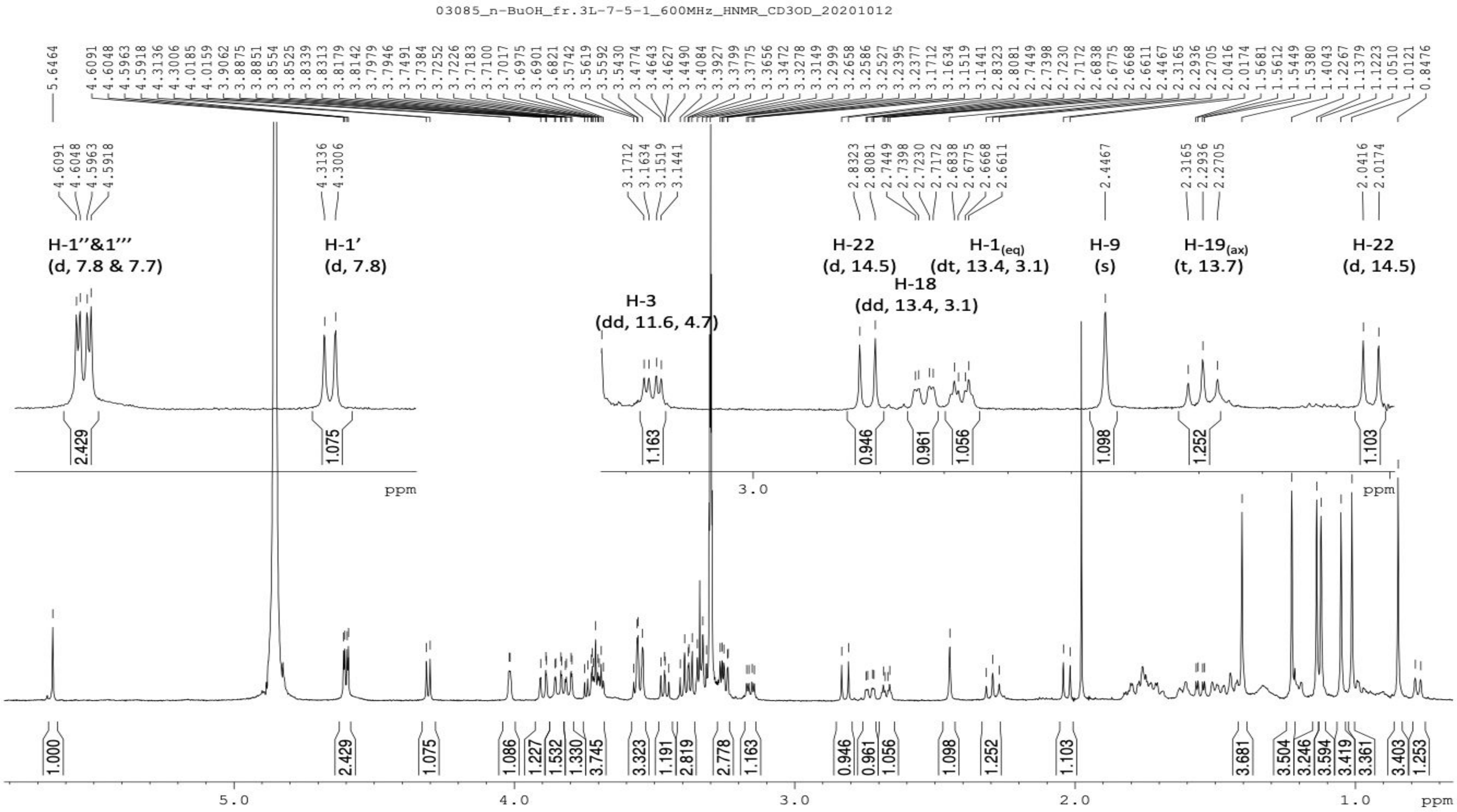

Fig. S56. ^{1}H NMR spectrum of **5** (CD_3OD, 600 MHz)

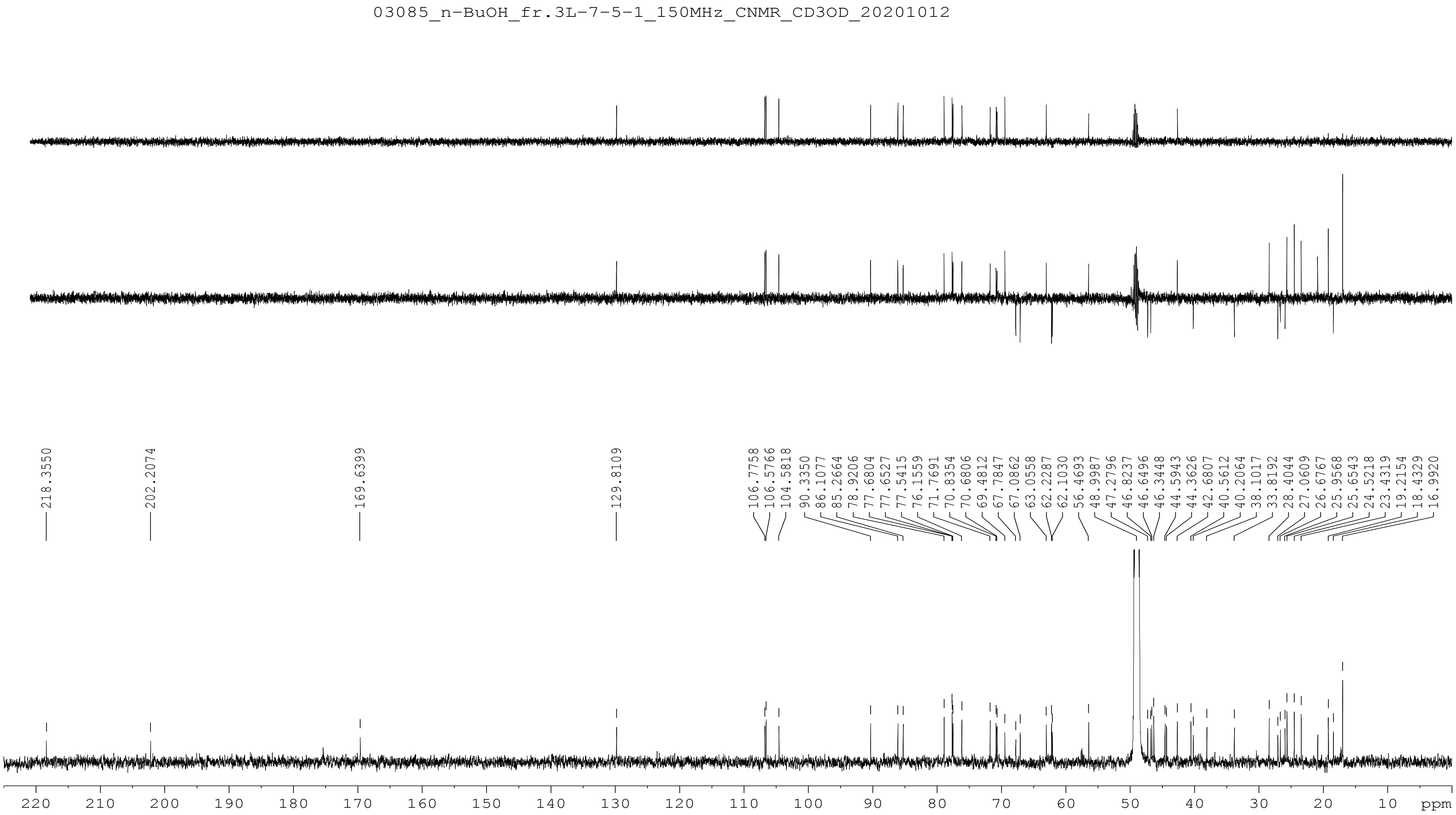

Fig. S57. ^{13}C NMR spectrum of **5** (BBD, bot.; DEPT-135, mid.; DEPT-90, top) (CD_3OD, 150 MHz)

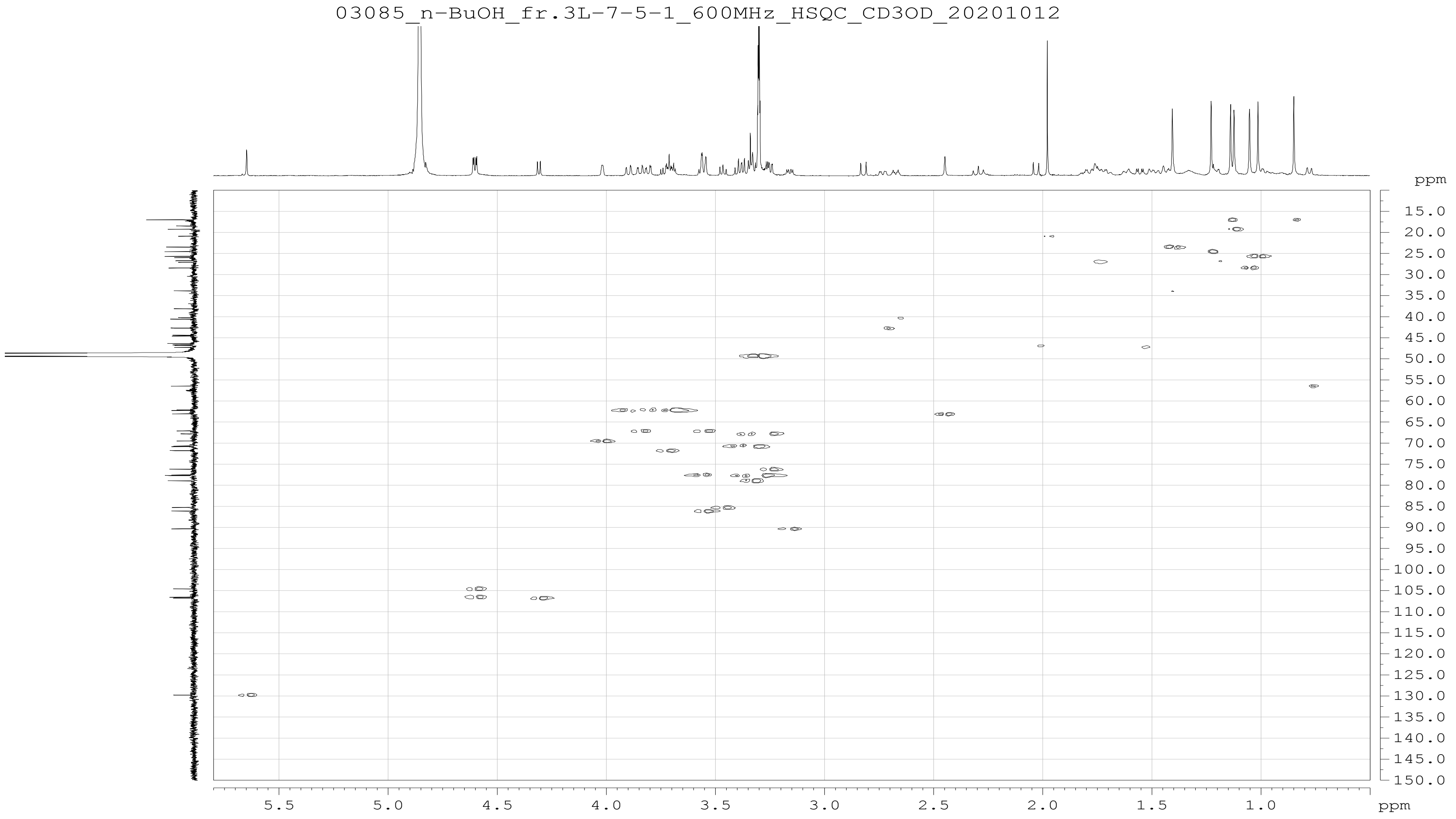

Fig. S58. HSQC spectrum of **5** (CD_3OD, 600 MHz)

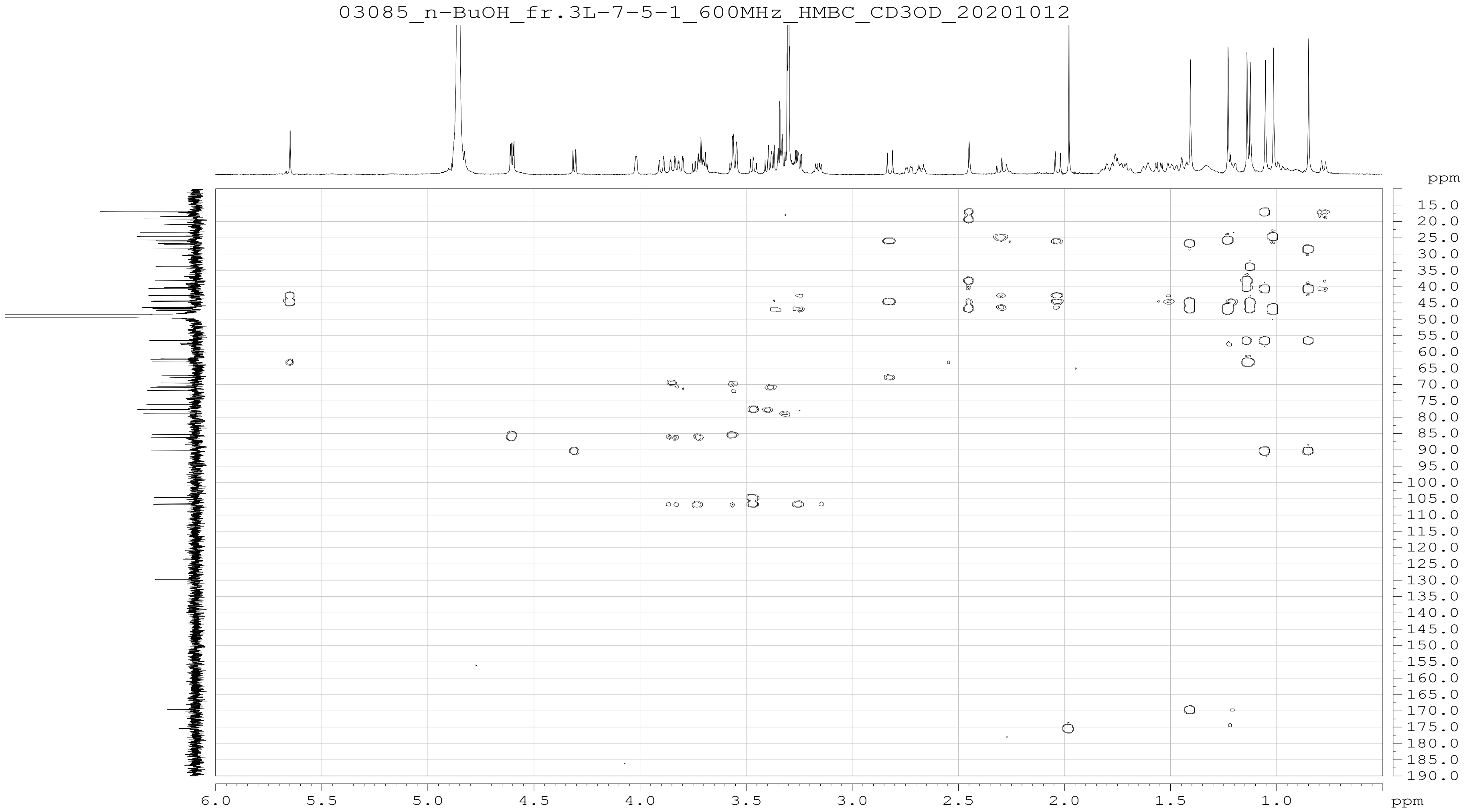

Fig. S59. HMBC spectrum of **5** (CD_3OD, 600 MHz)

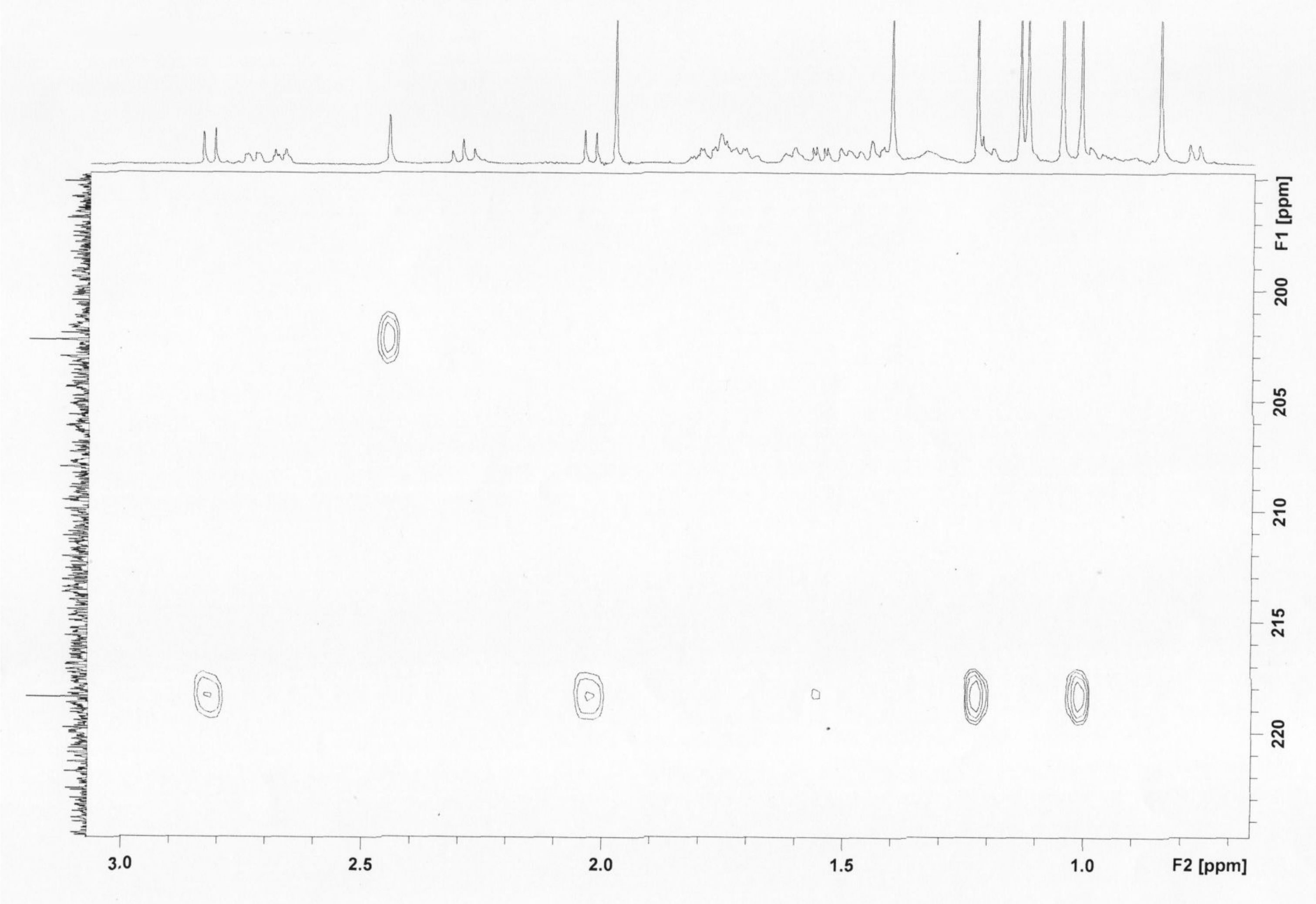

Fig. S60. HMBC spectrum of **5** (CD_3OD, 600 MHz)

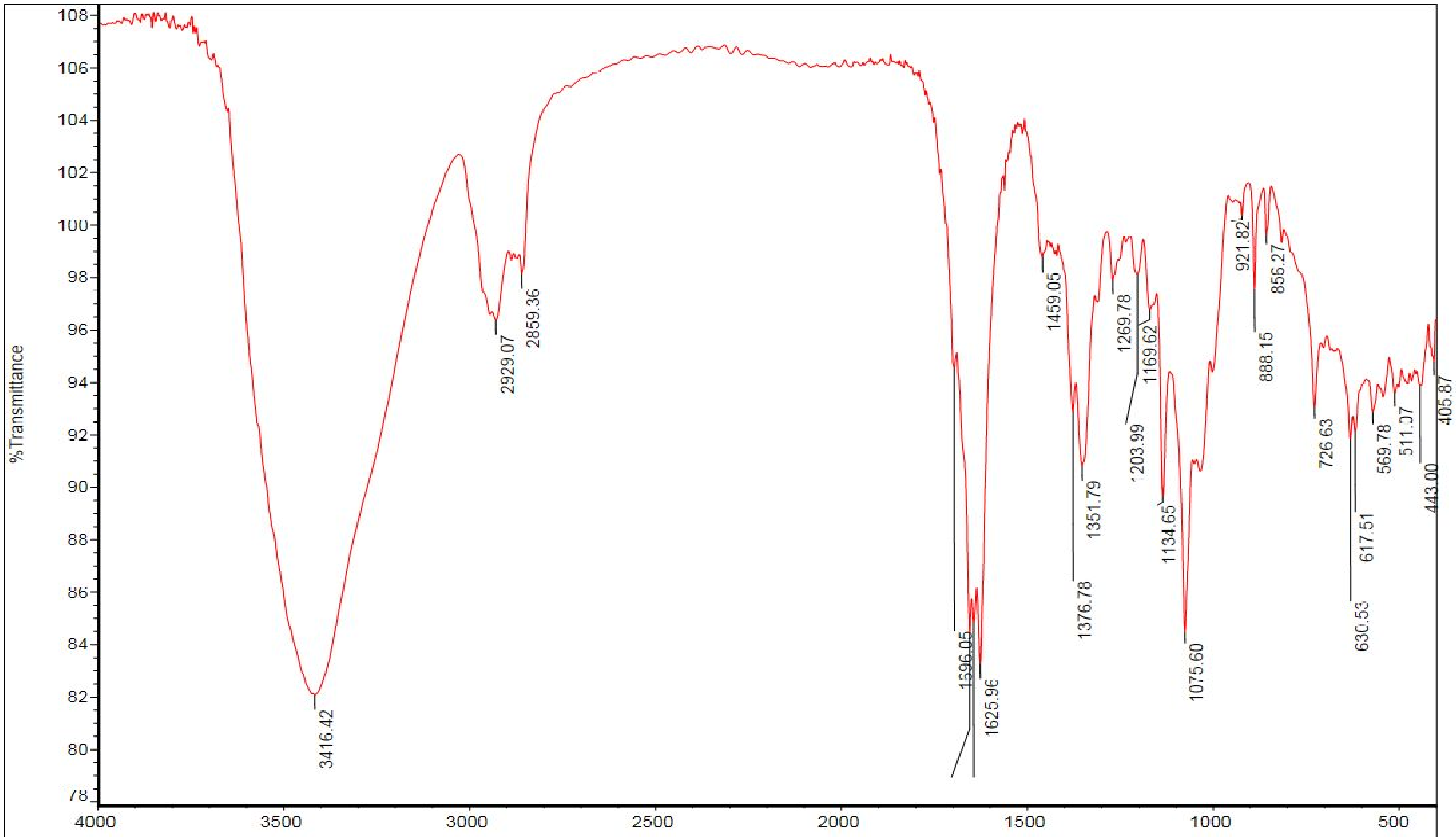

Fig. S61. IR spectrum of **5**

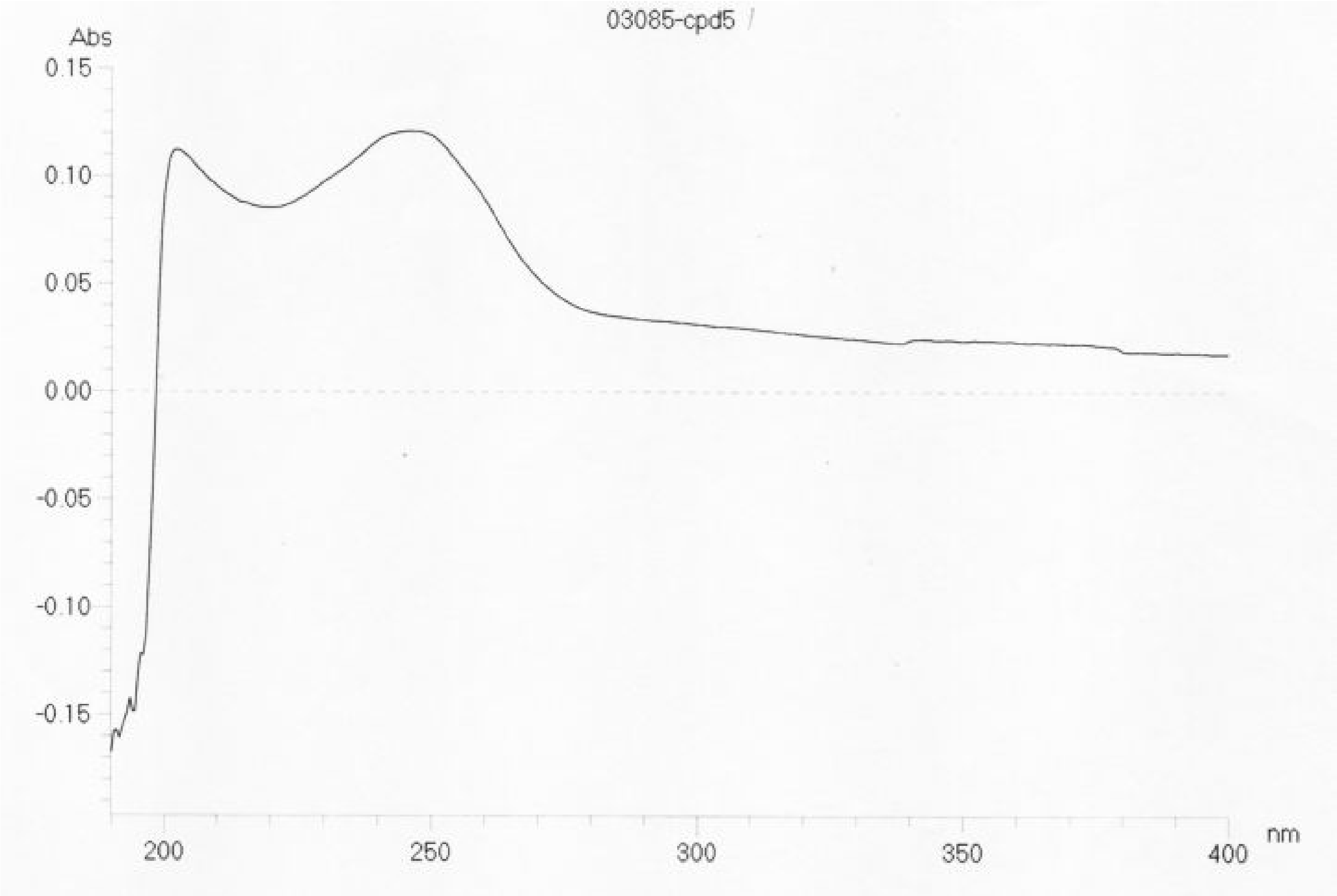

Fig. S62. UV spectrum of **5**

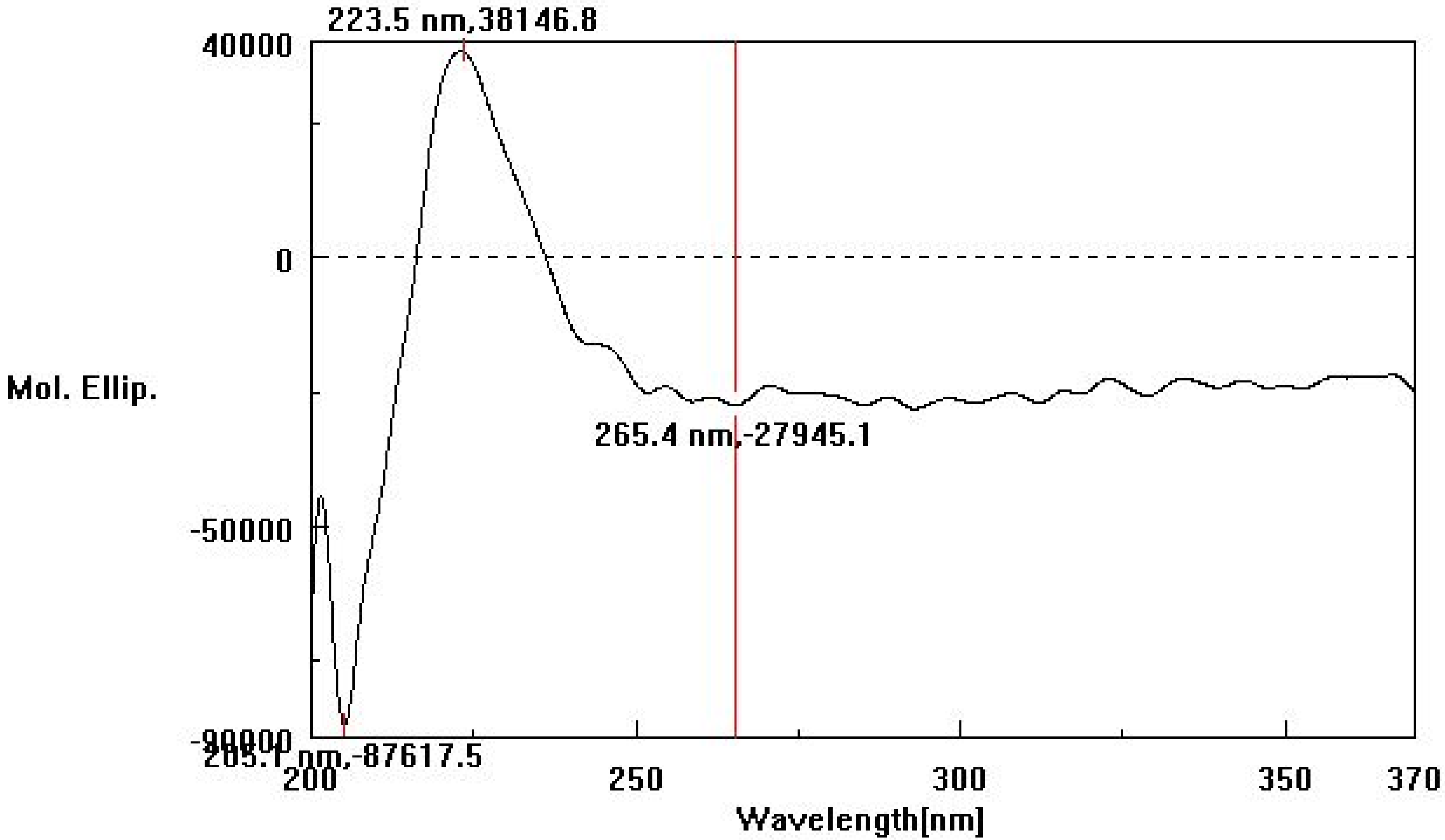

Fig. S63. CD spectrum of **5**

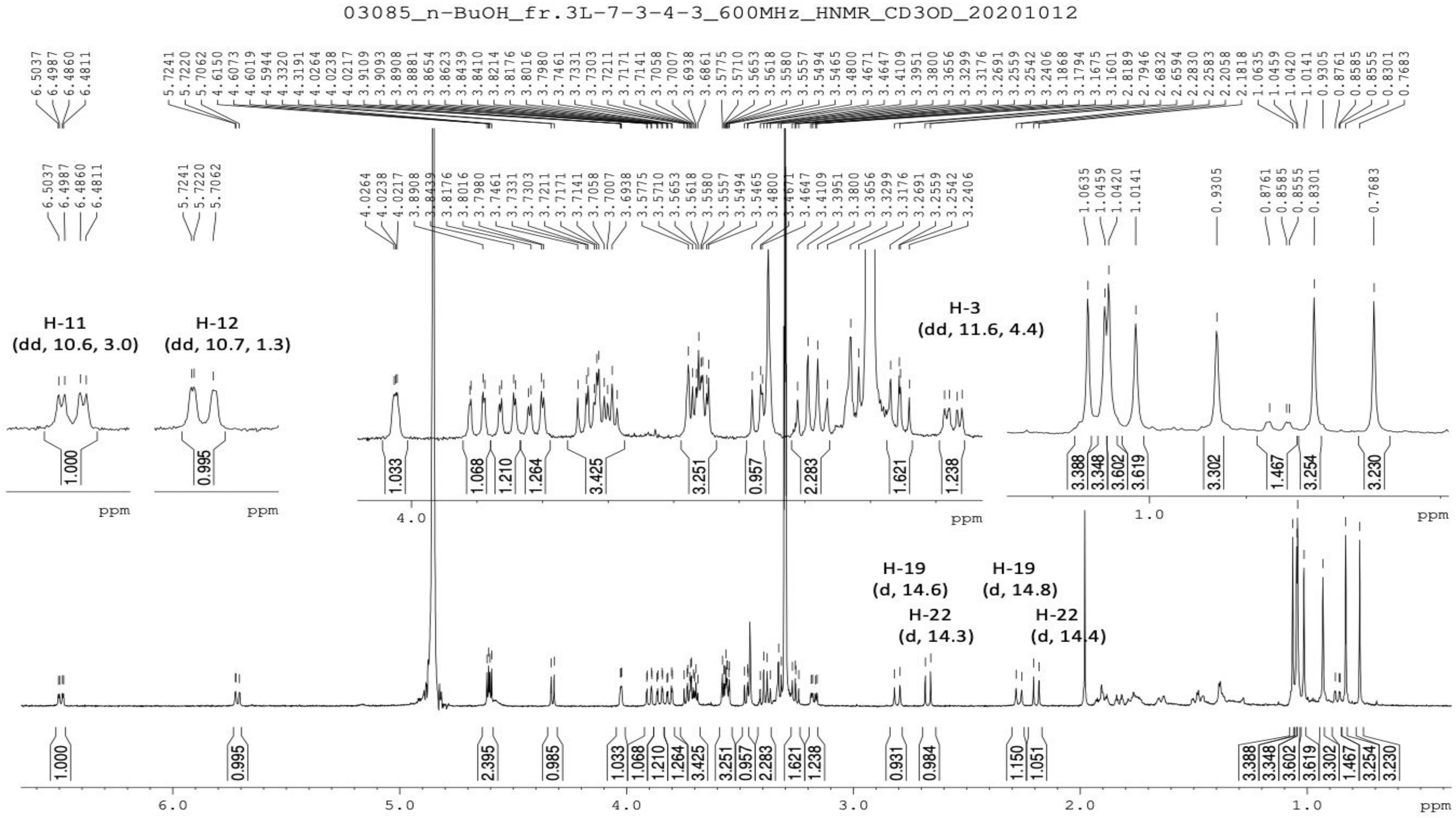

Fig. S64. ^{1}H NMR spectrum of **6** (CD_3OD, 600 MHz)

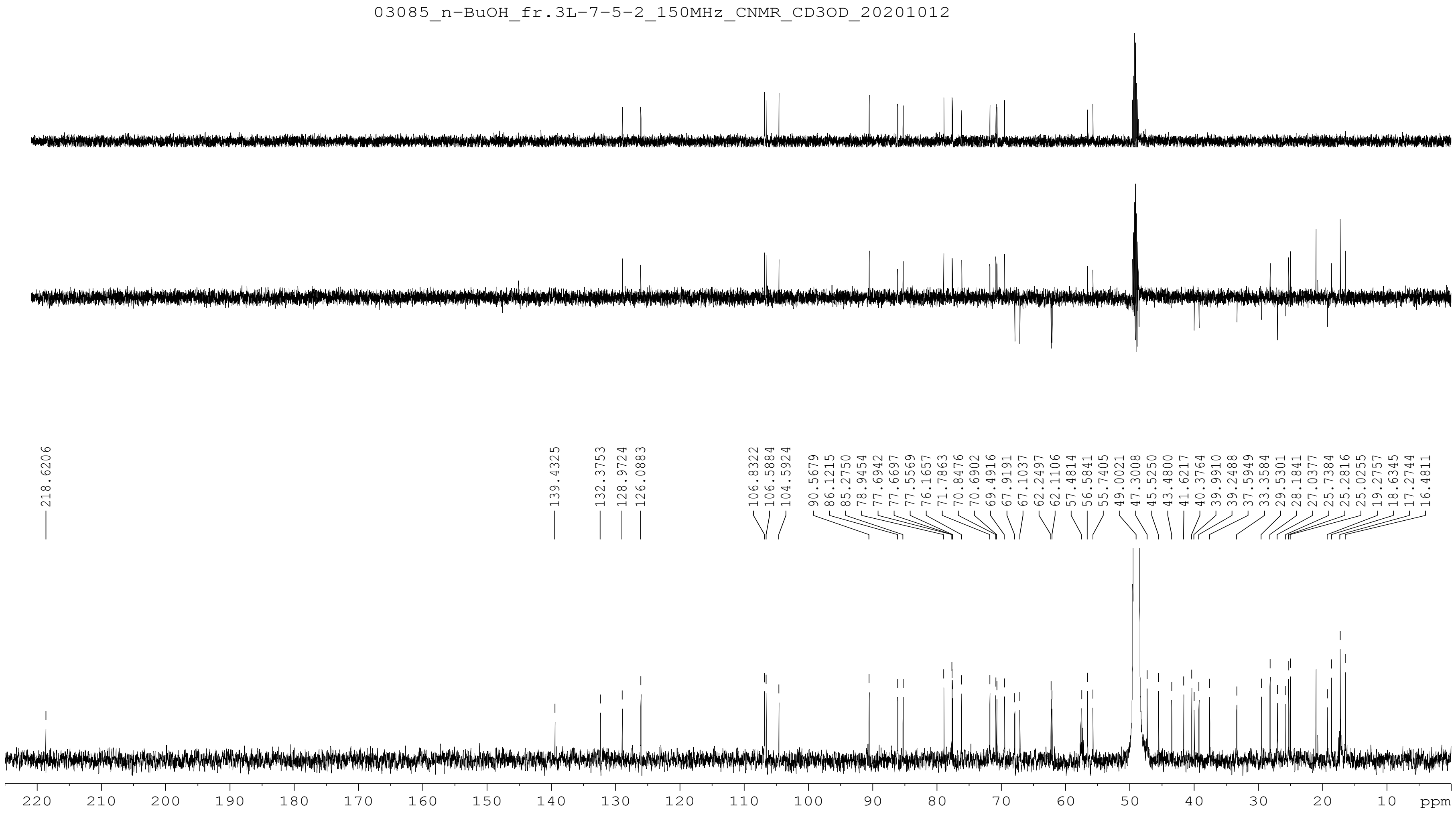

Fig. S65. ^{13}C NMR spectrum of **6** (BBD, bot.; DEPT-135, mid.; DEPT-90, top) (CD_3OD, 150 MHz)

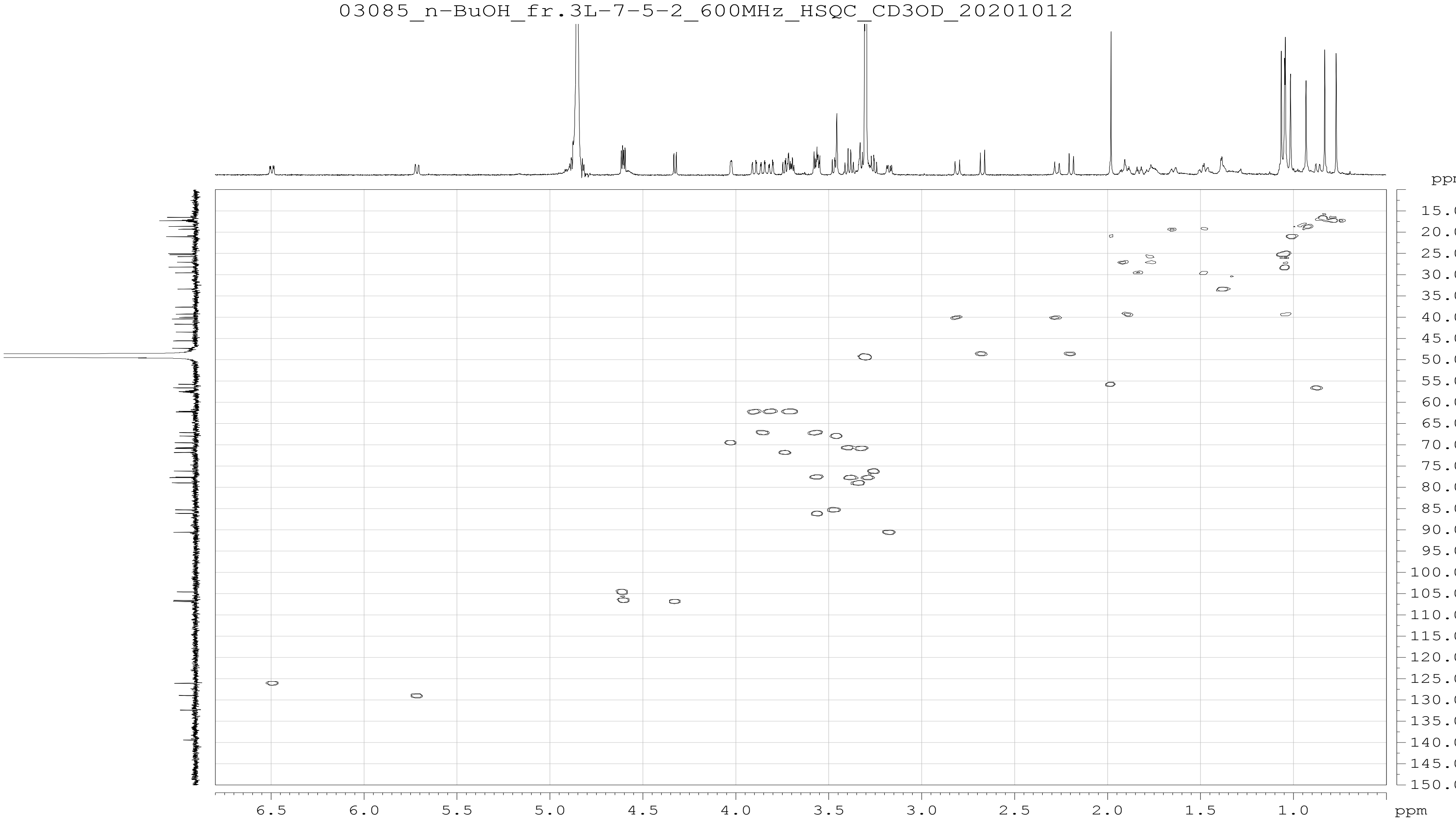

Fig. S66. HSQC spectrum of **6** (CD_3OD, 600 MHz)

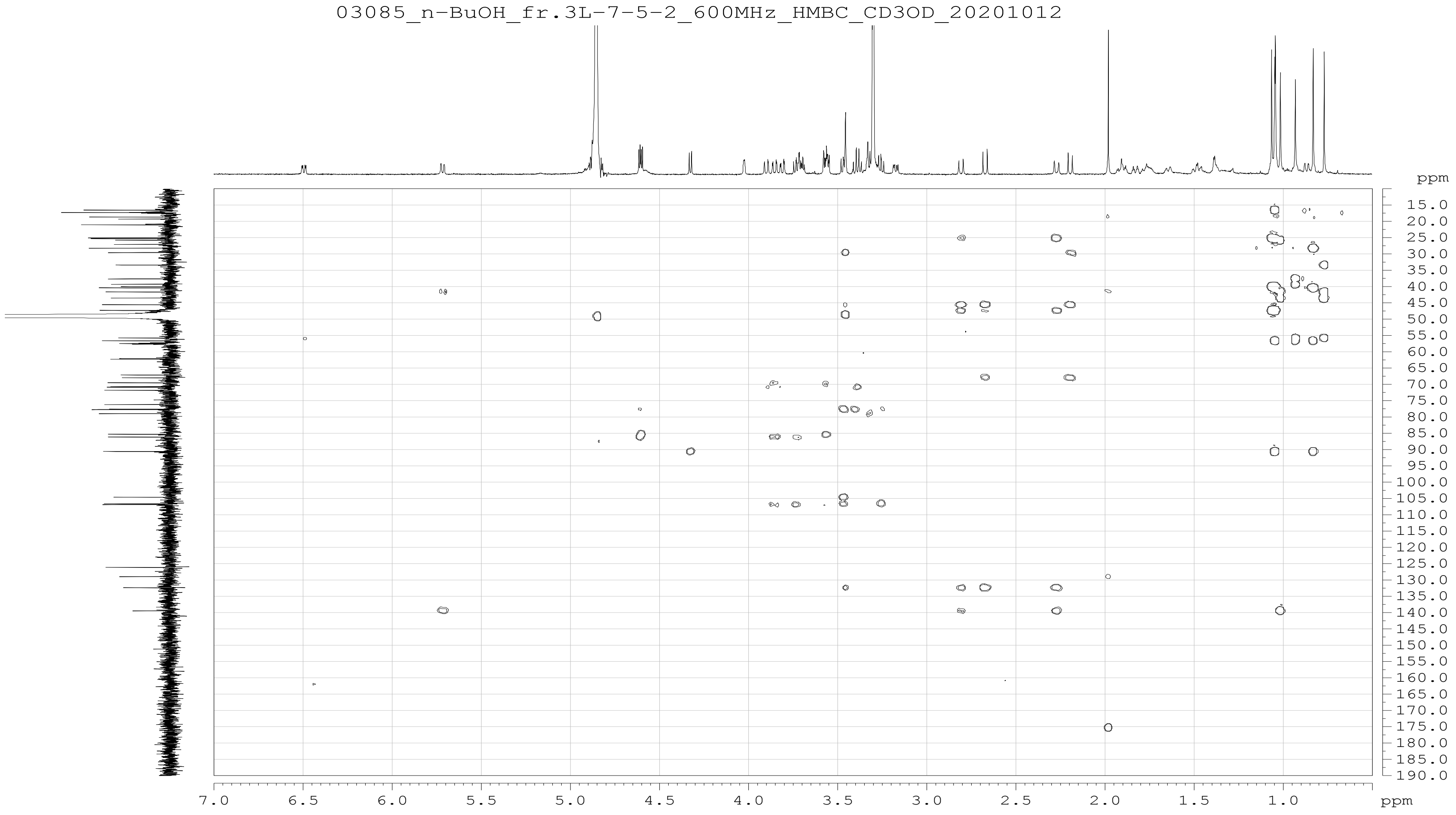

Fig. S67. HMBC spectrum of **6** (CD_3OD, 600 MHz)

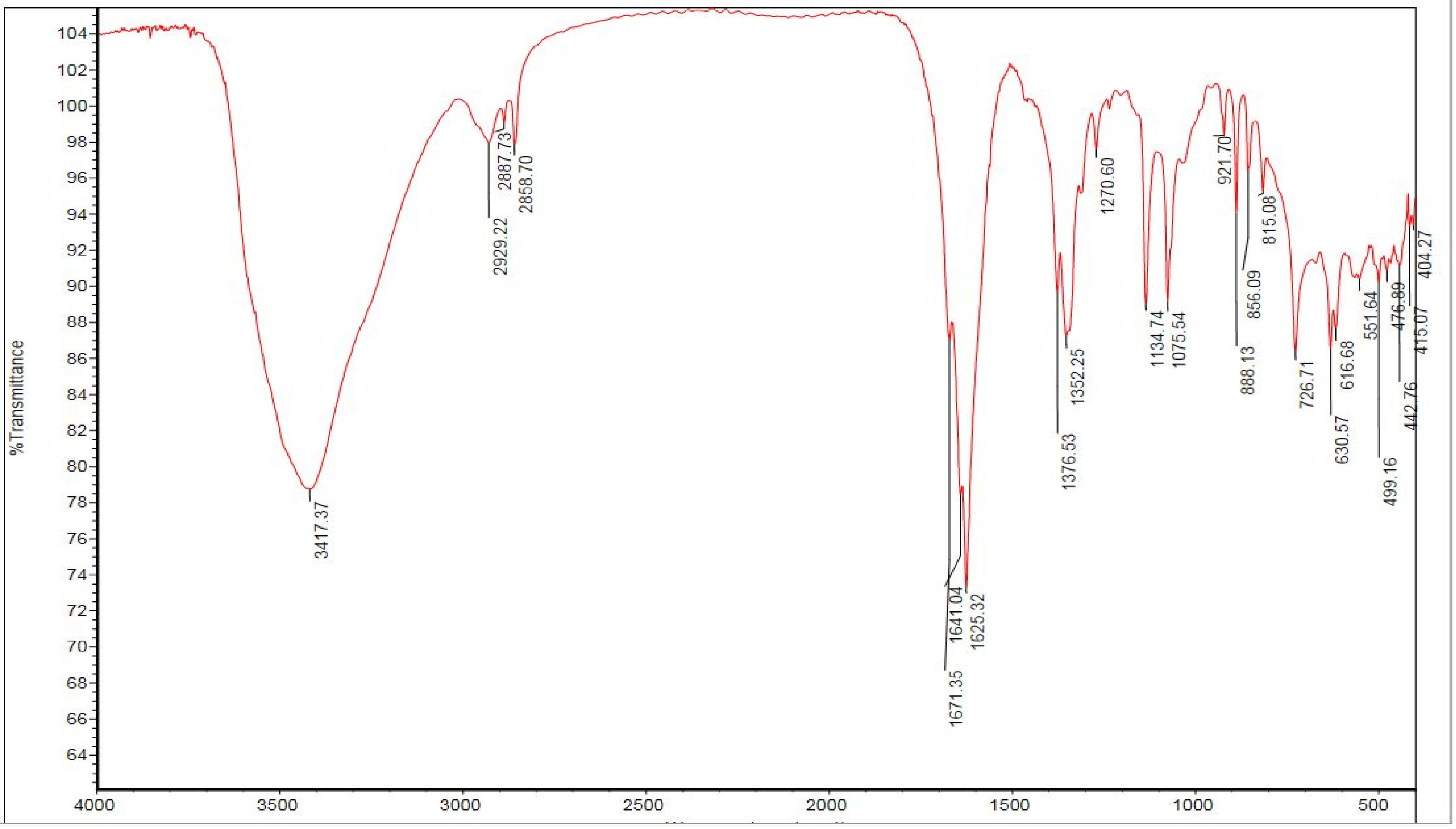

Fig. S68. IR spectrum of **6**

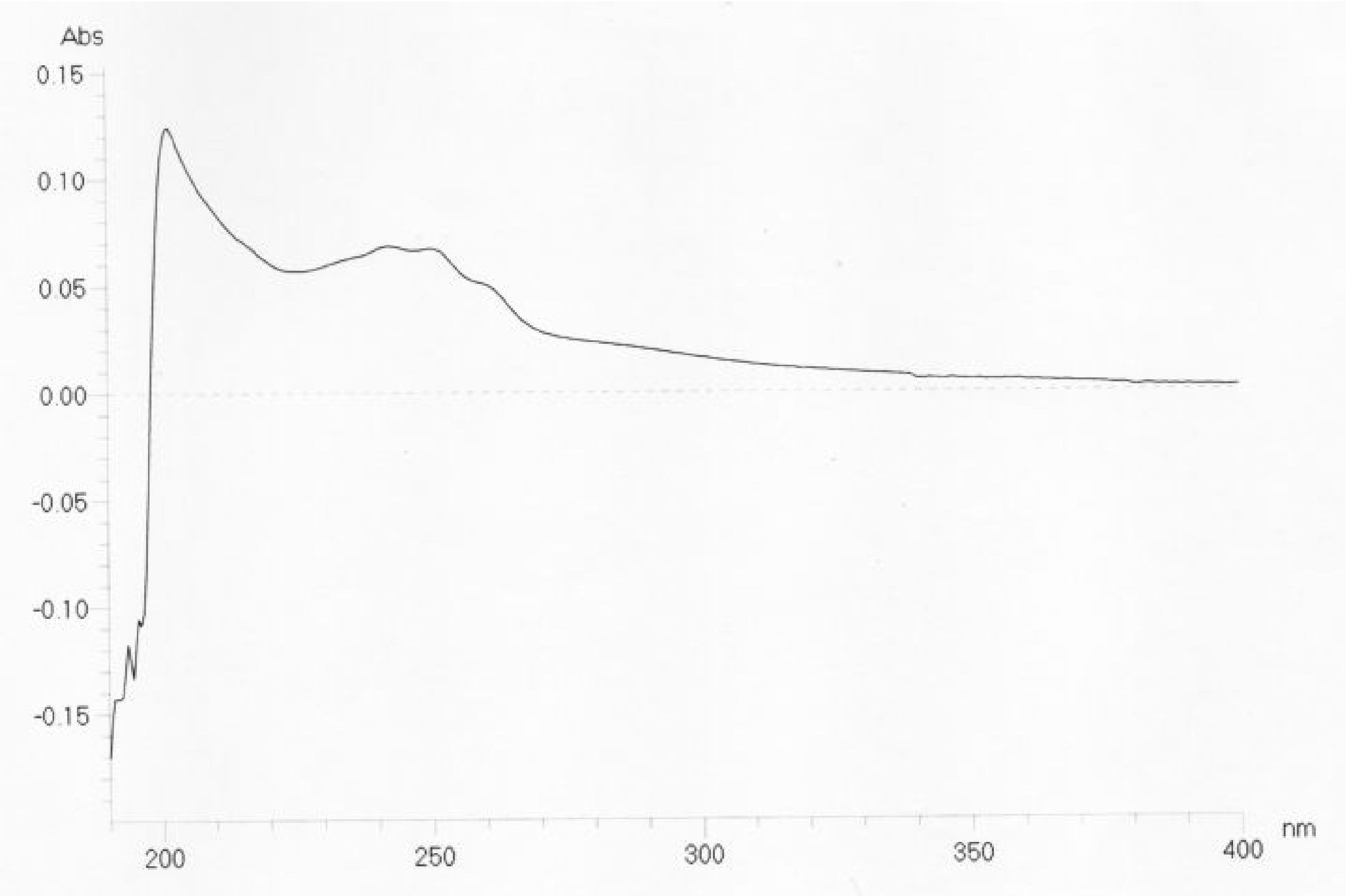

Fig. S69. UV spectrum of **6**

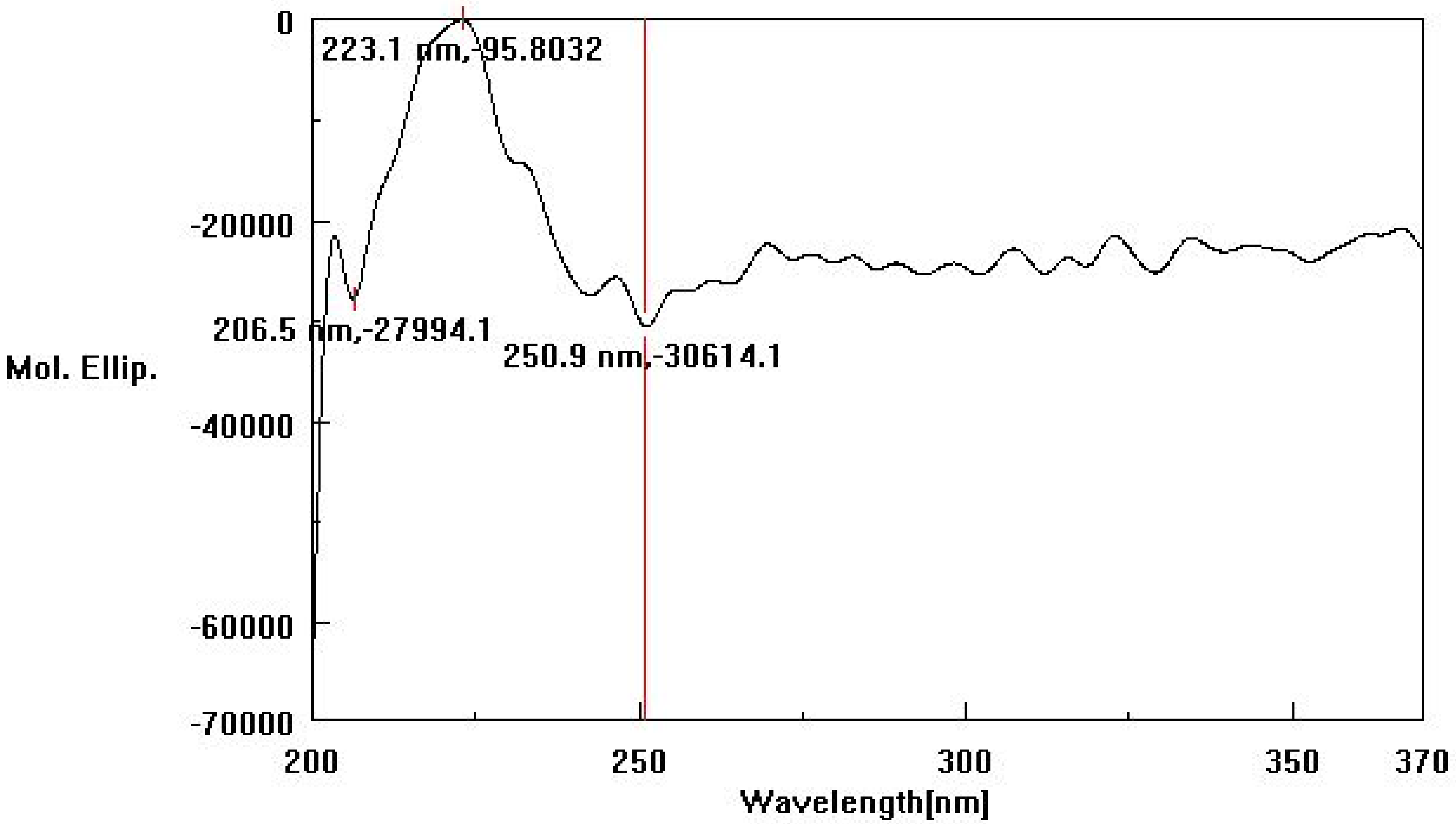

Fig. S70. CD spectrum of **6**

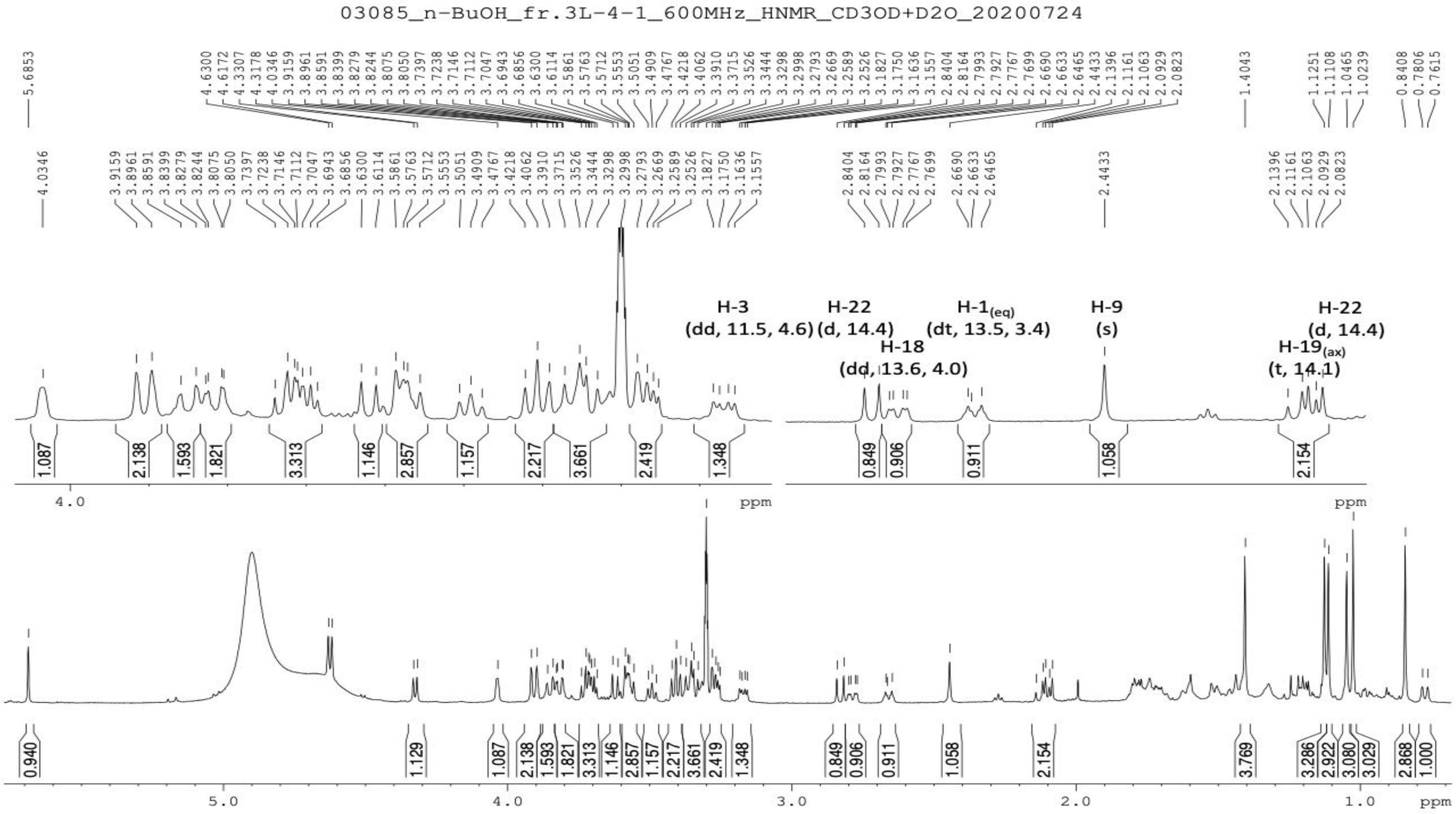

Fig. S71. ^{1}H NMR spectrum of **7** (CD_3OD+D_2O, 600 MHz)

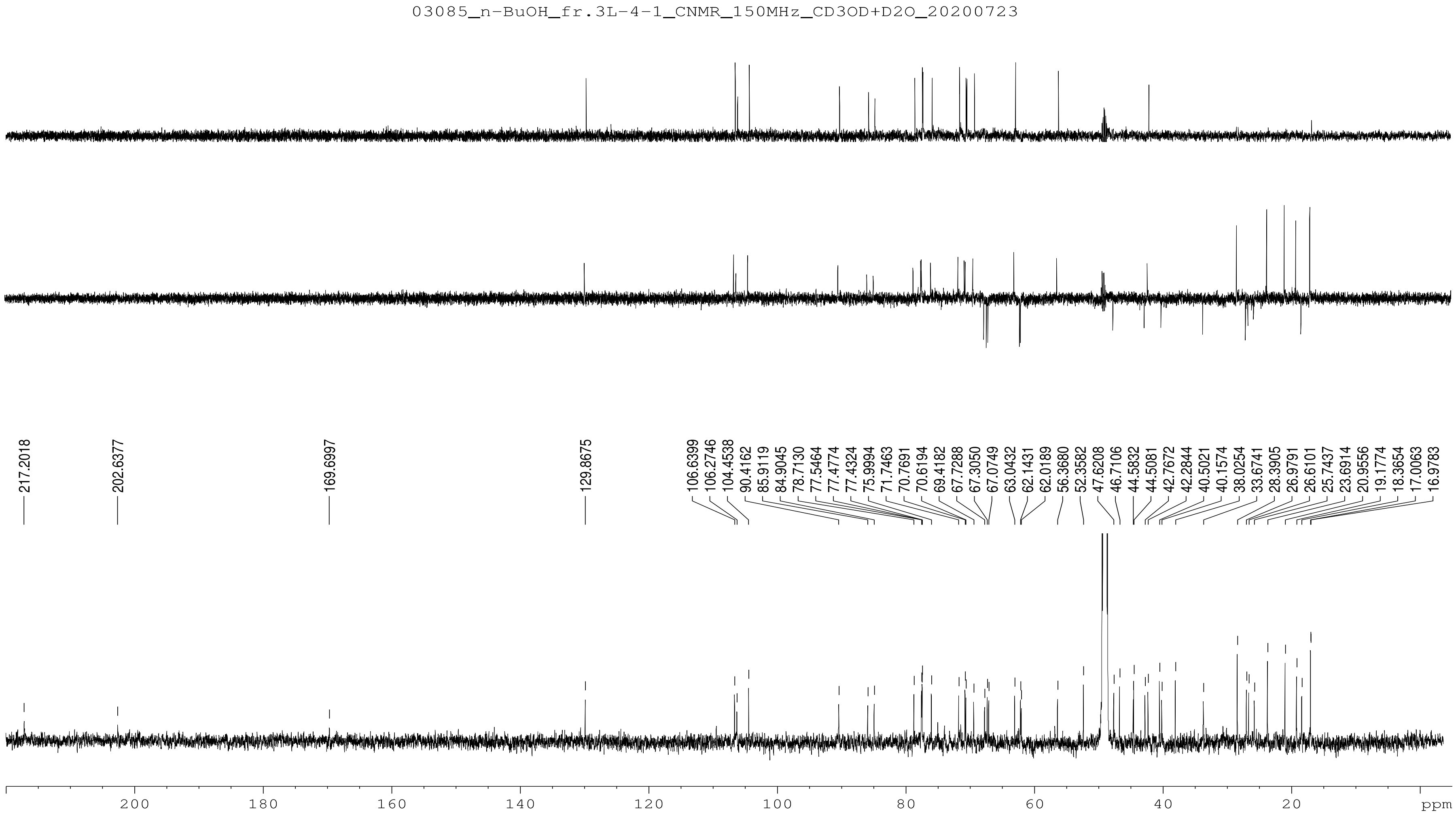

Fig. S72. ^{13}C NMR spectrum of **7** (BBD, bot.; DEPT-135, mid.; DEPT-90, top) (CD_3OD+D_2O, 150 MHz)

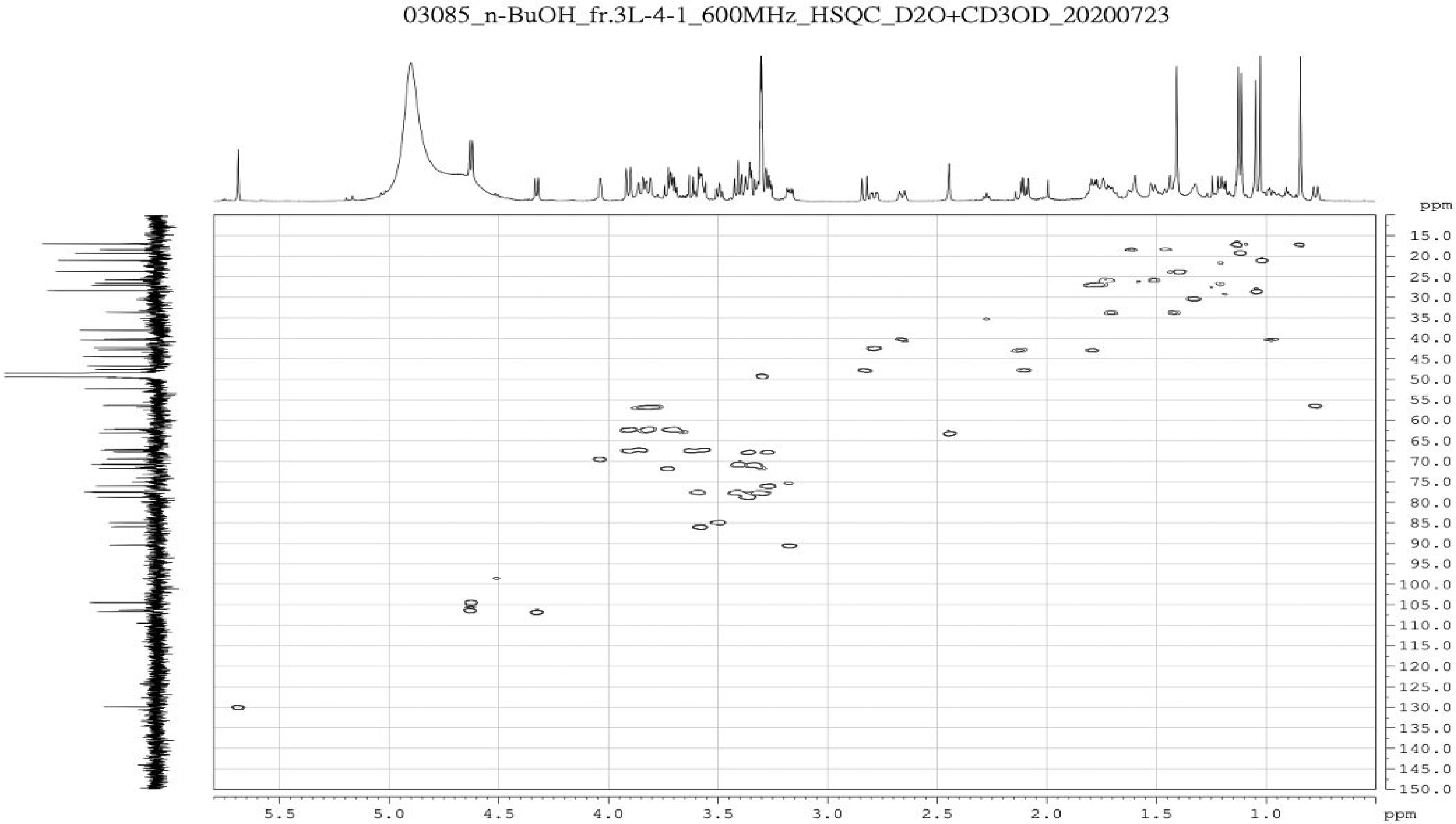

Fig. S73. HSQC spectrum of **7** (CD_3OD+D_2O, 600 MHz)

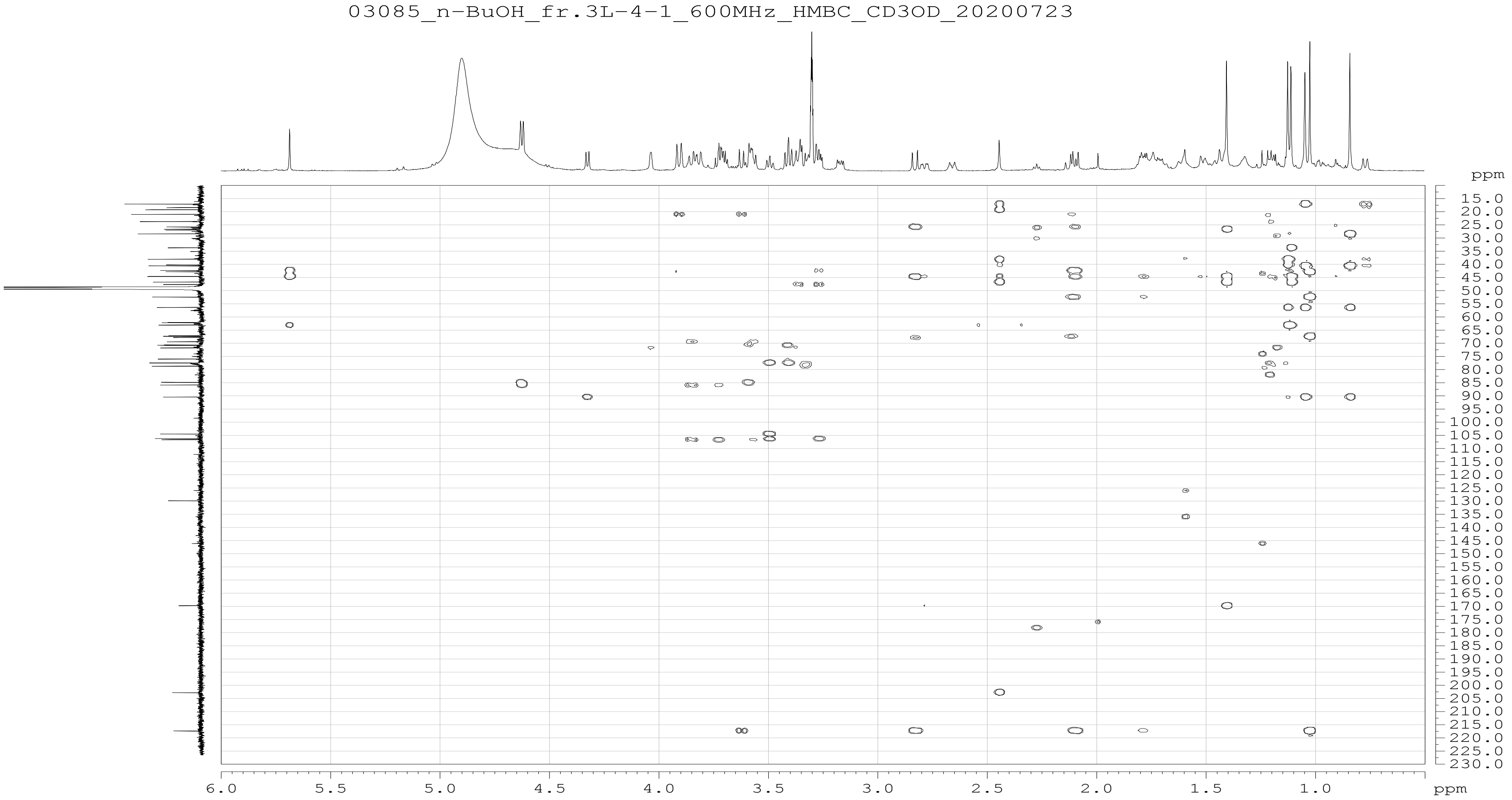

Fig. S74. HMBC spectrum of **7** (CD_3OD+D_2O, 600 MHz)

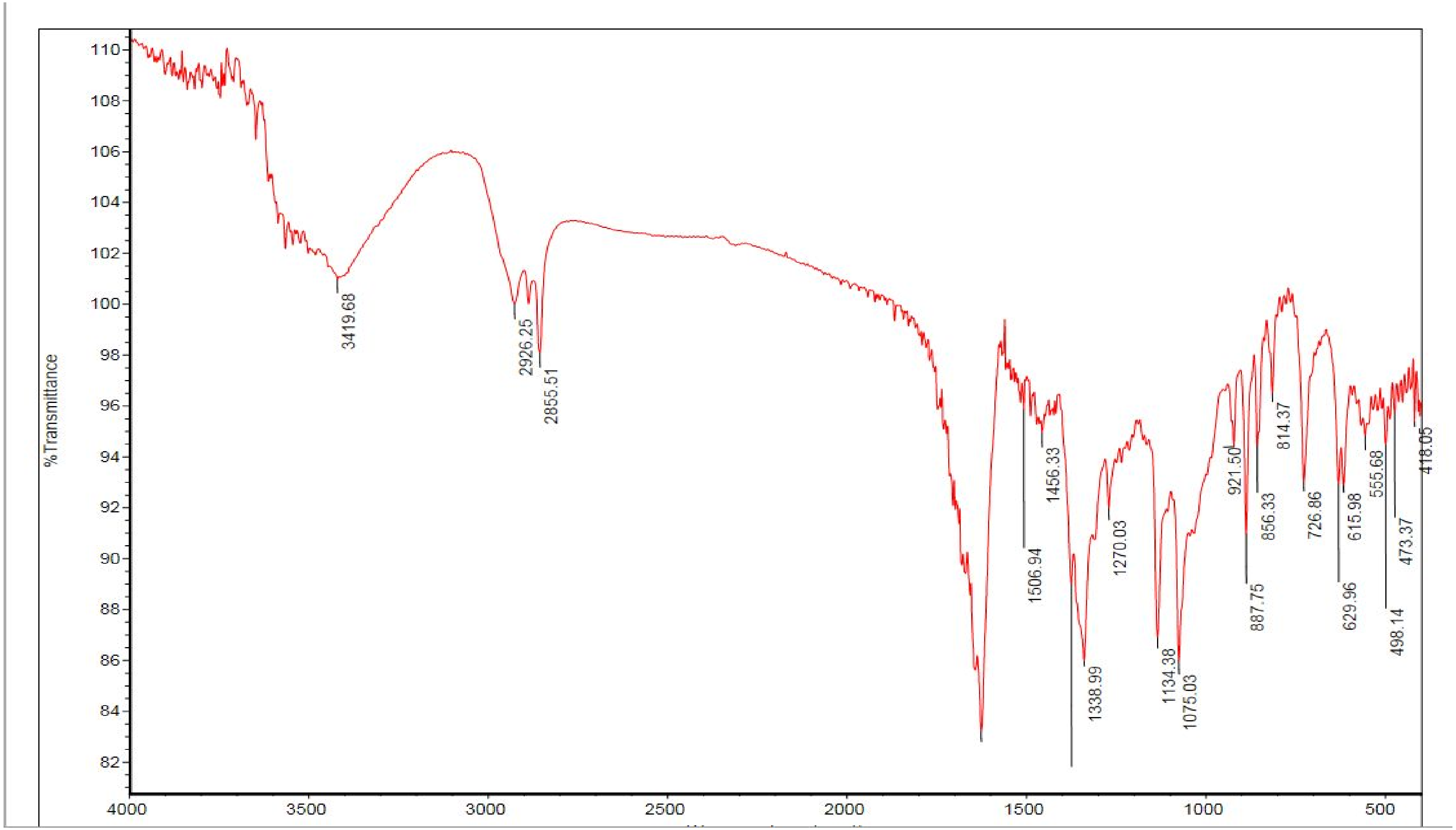

Fig. S75. IR spectrum of **7**

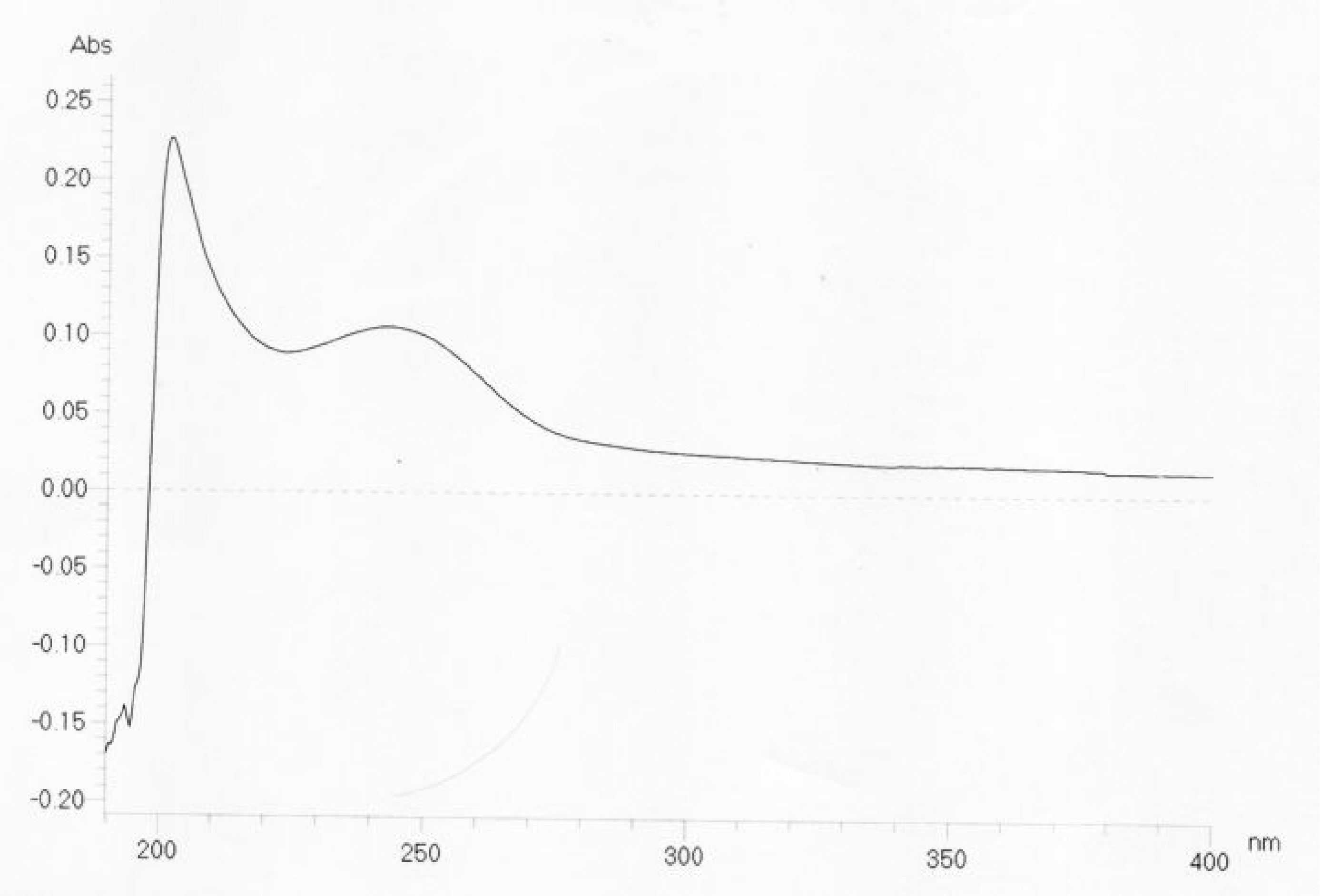

Fig. S76. UV spectrum of **7**

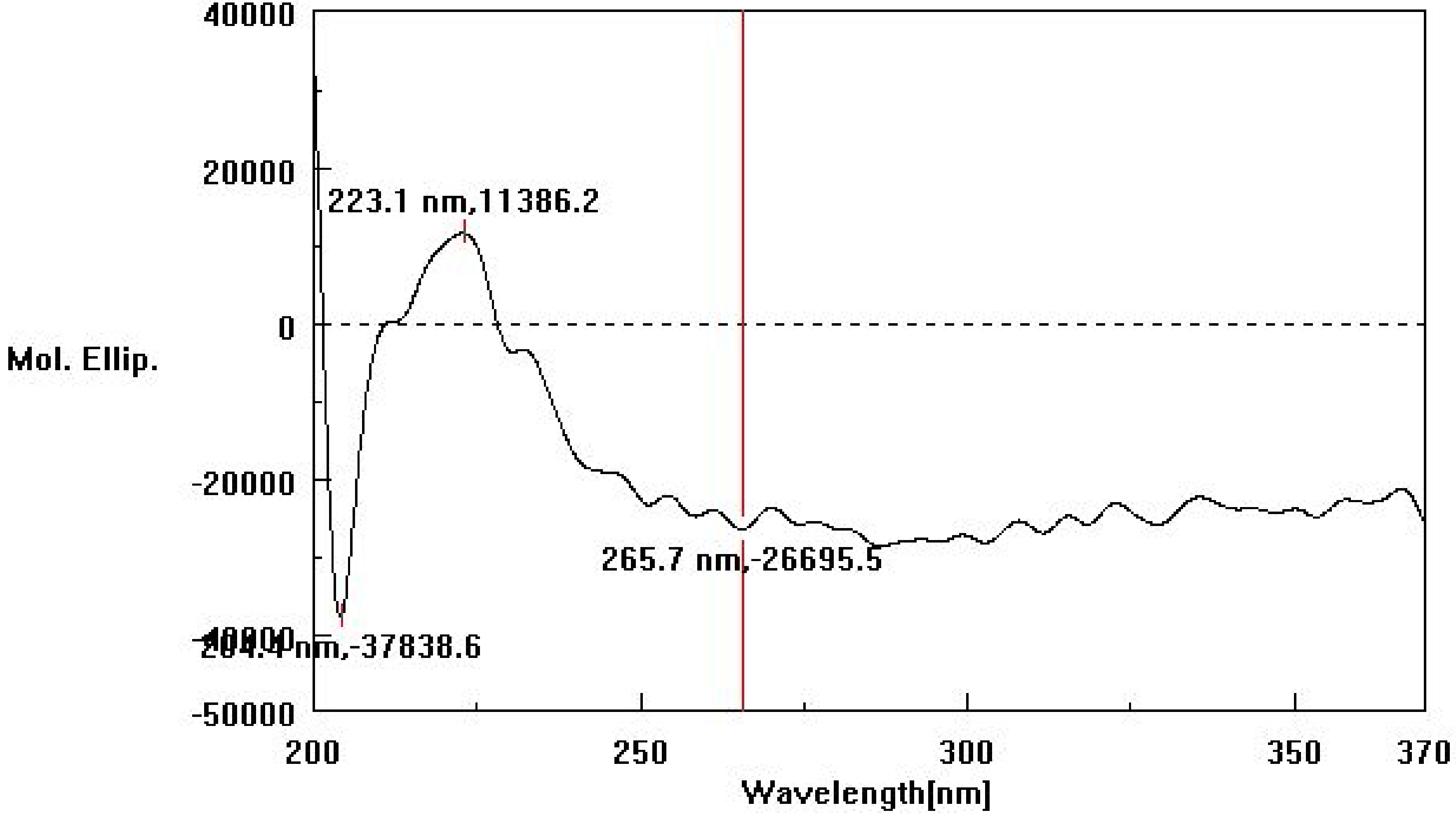

Fig. S77. CD spectrum of **7**

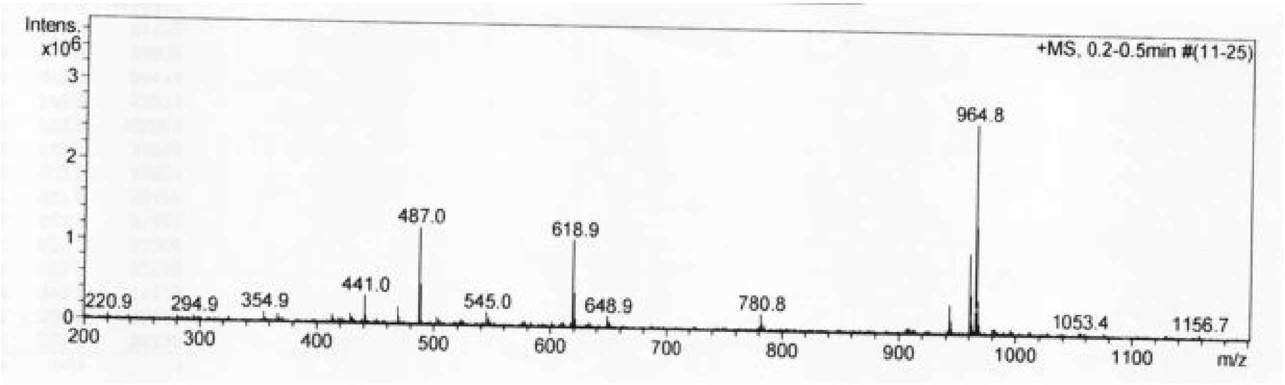

Fig. S78. MS spectrum of **7** (positive mode)

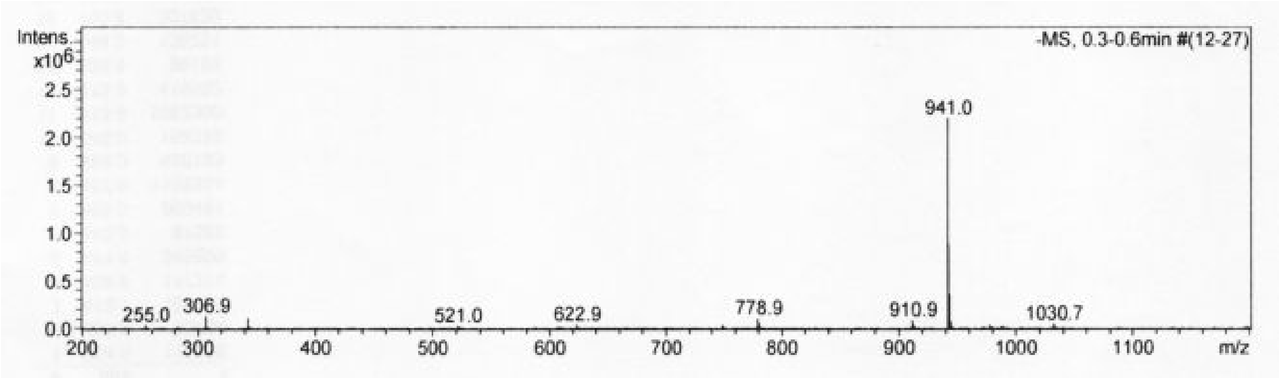

Fig. S79. MS spectrum of **7** (negative mode)

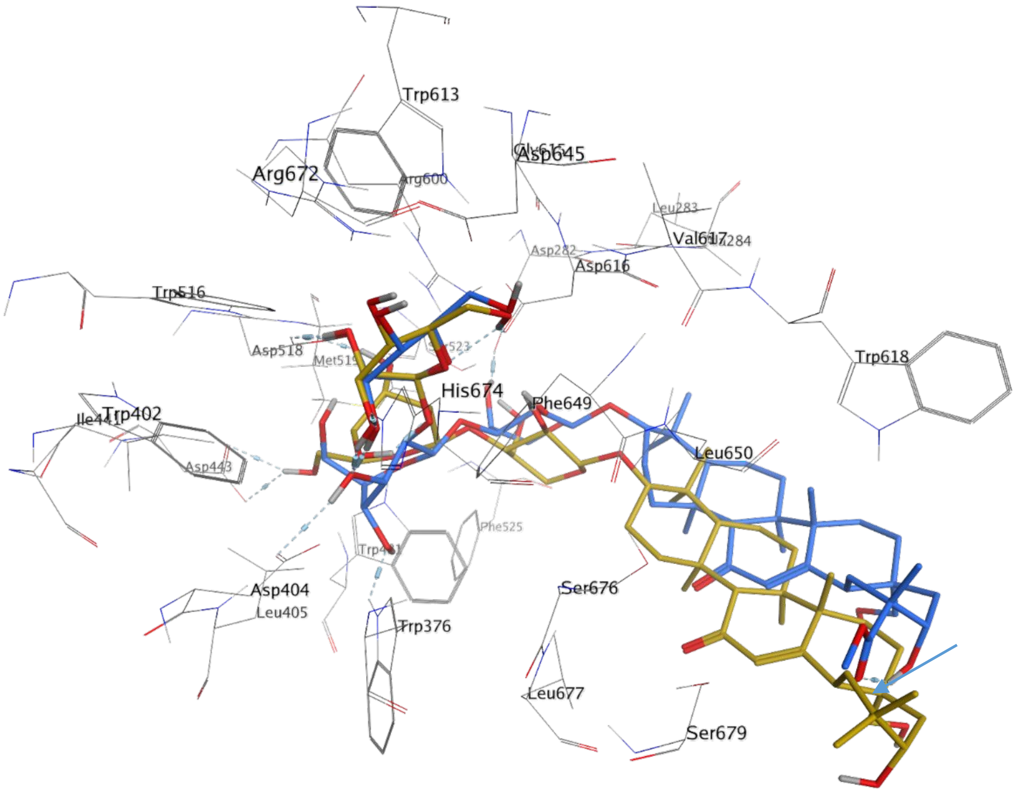

Fig. S80. Depiction of **1** (blue) vs. **2** docked with the AG; intramolecular H-bond: →

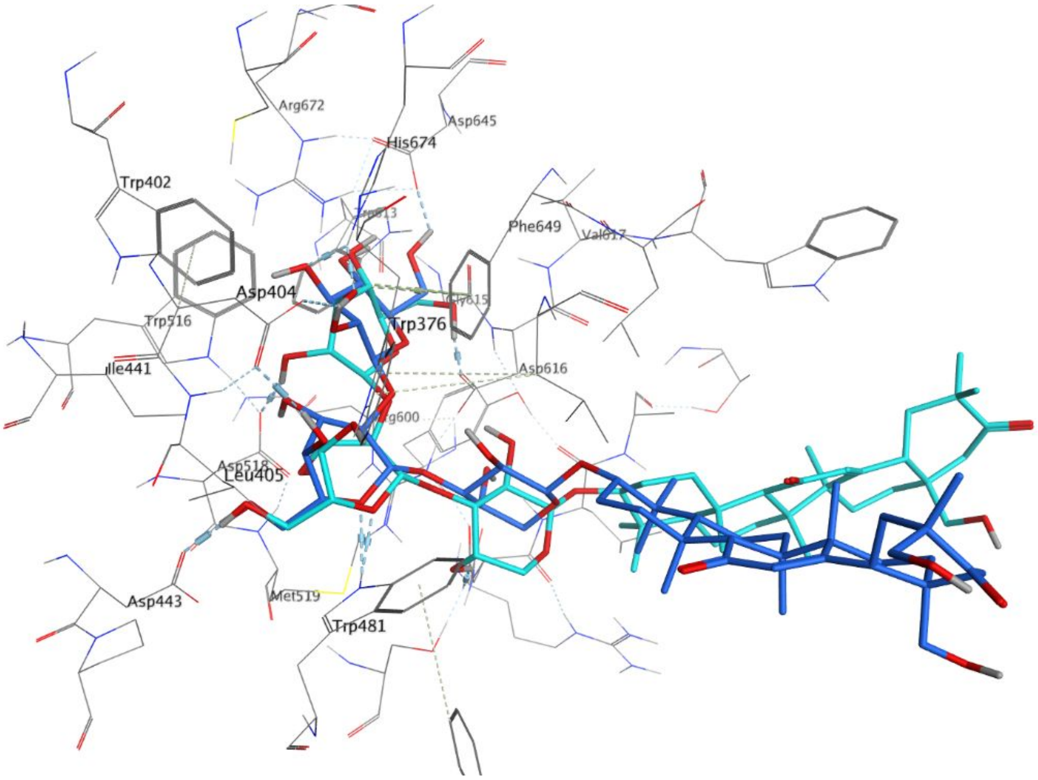

Fig. S81. Depiction of **5** (cyan) vs. **7** docked with the AG
